# Supplementary material for: Adaptive multi-temperature control for transport and storage containers enabled by phase-change materials
Source: Nat Commun. 2023 Sep 6;14:5449. doi: 10.1038/s41467-023-40988-2 (PMC10482904; doi:10.1038/s41467-023-40988-2)
Supplement: Supplementary file 1 — Supplementary Information [file 41467_2023_40988_MOESM1_ESM.pdf]

Supplementary Information

Adaptive multi-temperature control for transport and storage containers enabled by phase-change materials

Xinchen Zhou[1,2,3], Xiang Xu[4,5], and Jiping Huang[1,2,3*]

[1]*Department of Physics, Fudan University, Shanghai 200438, China*.

[2]*State Key Laboratory of Surface Physics, Fudan University, Shanghai 200438, China*.

[3]*Key Laboratory of Micro and Nano Photonic Structures (MOE), Fudan University, Shanghai 200438, China*.

[4]*Key Lab of Smart Prevention and Mitigation of Civil Engineering Disasters of the Ministry of Industry and Information Technology, Harbin Institute of Technology, Harbin 150090, China*.

[5]*Key Lab of Structures Dynamic Behavior and Control of the Ministry of Education, Harbin Institute of Technology, Harbin 150090, China*.

[*]Correspondence to: jphuang@fudan.edu.cn.

This PDF file includes:

Supplementary Notes 1-15

Supplementary Figures 1-30

Supplementary Tables 1-6

Supplementary References

Supplementary Notes

1 Detailed derivation/explanation for the "General theory for AMTC"

1.1 Derivation for Eq. (4)

According to Eq. (3), we obtain

$$q_m = \frac{T_{\mathrm{c},\{m,j\}} - T^{+}_{\mathrm{c},\{m,j\}}}{\left(1/2d_{\mathrm{c},\{m,j\}} - x_{\{m,j\}}\right)/\kappa_{\mathrm{c},\{m,j\}}} \tag{S1a}$$

$$= \frac{T^{+}_{\mathrm{c},\{m,j\}} - T_{\mathrm{b},\{m,j\}}}{d_{\mathrm{r},\{m,j\}}/\kappa_{\mathrm{r},\{m,j\}}}, \tag{S1b}$$

where $T^{+}_{\mathrm{c},\{m,j\}}$ represents the right boundary temperature of $\mathrm{C}_{\{m,j\}}$. From Eq. (S1b)

$$T^{+}_{\mathrm{c},\{m,j\}} = q_m \frac{d_{\mathrm{r},\{m,j\}}}{\kappa_{\mathrm{r},\{m,j\}}} + T_{\mathrm{b},\{m,j\}}. \tag{S2}$$

Substituting Eq. (S2) into Eq. (S1a) and rearranging, we finally arrive at Eq. (4).

1.2 Derivation for Eq. (5b)

The core idea behind deriving Eq. (5b) is to establish a relationship between the local system and the global systems. We define the temperature range ratio of these two systems as

$$\Lambda_{\{m,j\}} = \frac{T_{\mathrm{a},\{m,j\}} - T_{\mathrm{b},\{m,j\}}}{T_{\mathrm{A}} - T_{\mathrm{B}}}. \tag{S3}$$

Considering the relationships among $T_{\mathrm{a},\{m,j\}}$, $T_{\mathrm{b},\{m,j\}}$, T_{A}, and T_{B}, we have

$$\begin{cases} T_{\mathrm{b},\{m,1\}} = (1-\Lambda_{m,1})(T_{\mathrm{A}} - T_{\mathrm{B}}) + T_{\mathrm{B}} \\ T_{\mathrm{b},\{m,2\}} = (1-\Lambda_{m,1}-\Lambda_{m,2})(T_{\mathrm{A}} - T_{\mathrm{B}}) + T_{\mathrm{B}} \\ T_{\mathrm{b},\{m,3\}} = (1-\Lambda_{m,1}-\Lambda_{m,2}-\Lambda_{m,3})(T_{\mathrm{A}} - T_{\mathrm{B}}) + T_{\mathrm{B}} \\ \cdots\cdots\cdots\cdots\cdots\cdots\cdots\cdots\cdots\cdots\cdots\cdots\cdots\cdots\cdots \\ T_{\mathrm{b},\{m,n\}} = (1-\Lambda_{m,1}-\Lambda_{m,2}-\cdots-\Lambda_{m,n})(T_{\mathrm{A}} - T_{\mathrm{B}}) + T_{\mathrm{B}} \end{cases}. \tag{S4}$$

From Eq. (S4), we deduce

$$T_{\mathrm{b},\{m,j\}} = \left(1 - \sum_{j^*=1}^{j} \Lambda_{\{m,j^*\}}\right)(T_{\mathrm{A}} - T_{\mathrm{B}}) + T_{\mathrm{B}} \tag{S5a}$$

$$= T_{\mathrm{A}} - (T_{\mathrm{A}} - T_{\mathrm{B}}) \sum_{j^*=1}^{j} \Lambda_{\{m,j^*\}}. \tag{S5b}$$

Following the equivalent thermal resistance method, we have

$$T_{\mathrm{a},\{m,j^*\}} - T_{\mathrm{b},\{m,j^*\}} = q_m \sum_{\epsilon=\mathrm{l,c,r}} \frac{d_{\epsilon,\{m,j^*\}}}{\kappa_{\epsilon,\{m,j^*\}}}. \tag{S6}$$

Combining Eq. (S5a), Eq. (S3), Eq. (S5b), and Eq. (S6), we finally obtain Eq. (5b).

In addition, it should be mentioned that another relationship can directly observe Eq. (S5b)

$$\begin{cases} T_{\mathrm{b},\{m,1\}} = T_\mathrm{A} - \Lambda_{m,1}\left(T_\mathrm{A} - T_\mathrm{B}\right) \\ T_{\mathrm{b},\{m,2\}} = T_\mathrm{A} - \left(\Lambda_{m,1} + \Lambda_{m,2}\right)\left(T_\mathrm{A} - T_\mathrm{B}\right) \\ T_{\mathrm{b},\{m,3\}} = T_\mathrm{A} - \left(\Lambda_{m,1} + \Lambda_{m,2} + \Lambda_{m,3}\right)\left(T_\mathrm{A} - T_\mathrm{B}\right) \\ \cdots\cdots\cdots\cdots\cdots\cdots\cdots\cdots\cdots\cdots\cdots \\ T_{\mathrm{b},\{m,n\}} = T_\mathrm{A} - \left(\Lambda_{m,1} + \Lambda_{m,2} + \Lambda_{m,3} + \cdots + \Lambda_{m,n}\right)\left(T_\mathrm{A} - T_\mathrm{B}\right) \end{cases}. \tag{S7}$$

Eq. (S5a) and Eq. (S5b) are two equivalent expressions for $T_{\mathrm{b},\{m,j\}}$, which can be applied to demand.

1.3 Derivation for $\left|\nabla T_{\mathrm{c},\{m,j\}}\right| \to 0$

From Eq. (6),

$$\left|\nabla T_{\mathrm{c},\{m,j\}}\right| = \left|\frac{\mathrm{d}T_{\mathrm{c},\{m,j\}}}{\mathrm{d}x}\right| = \frac{T_\mathrm{A} - T_\mathrm{B}}{\left(\sum_{j\#=1}^{n}\sum_{\epsilon=\mathrm{l,c,r}} d_{\epsilon,\{m,j\#\}}/\kappa_{\epsilon,\{m,j\#\}}\right)\kappa_{\mathrm{c},\{m,j\}}}. \tag{S8}$$

Expanding the summation term in Eq. (S8) and arranging, we have

$$\left|\nabla T_{\mathrm{c},\{m,j\}}\right| = \frac{T_\mathrm{A} - T_\mathrm{B}}{\sum_{j\#=1}^{n}\sum_{\epsilon=\mathrm{l,c,r}}\left(d_{\mathrm{l},\{m,j\#\}}\frac{\kappa_{\mathrm{c},\{m,j\}}}{\kappa_{\mathrm{l},\{m,j\#\}}} + d_{\mathrm{c},\{m,j\#\}}\frac{\kappa_{\mathrm{c},\{m,j\}}}{\kappa_{\mathrm{c},\{m,j\#\}}} + d_{\mathrm{r},\{m,j\#\}}\frac{\kappa_{\mathrm{c},\{m,j\}}}{\kappa_{\mathrm{r},\{m,j\#\}}}\right)}. \tag{S9}$$

When $\kappa_{\mathrm{l},\{m,j\#\}} << \kappa_{\mathrm{c},\{m,j\}}$ or $\kappa_{\mathrm{r},\{m,j\#\}} << \kappa_{\mathrm{c},\{m,j\}}$,

$$\sum_{j\#=1}^{n}\sum_{\epsilon=\mathrm{l,c,r}}\left(d_{\mathrm{l},\{m,j\#\}}\frac{\kappa_{\mathrm{c},\{m,j\}}}{\kappa_{\mathrm{l},\{m,j\#\}}} + d_{\mathrm{c},\{m,j\#\}}\frac{\kappa_{\mathrm{c},\{m,j\}}}{\kappa_{\mathrm{c},\{m,j\#\}}} + d_{\mathrm{r},\{m,j\#\}}\frac{\kappa_{\mathrm{c},\{m,j\}}}{\kappa_{\mathrm{r},\{m,j\#\}}}\right) \to \infty, \tag{S10}$$

so $\left|\nabla T_{\mathrm{c},\{m,j\}}\right| \to 0$. It means that the temperature in $\mathrm{C}_{\{m,j\}}$ is uniform. The condition that $\kappa_{\epsilon,\{m,j\#\}} << \kappa_{\mathrm{c},\{m,j\}}$ $\left(\kappa_{\epsilon,\{m,j^*\}} << \kappa_{\mathrm{c},\{m,j\#\}},\ \kappa_{\epsilon,\{m,j\}} << \kappa_{\mathrm{c},\{m,j\}},\ \epsilon = \mathrm{l,r}\right)$ is termed as a temperature-uniform condition.

1.4 Derivation for Eq. (1)

From Eq. (6)

$$\begin{aligned}T_{\mathrm{c},\{m,j\}} &= \frac{T_\mathrm{A}-T_\mathrm{B}}{\sum_{j^\#=1}^{n}\left(d_{\mathrm{l},\{m,j^\#\}}\frac{\kappa_{\mathrm{r},\{m,j\}}}{\kappa_{\mathrm{l},\{m,j^\#\}}}+d_{\mathrm{c},\{m,j^\#\}}\frac{\kappa_{\mathrm{r},\{m,j\}}}{\kappa_{\mathrm{c},\{m,j^\#\}}}+d_{\mathrm{r},\{m,j^\#\}}\frac{\kappa_{\mathrm{r},\{m,j\}}}{\kappa_{\mathrm{r},\{m,j^\#\}}}\right)}d_{\mathrm{r},\{m,j\}}\\
&+\frac{T_\mathrm{A}-T_\mathrm{B}}{\sum_{j^\#=1}^{n}\left(d_{\mathrm{l},\{m,j^\#\}}\frac{\kappa_{\mathrm{c},\{m,j\}}}{\kappa_{\mathrm{l},\{m,j^\#\}}}+d_{\mathrm{c},\{m,j^\#\}}\frac{\kappa_{\mathrm{c},\{m,j\}}}{\kappa_{\mathrm{c},\{m,j^\#\}}}+d_{\mathrm{r},\{m,j^\#\}}\frac{\kappa_{\mathrm{c},\{m,j\}}}{\kappa_{\mathrm{r},\{m,j^\#\}}}\right)}\left(\frac{d_{\mathrm{c},\{m,j\}}}{2}-x_{\{m,j\}}\right)\\
&+T_\mathrm{A}-(T_\mathrm{A}-T_\mathrm{B})\sum_{j^*=1}^{j}\frac{d_{\mathrm{l},\{m,j^*\}}\frac{\kappa_{\mathrm{c},\{m,j^\#\}}}{\kappa_{\mathrm{l},\{m,j^*\}}}+d_{\mathrm{c},\{m,j^*\}}\frac{\kappa_{\mathrm{c},\{m,j^\#\}}}{\kappa_{\mathrm{c},\{m,j^*\}}}+d_{\mathrm{r},\{m,j^*\}}\frac{\kappa_{\mathrm{c},\{m,j^\#\}}}{\kappa_{\mathrm{r},\{m,j^*\}}}}{\sum_{j^\#=1}^{n}\left(d_{\mathrm{l},\{m,j^\#\}}\frac{\kappa_{\mathrm{c},\{m,j^\#\}}}{\kappa_{\mathrm{l},\{m,j^\#\}}}+d_{\mathrm{c},\{m,j^\#\}}+d_{\mathrm{r},\{m,j^\#\}}\frac{\kappa_{\mathrm{c},\{m,j^\#\}}}{\kappa_{\mathrm{r},\{m,j^\#\}}}\right)}.\end{aligned}\tag{S11}$$

For Eq. (S11), under the temperature-uniform condition, the denominator of the first term in the right side

$$\begin{aligned}&\sum_{j^\#=1}^{n}\left(d_{\mathrm{l},\{m,j^\#\}}\frac{\kappa_{\mathrm{r},\{m,j\}}}{\kappa_{\mathrm{l},\{m,j^\#\}}}+d_{\mathrm{c},\{m,j^\#\}}\frac{\kappa_{\mathrm{r},\{m,j\}}}{\kappa_{\mathrm{c},\{m,j^\#\}}}+d_{\mathrm{r},\{m,j^\#\}}\frac{\kappa_{\mathrm{r},\{m,j\}}}{\kappa_{\mathrm{r},\{m,j^\#\}}}\right)\\
&=\sum_{j^\#=1}^{n}\sum_{\epsilon=\mathrm{l},\mathrm{r}}\frac{d_{\epsilon,\{m,j^\#\}}}{\kappa_{\epsilon,\{m,j^\#\}}}\kappa_{\mathrm{r},\{m,j\}}.\end{aligned}\tag{S12}$$

The denominator of the second term

$$\sum_{j^\#=1}^{n}\left(d_{\mathrm{l},\{m,j^\#\}}\frac{\kappa_{\mathrm{c},\{m,j\}}}{\kappa_{\mathrm{l},\{m,j^\#\}}}+d_{\mathrm{c},\{m,j^\#\}}\frac{\kappa_{\mathrm{c},\{m,j\}}}{\kappa_{\mathrm{c},\{m,j^\#\}}}+d_{\mathrm{r},\{m,j^\#\}}\frac{\kappa_{\mathrm{c},\{m,j\}}}{\kappa_{\mathrm{r},\{m,j^\#\}}}\right)\to\infty.\tag{S13}$$

Observing the summation in the fourth term, for the numerator, $d_{\mathrm{l},\{m,j^*\}}\frac{\kappa_{\mathrm{c},\{m,j^\#\}}}{\kappa_{\mathrm{l},\{m,j^*\}}}+d_{\mathrm{r},\{m,j^*\}}\frac{\kappa_{\mathrm{c},\{m,j^\#\}}}{\kappa_{\mathrm{r},\{m,j^*\}}}\to\infty$, so $d_{\mathrm{c},\{m,j^*\}}\frac{\kappa_{\mathrm{c},\{m,j^\#\}}}{\kappa_{\mathrm{c},\{m,j^*\}}}$ are relatively small amounts; for the denominator, $d_{\mathrm{l},\{m,j^\#\}}\frac{\kappa_{\mathrm{c},\{m,j^\#\}}}{\kappa_{\mathrm{l},\{m,j^\#\}}}+d_{\mathrm{r},\{m,j^\#\}}\frac{\kappa_{\mathrm{c},\{m,j^\#\}}}{\kappa_{\mathrm{r},\{m,j^\#\}}}\to\infty$, so $d_{\mathrm{c},\{m,j^\#\}}$ are relatively small amounts. Then, the summation

$$\begin{aligned}&\sum_{j^*=1}^{j}\frac{d_{\mathrm{l},\{m,j^*\}}\frac{\kappa_{\mathrm{c},\{m,j^\#\}}}{\kappa_{\mathrm{l},\{m,j^*\}}}+d_{\mathrm{c},\{m,j^*\}}\frac{\kappa_{\mathrm{c},\{m,j^\#\}}}{\kappa_{\mathrm{c},\{m,j^*\}}}+d_{\mathrm{r},\{m,j^*\}}\frac{\kappa_{\mathrm{c},\{m,j^\#\}}}{\kappa_{\mathrm{r},\{m,j^*\}}}}{\sum_{j^\#=1}^{n}\left(d_{\mathrm{l},\{m,j^\#\}}\frac{\kappa_{\mathrm{c},\{m,j^\#\}}}{\kappa_{\mathrm{l},\{m,j^\#\}}}+d_{\mathrm{c},\{m,j^\#\}}+d_{\mathrm{r},\{m,j^\#\}}\frac{\kappa_{\mathrm{c},\{m,j^\#\}}}{\kappa_{\mathrm{r},\{m,j^\#\}}}\right)}\\
&=\sum_{j^*=1}^{j}\frac{d_{\mathrm{l},\{m,j^*\}}\frac{\kappa_{\mathrm{c},\{m,j^\#\}}}{\kappa_{\mathrm{l},\{m,j^*\}}}+d_{\mathrm{r},\{m,j^*\}}\frac{\kappa_{\mathrm{c},\{m,j^\#\}}}{\kappa_{\mathrm{r},\{m,j^*\}}}}{\sum_{j^\#=1}^{n}\left(d_{\mathrm{l},\{m,j^\#\}}\frac{\kappa_{\mathrm{c},\{m,j^\#\}}}{\kappa_{\mathrm{l},\{m,j^\#\}}}+d_{\mathrm{r},\{m,j^\#\}}\frac{\kappa_{\mathrm{c},\{m,j^\#\}}}{\kappa_{\mathrm{r},\{m,j^\#\}}}\right)}\\
&=\sum_{j^*=1}^{j}\frac{\sum_{\epsilon=\mathrm{l},\mathrm{r}}d_{\epsilon,\{m,j^*\}}/\kappa_{\epsilon,\{m,j^*\}}}{\sum_{j^\#=1}^{n}\sum_{\epsilon=\mathrm{l},\mathrm{r}}d_{\epsilon,\{m,j^\#\}}/\kappa_{\epsilon,\{m,j^\#\}}}\end{aligned}.\tag{S14}$$

Using Eqs. (S12)-(S14) to simplify Eq. (S11), we finally get Eq. (1).

1.5 Another method: normalized temperature expression

In this section, we express $T_{\mathrm{c},\{m,j\}}$ in another form. We normalize $T_{\mathrm{c},\{m,j\}}$

$$\theta_{\mathrm{c},\{m,j\}} = \frac{T_{\mathrm{c},\{m,j\}} - T_{\mathrm{b},\{m,j\}}}{T_{\mathrm{a},\{m,j\}} - T_{\mathrm{b},\{m,j\}}}, \tag{S15}$$

and

$$\Psi_{\mathrm{c},\{m,j\}} = \frac{T_{\mathrm{c},\{m,j\}} - T_{\mathrm{B}}}{T_{\mathrm{A}} - T_{\mathrm{B}}}. \tag{S16}$$

from the perspective of the local system and the global system, respectively. Multiplying Eq. (S15) and Eq. (S3)

$$\theta_{\mathrm{c},\{m,j\}} \Lambda_{\{m,j\}} = \frac{T_{\mathrm{c},\{m,j\}} - T_{\mathrm{b},\{m,j\}}}{T_{\mathrm{A}} - T_{\mathrm{B}}}. \tag{S17}$$

Replacing the $T_{\mathrm{b},\{m,j\}}$ in Eq. (S17) by Eq. (S5a)

$$\begin{aligned} \theta_{\mathrm{c},\{m,j\}} \Lambda_{\{m,j\}} &= \frac{T_{\mathrm{c},\{m,j\}} - \left(1 - \sum_{j^*=1}^{j} \Lambda_{\{m,j^*\}}\right)(T_{\mathrm{A}} - T_{\mathrm{B}}) - T_{\mathrm{B}}}{T_{\mathrm{A}} - T_{\mathrm{B}}} \\ &= \frac{T_{\mathrm{c},\{m,j\}} - T_{\mathrm{B}}}{T_{\mathrm{A}} - T_{\mathrm{B}}} - 1 + \sum_{j^*=1}^{j} \Lambda_{\{m,j^*\}}. \end{aligned} \tag{S18}$$

Substituting Eq. (S16) into Eq. (S18) and arranging, we finally get

$$\Psi_{\mathrm{c},\{m,j\}} = \theta_{\mathrm{c},\{m,j\}} \Lambda_{\{m,j\}} + 1 - \sum_{j^*=1}^{j} \Lambda_{\{m,j^*\}}. \tag{S19}$$

Since $\Lambda_{\{m,j\}}$ is the temperature range ratio mentioned above [Eq. (S3)], we can make a presetting in the designing process. For example, we can set $\Lambda_{\{m,j\}} = 1/n$. Then, Eq. (S19) can be rewritten as

$$\Psi_{\mathrm{c},\{m,j\}} = \frac{\theta_{\mathrm{c},\{m,j\}}}{n} - \frac{j}{n} + 1. \tag{S20}$$

Considering Eq. (S15) and the equivalent thermal resistance method, we have

$$\begin{aligned} \theta_{\mathrm{c},\{m,j\}} &= \left(\frac{d_{\mathrm{r},\{m,j\}}}{\kappa_{\mathrm{r},\{m,j\}}} + \frac{d_{\mathrm{c},\{m,j\}}/2 - x_{\{m,j\}}}{\kappa_{\mathrm{c},\{m,j\}}}\right) \Big/ \left(\sum_{\epsilon=\mathrm{l,c,r}} \frac{d_{\epsilon,\{m,j\}}}{\kappa_{\epsilon,\{m,j\}}}\right) \\ &= \frac{d_{\mathrm{r},\{m,j\}}}{d_{\mathrm{l},\{m,j\}} \frac{\kappa_{\mathrm{r},\{m,j\}}}{\kappa_{\mathrm{l},\{m,j\}}} + d_{\mathrm{c},\{m,j\}} \frac{\kappa_{\mathrm{r},\{m,j\}}}{\kappa_{\mathrm{c},\{m,j\}}} + d_{\mathrm{r},\{m,j\}}} \\ &+ \frac{d_{\mathrm{c},\{m,j\}}/2 - x_{\{m,j\}}}{d_{\mathrm{l},\{m,j\}} \frac{\kappa_{\mathrm{c},\{m,j\}}}{\kappa_{\mathrm{l},\{m,j\}}} + d_{\mathrm{c},\{m,j\}} + d_{\mathrm{r},\{m,j\}} \frac{\kappa_{\mathrm{c},\{m,j\}}}{\kappa_{\mathrm{r},\{m,j\}}}}. \end{aligned} \tag{S21}$$

According to the temperature-uniform condition, Eq. (S21) can be rewritten as

$$\theta_{\mathrm{c},\{m,j\}} = \frac{d_{\mathrm{r},\{m,j\}}}{d_{\mathrm{l},\{m,j\}} \frac{\kappa_{\mathrm{r},\{m,j\}}}{\kappa_{\mathrm{l},\{m,j\}}} + d_{\mathrm{r},\{m,j\}}} = \frac{1}{\frac{d_{\mathrm{l},\{m,j\}}}{d_{\mathrm{r},\{m,j\}}} \frac{\kappa_{\mathrm{r},\{m,j\}}}{\kappa_{\mathrm{l},\{m,j\}}} + 1}. \tag{S22}$$

Substituting Eq. (S22) into Eq. (S20)

$$\Psi_{\mathrm{c},\{m,j\}} = \frac{1}{n}\left[1\bigg/\left(\frac{d_{\mathrm{l},\{m,j\}}}{d_{\mathrm{r},\{m,j\}}}\frac{\kappa_{\mathrm{r},\{m,j\}}}{\kappa_{\mathrm{l},\{m,j\}}}+1\right)-j\right]+1. \tag{S23}$$

Eq. (S23) suggests that we possess $4n$ adjustable parameters $\left(\kappa_{\mathrm{l},\{m,j\}}, \kappa_{\mathrm{r},\{m,j\}}, d_{\mathrm{l},\{m,j\}}, d_{\mathrm{r},\{m,j\}}\right)$ to control n required $\Psi_{\mathrm{c},\{m,j\}}$ $(j = \{1, 2, 3\cdots n\})$. Thus, we have an ample number of adjustable parameters. In real-world scenarios, this allows for the flexible design of the multi-temperature control system. A comprehensive calculation method for solving Eq. (S23) is provided in Supplementary Note 5.3.

2 Simplification for the (effective) thermal conductivity of the temperature control zone

As detailed in the manuscript, the temperature control zone $\mathrm{C}_{\{i,j\}}$ comprises both a storage space and a package structure. The storage space caters to goods, and the package structure safeguards these goods. Supplementary Figs. 2, 3, and 5-13 depict the intricate relationships between the goods and the package structure. Consequently, the thermal conductivity of the temperature control zone $\kappa_{\mathrm{c},\{i,j\}}$ is better described as the effective thermal conductivity $\kappa_{\mathrm{c},\{i,j\},\mathrm{eff}}$. Following the effective medium theory, $\kappa_{\mathrm{c},\{i,j\},\mathrm{eff}}$ correlates with the thermal conductivities of the goods $\kappa_{\mathrm{o},\{i,j\}}$ (assuming the thermal conductivities of all goods in $\mathrm{C}_{\{i,j\}}$ are uniform), the thermal conductivity of the package structure $\kappa_{\mathrm{pac},\{i,j\}}$, the area fraction of the goods $f_{\mathrm{o},\{i,j\}}$, and the area fraction of the package structure $f_{\mathrm{pac},\{i,j\}}$. For ease of understanding in the "Principle of AMTC," we posited $\kappa_{\mathrm{c},\{i,j\}} = \kappa_{\mathrm{o},\{i,j\}} = \kappa_{\mathrm{pac},\{i,j\}} = \kappa_{\mathrm{c},\{i,j\},\mathrm{eff}}$. The impact of $\kappa_{\mathrm{o},\{i,j\}}$ on the system's multi-temperature control performance is elaborated in Supplementary Note 9.1. The methodology to calculate $\kappa_{\mathrm{c},\{i,j\},\mathrm{eff}}$ is presented in Supplementary Note 10.

3 Symbols for goods in each temperature control zone

As previously discussed, we used $T_{\mathrm{c},\{i,j\}}$ and $T_{\mathrm{o},\{i,j\}}$ to represent the temperature of the temperature control zone $\mathrm{C}_{\{i,j\}}$ and the goods stored in $\mathrm{C}_{\{i,j\}}$, respectively. This notation aids in discussions and facilitates calculations of the multi-temperature control system's thermal and structural parameters. For subsequent discussions, these can be succinctly represented as T_γ where γ serves as an annotation for both the temperature control zone γ and its stored goods. In this context, γ={①, ②, . . . , ⓩ}, with z indicating the count of temperature control zones. For instance, in the "Multi-temperature maintenance container based on AMTC," we designed a multi-temperature control system spanning 3×3 zones. Here, $T_{\mathrm{o},\{i,j\},\mathrm{re}}$ denotes the multi-temperature requirements for goods within $\mathrm{C}_{\{m,j\}}$. Computations can rigorously adhere to the symbolic conventions of our theory, and the outcomes can be neatly encapsulated in matrix notation. However, for performance illustrations derived from experiments (or finite element simulations), the former symbol is superfluous, prompting us to adopt T_γ for clarity. These two symbols are wholly interchangeable. In the aforementioned scenario, their interrelationships

are encapsulated as:

$$\left[T_{\mathrm{o},\{i,j\},\mathrm{re}}\right]_{3\times3}=\begin{bmatrix}T_{\mathrm{o},\{1,1\}} & T_{\mathrm{o},\{1,2\}} & T_{\mathrm{o},\{1,3\}}\\ T_{\mathrm{o},\{2,1\}} & T_{\mathrm{o},\{2,2\}} & T_{\mathrm{o},\{2,3\}}\\ T_{\mathrm{o},\{3,1\}} & T_{\mathrm{o},\{3,2\}} & T_{\mathrm{o},\{3,3\}}\end{bmatrix}=\left[T_{\gamma,\mathrm{re}}\right]_{3\times3}=\begin{bmatrix}T_{①} & T_{④} & T_{⑦}\\ T_{②} & T_{⑤} & T_{⑧}\\ T_{③} & T_{⑥} & T_{⑨}\end{bmatrix}. \tag{S24}$$

In light of this, we can employ symbols like ①, ②, $\cdots$, ⑨ to label each individual temperature control zone and its corresponding stored goods.

4 Design methods of the 2-D multi-temperature control system based on AMTC

To elucidate the design methods, we selected a 2-D multi-temperature control system as depicted in Supplementary Fig. 5a. The system's geometry and structural parameters are detailed in Supplementary Fig. 2a1, b1. For clarity and ease of comprehension, we employ a direct temperature notation rather than a normalized temperature expression. The temperature of the heat and cold sources are set as $T_{\mathrm{A}}=400\ \mathrm{K}$ and $T_{\mathrm{B}}=300\ \mathrm{K}$, respectively. We set $\kappa_{\mathrm{c},\{i,j\}}=10\ \mathrm{W\ m^{-1}\ K^{-1}}$ and

$$\left[T_{\mathrm{o},\{i,j\},\mathrm{re}}\right]_{3\times3}=\begin{bmatrix}390 & 360 & 330\\ 380 & 350 & 320\\ 370 & 340 & 310\end{bmatrix}\ \mathrm{K}. \tag{S25}$$

Using Eq. (1), we obtained

$$\begin{cases}\left[\kappa_{\mathrm{l},\{i,j\}}\right]_{3\times3}=\begin{bmatrix}0.308 & 0.471 & 1.000\\ 0.100 & 0.120 & 0.151\\ 0.033 & 0.037 & 0.043\end{bmatrix}\ \mathrm{W\ m^{-1}\ K^{-1}}\\ \left[\kappa_{\mathrm{r},\{i,j\}}\right]_{3\times3}=\begin{bmatrix}0.129 & 0.113 & 0.100\\ 0.151 & 0.120 & 0.100\\ 0.308 & 0.151 & 0.100\end{bmatrix}\ \mathrm{W\ m^{-1}\ K^{-1}}\end{cases}, \tag{S26}$$

(a comprehensive calculation methodology is available in Supplementary Note 5, approach 5). Further assuming

$$\begin{cases}\kappa_{\mathrm{o},\{i,j\}}=\kappa_{\mathrm{pac},\{i,j\}}=\kappa_{\mathrm{c},\{i,j\}}=10\ \mathrm{W\ m^{-1}\ K^{-1}}\\ \kappa_{\mathrm{s}}=0.000001\ \mathrm{W\ m^{-1}\ K^{-1}}\ (\text{thermal conductivity of zone S})\end{cases}, \tag{S27}$$

and by specifying the structural parameters of the goods within $\mathrm{C}_{\{i,j\}}$ (Supplementary Fig. 2b1), we finally obtained all the requisite parameters of the multi-temperature control system for steady-state simulations.

5 Calculating the thermal/structural parameters of the multi-temperature control system.

We choose the 2-D multi-temperature control system in Supplementary Note 4 as a case for calculation.

5.1 Approach 1

According to Eq. (1) and Eq. (S25), for $i = 1$, we set $m = 1$

$$\begin{cases} 390 = T_\mathrm{A} - \dfrac{T_\mathrm{A} - T_\mathrm{B}}{\sum_{j\#=1}^{3} \sum_{\epsilon=\mathrm{l,r}} d_{\epsilon,\{1,j\#\}} / \kappa_{\epsilon,\{1,j\#\}}} \left(-\dfrac{d_{\mathrm{r},\{1,1\}}}{\kappa_{\mathrm{r},\{1,1\}}} + \sum_{j^*=1}^{1} \sum_{\epsilon=\mathrm{l,r}} \dfrac{d_{\epsilon,\{1,j^*\}}}{\kappa_{\epsilon,\{1,j^*\}}} \right) \\ 360 = T_\mathrm{A} - \dfrac{T_\mathrm{A} - T_\mathrm{B}}{\sum_{j\#=1}^{3} \sum_{\epsilon=\mathrm{l,r}} d_{\epsilon,\{1,j\#\}} / \kappa_{\epsilon,\{1,j\#\}}} \left(-\dfrac{d_{\mathrm{r},\{1,2\}}}{\kappa_{\mathrm{r},\{1,2\}}} + \sum_{j^*=1}^{2} \sum_{\epsilon=\mathrm{l,r}} \dfrac{d_{\epsilon,\{1,j^*\}}}{\kappa_{\epsilon,\{1,j^*\}}} \right) \\ 330 = T_\mathrm{A} - \dfrac{T_\mathrm{A} - T_\mathrm{B}}{\sum_{j\#=1}^{3} \sum_{\epsilon=\mathrm{l,r}} d_{\epsilon,\{1,j\#\}} / \kappa_{\epsilon,\{1,j\#\}}} \left(-\dfrac{d_{\mathrm{r},\{1,3\}}}{\kappa_{\mathrm{r},\{1,3\}}} + \sum_{j^*=1}^{3} \sum_{\epsilon=\mathrm{l,r}} \dfrac{d_{\epsilon,\{1,j^*\}}}{\kappa_{\epsilon,\{1,j^*\}}} \right) \end{cases}. \tag{S28}$$

For $i = 2$, we set $m = 2$

$$\begin{cases} 380 = T_\mathrm{A} - \dfrac{T_\mathrm{A} - T_\mathrm{B}}{\sum_{j\#=1}^{3} \sum_{\epsilon=\mathrm{l,r}} d_{\epsilon,\{2,j\#\}} / \kappa_{\epsilon,\{2,j\#\}}} \left(-\dfrac{d_{\mathrm{r},\{2,1\}}}{\kappa_{\mathrm{r},\{2,1\}}} + \sum_{j^*=1}^{1} \sum_{\epsilon=\mathrm{l,r}} \dfrac{d_{\epsilon,\{2,j^*\}}}{\kappa_{\epsilon,\{2,j^*\}}} \right) \\ 350 = T_\mathrm{A} - \dfrac{T_\mathrm{A} - T_\mathrm{B}}{\sum_{j\#=1}^{3} \sum_{\epsilon=\mathrm{l,r}} d_{\epsilon,\{2,j\#\}} / \kappa_{\epsilon,\{2,j\#\}}} \left(-\dfrac{d_{\mathrm{r},\{2,2\}}}{\kappa_{\mathrm{r},\{2,2\}}} + \sum_{j^*=1}^{2} \sum_{\epsilon=\mathrm{l,r}} \dfrac{d_{\epsilon,\{2,j^*\}}}{\kappa_{\epsilon,\{2,j^*\}}} \right) \\ 320 = T_\mathrm{A} - \dfrac{T_\mathrm{A} - T_\mathrm{B}}{\sum_{j\#=1}^{3} \sum_{\epsilon=\mathrm{l,r}} d_{\epsilon,\{2,j\#\}} / \kappa_{\epsilon,\{2,j\#\}}} \left(-\dfrac{d_{\mathrm{r},\{2,3\}}}{\kappa_{\mathrm{r},\{2,3\}}} + \sum_{j^*=1}^{3} \sum_{\epsilon=\mathrm{l,r}} \dfrac{d_{\epsilon,\{2,j^*\}}}{\kappa_{\epsilon,\{2,j^*\}}} \right) \end{cases}. \tag{S29}$$

For $i = 3$, we set $m = 3$

$$\begin{cases} 370 = T_\mathrm{A} - \dfrac{T_\mathrm{A} - T_\mathrm{B}}{\sum_{j\#=1}^{3} \sum_{\epsilon=\mathrm{l,r}} d_{\epsilon,\{3,j\#\}} / \kappa_{\epsilon,\{3,j\#\}}} \left(-\dfrac{d_{\mathrm{r},\{3,1\}}}{\kappa_{\mathrm{r},\{3,1\}}} + \sum_{j^*=1}^{1} \sum_{\epsilon=\mathrm{l,r}} \dfrac{d_{\epsilon,\{3,j^*\}}}{\kappa_{\epsilon,\{3,j^*\}}} \right) \\ 340 = T_\mathrm{A} - \dfrac{T_\mathrm{A} - T_\mathrm{B}}{\sum_{j\#=1}^{3} \sum_{\epsilon=\mathrm{l,r}} d_{\epsilon,\{3,j\#\}} / \kappa_{\epsilon,\{3,j\#\}}} \left(-\dfrac{d_{\mathrm{r},\{3,2\}}}{\kappa_{\mathrm{r},\{3,2\}}} + \sum_{j^*=1}^{2} \sum_{\epsilon=\mathrm{l,r}} \dfrac{d_{\epsilon,\{3,j^*\}}}{\kappa_{\epsilon,\{3,j^*\}}} \right) \\ 310 = T_\mathrm{A} - \dfrac{T_\mathrm{A} - T_\mathrm{B}}{\sum_{j\#=1}^{3} \sum_{\epsilon=\mathrm{l,r}} d_{\epsilon,\{3,j\#\}} / \kappa_{\epsilon,\{3,j\#\}}} \left(-\dfrac{d_{\mathrm{r},\{3,3\}}}{\kappa_{\mathrm{r},\{3,3\}}} + \sum_{j^*=1}^{3} \sum_{\epsilon=\mathrm{l,r}} \dfrac{d_{\epsilon,\{3,j^*\}}}{\kappa_{\epsilon,\{3,j^*\}}} \right) \end{cases}. \tag{S30}$$

From the calculation case (Supplementary Note 4), $T_\mathrm{A} = 400$ K, $T_\mathrm{B} = 300$ K, $\left[d_{\mathrm{l},\{1,j\}}\right]_{1\times3} = \left[d_{\mathrm{r},\{1,j\}}\right]_{1\times3} = [20]_{1\times3}$ mm. Setting $\kappa_{\mathrm{l},\{1,1\}} = \kappa_{\mathrm{l},\{1,2\}} = \kappa_{\mathrm{l},\{1,3\}} = 0.050\ \mathrm{W\ m^{-1}\ K^{-1}}$, Eq. (S28) can be solved. We obtained $\kappa_{\mathrm{r},\{1,1\}} = 0.025\ \mathrm{W\ m^{-1}\ K^{-1}}$, $\kappa_{\mathrm{r},\{1,2\}} = 0.025\ \mathrm{W\ m^{-1}\ K^{-1}}$,

and $\kappa_{\mathrm{r},\{1,3\}} = 0.017$ W m^{-1} K^{-1}. Setting $\kappa_{\mathrm{l},\{2,1\}} = \kappa_{\mathrm{l},\{2,2\}} = \kappa_{\mathrm{l},\{2,3\}} = 0.050$ W m^{-1} K^{-1}, Eq. (S29) can be solved. We obtained $\kappa_{\mathrm{r},\{2,1\}} = 0.100$ W m^{-1} K^{-1}, $\kappa_{\mathrm{r},\{2,2\}} = 0.100$ W m^{-1} K^{-1}, and $\kappa_{\mathrm{r},\{2,3\}} = 0.050$ W m^{-1} K^{-1}. Setting $\kappa_{\mathrm{r},\{3,1\}} = \kappa_{\mathrm{r},\{3,2\}} = \kappa_{\mathrm{r},\{3,3\}} = 0.050$ W m^{-1} K^{-1}, Eq. (S30) can be solved. We obtained $\kappa_{\mathrm{l},\{3,1\}} = 0.017$ W m^{-1} K^{-1}, $\kappa_{\mathrm{l},\{3,2\}} = 0.025$ W m^{-1} K^{-1}, and $\kappa_{\mathrm{l},\{3,3\}} = 0.025$ W m^{-1} K^{-1}. Arranging the above results, we finally got

$$\left[\kappa_{\mathrm{l},\{i,j\}}\right]_{3\times 3} = \begin{bmatrix} 0.050 & 0.050 & 0.050 \\ 0.050 & 0.050 & 0.050 \\ 0.017 & 0.025 & 0.025 \end{bmatrix} \text{ W m}^{-1}\text{ K}^{-1}, \tag{S31}$$

and

$$\left[\kappa_{\mathrm{r},\{i,j\}}\right]_{3\times 3} = \begin{bmatrix} 0.025 & 0.025 & 0.017 \\ 0.100 & 0.100 & 0.050 \\ 0.050 & 0.050 & 0.050 \end{bmatrix} \text{ W m}^{-1}\text{ K}^{-1}. \tag{S32}$$

5.2 Approach 2

Following Eq. (S3), the temperature-uniform condition, and the equivalent thermal resistance method

$$\Lambda_{\{m,j\}} = \frac{\sum_{\epsilon=\mathrm{l,r}} d_{\epsilon,\{m,j\}}/\kappa_{\epsilon,\{m,j\}}}{\sum_{j\#=1}^{n}\sum_{\epsilon=\mathrm{l,r}} d_{\epsilon,\{m,j\#\}}/\kappa_{\epsilon,\{m,j\#\}}}. \tag{S33}$$

For $i = 1$, we set $m = 1$, according to Eq. (S16), Eq. (S19), Eq. (S22), Eq. (S25), and Eq. (S33), we have

$$\begin{cases} 0.9 = \theta_{\mathrm{c},\{1,1\}}\Lambda_{\{1,1\}} + 1 - \sum\limits_{j^*=1}^{1}\Lambda_{\{1,j^*\}} \\ 0.6 = \theta_{\mathrm{c},\{1,2\}}\Lambda_{\{1,2\}} + 1 - \sum\limits_{j^*=1}^{2}\Lambda_{\{1,j^*\}} \\ 0.3 = \theta_{\mathrm{c},\{1,3\}}\Lambda_{\{1,3\}} + 1 - \sum\limits_{j^*=1}^{3}\Lambda_{\{1,j^*\}} \\ \theta_{\mathrm{c},\{1,1\}} = 1\Big/\left(\dfrac{d_{\mathrm{l},\{1,1\}}}{d_{\mathrm{r},\{1,1\}}}\dfrac{\kappa_{\mathrm{r},\{1,1\}}}{\kappa_{\mathrm{l},\{1,1\}}} + 1\right) \\ \theta_{\mathrm{c},\{1,2\}} = 1\Big/\left(\dfrac{d_{\mathrm{l},\{1,2\}}}{d_{\mathrm{r},\{1,2\}}}\dfrac{\kappa_{\mathrm{r},\{1,2\}}}{\kappa_{\mathrm{l},\{1,2\}}} + 1\right) \\ \theta_{\mathrm{c},\{1,3\}} = 1\Big/\left(\dfrac{d_{\mathrm{l},\{1,3\}}}{d_{\mathrm{r},\{1,3\}}}\dfrac{\kappa_{\mathrm{r},\{1,3\}}}{\kappa_{\mathrm{l},\{1,3\}}} + 1\right) \\ \Lambda_{\{1,1\}} = \dfrac{\sum_{\epsilon=\mathrm{l,r}} d_{\epsilon,\{1,1\}}/\kappa_{\epsilon,\{1,1\}}}{\sum_{j\#=1}^{3}\sum_{\epsilon=\mathrm{l,r}} d_{\epsilon,\{1,j\#\}}/\kappa_{\epsilon,\{1,j\#\}}} \\ \Lambda_{\{1,2\}} = \dfrac{\sum_{\epsilon=\mathrm{l,r}} d_{\epsilon,\{1,2\}}/\kappa_{\epsilon,\{1,2\}}}{\sum_{j\#=1}^{3}\sum_{\epsilon=\mathrm{l,r}} d_{\epsilon,\{1,j\#\}}/\kappa_{\epsilon,\{1,j\#\}}} \\ \Lambda_{\{1,3\}} = \dfrac{\sum_{\epsilon=\mathrm{l,r}} d_{\epsilon,\{1,3\}}/\kappa_{\epsilon,\{1,3\}}}{\sum_{j\#=1}^{3}\sum_{\epsilon=\mathrm{l,r}} d_{\epsilon,\{1,j\#\}}/\kappa_{\epsilon,\{1,j\#\}}} \end{cases}. \tag{S34}$$

For $i = 2$, we set $m = 2$

$$\begin{cases} 0.8 = \theta_{\mathrm{c},\{2,1\}} \mathit{\Lambda}_{\{2,1\}} + 1 - \sum_{j^*=1}^{1} \mathit{\Lambda}_{\{2,j^*\}} \\ 0.5 = \theta_{\mathrm{c},\{2,2\}} \mathit{\Lambda}_{\{2,2\}} + 1 - \sum_{j^*=1}^{2} \mathit{\Lambda}_{\{2,j^*\}} \\ 0.2 = \theta_{\mathrm{c},\{2,3\}} \mathit{\Lambda}_{\{2,3\}} + 1 - \sum_{j^*=1}^{3} \mathit{\Lambda}_{\{2,j^*\}} \\ \theta_{\mathrm{c},\{2,1\}} = 1 \Big/ \left(\frac{d_{\mathrm{l},\{2,1\}}}{d_{\mathrm{r},\{2,1\}}} \frac{\kappa_{\mathrm{r},\{2,1\}}}{\kappa_{\mathrm{l},\{2,1\}}} + 1 \right) \\ \theta_{\mathrm{c},\{2,2\}} = 1 \Big/ \left(\frac{d_{\mathrm{l},\{2,2\}}}{d_{\mathrm{r},\{2,2\}}} \frac{\kappa_{\mathrm{r},\{2,2\}}}{\kappa_{\mathrm{l},\{2,2\}}} + 1 \right) \\ \theta_{\mathrm{c},\{2,3\}} = 1 \Big/ \left(\frac{d_{\mathrm{l},\{2,3\}}}{d_{\mathrm{r},\{2,3\}}} \frac{\kappa_{\mathrm{r},\{2,3\}}}{\kappa_{\mathrm{l},\{2,3\}}} + 1 \right) \\ \mathit{\Lambda}_{\{2,1\}} = \frac{\sum_{\epsilon=\mathrm{l,r}} d_{\epsilon,\{2,1\}} / \kappa_{\epsilon,\{2,1\}}}{\sum_{j^\#=1}^{3} \sum_{\epsilon=\mathrm{l,r}} d_{\epsilon,\{2,j^\#\}} / \kappa_{\epsilon,\{2,j^\#\}}} \\ \mathit{\Lambda}_{\{2,2\}} = \frac{\sum_{\epsilon=\mathrm{l,r}} d_{\epsilon,\{2,2\}} / \kappa_{\epsilon,\{2,2\}}}{\sum_{j^\#=1}^{3} \sum_{\epsilon=\mathrm{l,r}} d_{\epsilon,\{2,j^\#\}} / \kappa_{\epsilon,\{2,j^\#\}}} \\ \mathit{\Lambda}_{\{2,3\}} = \frac{\sum_{\epsilon=\mathrm{l,r}} d_{\epsilon,\{2,3\}} / \kappa_{\epsilon,\{2,3\}}}{\sum_{j^\#=1}^{3} \sum_{\epsilon=\mathrm{l,r}} d_{\epsilon,\{2,j^\#\}} / \kappa_{\epsilon,\{2,j^\#\}}} \end{cases} . \tag{S35}$$

For $i = 3$, we set $m = 3$

$$\begin{cases} 0.7 = \theta_{\mathrm{c},\{3,1\}} \mathit{\Lambda}_{\{3,1\}} + 1 - \sum_{j^*=1}^{1} \mathit{\Lambda}_{\{3,j^*\}} \\ 0.4 = \theta_{\mathrm{c},\{3,2\}} \mathit{\Lambda}_{\{3,2\}} + 1 - \sum_{j^*=1}^{2} \mathit{\Lambda}_{\{3,j^*\}} \\ 0.1 = \theta_{\mathrm{c},\{3,3\}} \mathit{\Lambda}_{\{3,3\}} + 1 - \sum_{j^*=1}^{3} \mathit{\Lambda}_{\{3,j^*\}} \\ \theta_{\mathrm{c},\{3,1\}} = 1 \Big/ \left(\frac{d_{\mathrm{l},\{3,1\}}}{d_{\mathrm{r},\{3,1\}}} \frac{\kappa_{\mathrm{r},\{3,1\}}}{\kappa_{\mathrm{l},\{3,1\}}} + 1 \right) \\ \theta_{\mathrm{c},\{3,2\}} = 1 \Big/ \left(\frac{d_{\mathrm{l},\{3,2\}}}{d_{\mathrm{r},\{3,2\}}} \frac{\kappa_{\mathrm{r},\{3,2\}}}{\kappa_{\mathrm{l},\{3,2\}}} + 1 \right) \\ \theta_{\mathrm{c},\{3,3\}} = 1 \Big/ \left(\frac{d_{\mathrm{l},\{3,3\}}}{d_{\mathrm{r},\{3,3\}}} \frac{\kappa_{\mathrm{r},\{3,3\}}}{\kappa_{\mathrm{l},\{3,3\}}} + 1 \right) \\ \mathit{\Lambda}_{\{3,1\}} = \frac{\sum_{\epsilon=\mathrm{l,r}} d_{\epsilon,\{3,1\}} / \kappa_{\epsilon,\{3,1\}}}{\sum_{j^\#=1}^{3} \sum_{\epsilon=\mathrm{l,r}} d_{\epsilon,\{3,j^\#\}} / \kappa_{\epsilon,\{3,j^\#\}}} \\ \mathit{\Lambda}_{\{3,2\}} = \frac{\sum_{\epsilon=\mathrm{l,r}} d_{\epsilon,\{3,2\}} / \kappa_{\epsilon,\{3,2\}}}{\sum_{j^\#=1}^{3} \sum_{\epsilon=\mathrm{l,r}} d_{\epsilon,\{3,j^\#\}} / \kappa_{\epsilon,\{3,j^\#\}}} \\ \mathit{\Lambda}_{\{3,3\}} = \frac{\sum_{\epsilon=\mathrm{l,r}} d_{\epsilon,\{3,3\}} / \kappa_{\epsilon,\{3,3\}}}{\sum_{j^\#=1}^{3} \sum_{\epsilon=\mathrm{l,r}} d_{\epsilon,\{3,j^\#\}} / \kappa_{\epsilon,\{3,j^\#\}}} \end{cases} . \tag{S36}$$

From the calculation case, we got $\left[d_{\mathrm{l},\{i,j\}}\right]_{3\times3} = \left[d_{\mathrm{r},\{i,j\}}\right]_{3\times3} = [20]_{3\times3}$ mm. Setting $\mathit{\Lambda}_{\{i,j\}} = 1/3$, we finally obtain

$$\left[\kappa_{\mathrm{l},\{i,j\}}\right]_{3\times3} = \begin{bmatrix} 0.030 & 0.045 & 0.090 \\ 0.010 & 0.012 & 0.015 \\ 0.004 & 0.004 & 0.005 \end{bmatrix} \ \mathrm{W\ m^{-1}\ K^{-1}}, \tag{S37}$$

and

$$\left[\kappa_{\mathrm{r},\{i,j\}}\right]_{3\times3} = \begin{bmatrix} 0.013 & 0.011 & 0.010 \\ 0.015 & 0.012 & 0.010 \\ 0.035 & 0.018 & 0.012 \end{bmatrix} \ \mathrm{W\ m^{-1}\ K^{-1}}. \tag{S38}$$

5.3 Approach 3

According to Eq. (S23), Eq. (S25), and Eq. (S33) $\left(\Lambda_{\{m,j\}} = 1/3\right)$, for $i = 1$, we set $m = 1$

$$\begin{cases} 0.9 = \dfrac{1}{3}\left[1\Big/\left(\dfrac{d_{\mathrm{l},\{1,1\}}}{d_{\mathrm{r},\{1,1\}}}\dfrac{\kappa_{\mathrm{r},\{1,1\}}}{\kappa_{\mathrm{l},\{1,1\}}}+1\right)-1\right]+1 \\ 0.6 = \dfrac{1}{3}\left[1\Big/\left(\dfrac{d_{\mathrm{l},\{1,2\}}}{d_{\mathrm{r},\{1,2\}}}\dfrac{\kappa_{\mathrm{r},\{1,2\}}}{\kappa_{\mathrm{l},\{1,2\}}}+1\right)-2\right]+1 \\ 0.3 = \dfrac{1}{3}\left[1\Big/\left(\dfrac{d_{\mathrm{l},\{1,3\}}}{d_{\mathrm{r},\{1,3\}}}\dfrac{\kappa_{\mathrm{r},\{1,3\}}}{\kappa_{\mathrm{l},\{1,3\}}}+1\right)-3\right]+1 \\ \dfrac{1}{3} = \dfrac{\sum_{\epsilon=\mathrm{l,r}} d_{\epsilon,\{1,1\}}/\kappa_{\epsilon,\{1,1\}}}{\sum_{j\#=1}^{3}\sum_{\epsilon=\mathrm{l,r}} d_{\epsilon,\{1,j\#\}}/\kappa_{\epsilon,\{1,j\#\}}} \\ \dfrac{1}{3} = \dfrac{\sum_{\epsilon=\mathrm{l,r}} d_{\epsilon,\{1,2\}}/\kappa_{\epsilon,\{1,2\}}}{\sum_{j\#=1}^{3}\sum_{\epsilon=\mathrm{l,r}} d_{\epsilon,\{1,j\#\}}/\kappa_{\epsilon,\{1,j\#\}}} \\ \dfrac{1}{3} = \dfrac{\sum_{\epsilon=\mathrm{l,r}} d_{\epsilon,\{1,3\}}/\kappa_{\epsilon,\{1,3\}}}{\sum_{j\#=1}^{3}\sum_{\epsilon=\mathrm{l,r}} d_{\epsilon,\{1,j\#\}}/\kappa_{\epsilon,\{1,j\#\}}} \end{cases} . \tag{S39}$$

For $i = 2$, we set $m = 2$

$$\begin{cases} 0.8 = \dfrac{1}{3}\left[1\Big/\left(\dfrac{d_{\mathrm{l},\{2,1\}}}{d_{\mathrm{r},\{2,1\}}}\dfrac{\kappa_{\mathrm{r},\{2,1\}}}{\kappa_{\mathrm{l},\{2,1\}}}+1\right)-1\right]+1 \\ 0.5 = \dfrac{1}{3}\left[1\Big/\left(\dfrac{d_{\mathrm{l},\{2,2\}}}{d_{\mathrm{r},\{2,2\}}}\dfrac{\kappa_{\mathrm{r},\{2,2\}}}{\kappa_{\mathrm{l},\{2,2\}}}+1\right)-2\right]+1 \\ 0.2 = \dfrac{1}{3}\left[1\Big/\left(\dfrac{d_{\mathrm{l},\{2,3\}}}{d_{\mathrm{r},\{2,3\}}}\dfrac{\kappa_{\mathrm{r},\{2,3\}}}{\kappa_{\mathrm{l},\{2,3\}}}+1\right)-3\right]+1 \\ \dfrac{1}{3} = \dfrac{\sum_{\epsilon=\mathrm{l,r}} d_{\epsilon,\{2,1\}}/\kappa_{\epsilon,\{2,1\}}}{\sum_{j\#=1}^{3}\sum_{\epsilon=\mathrm{l,r}} d_{\epsilon,\{2,j\#\}}/\kappa_{\epsilon,\{2,j\#\}}} \\ \dfrac{1}{3} = \dfrac{\sum_{\epsilon=\mathrm{l,r}} d_{\epsilon,\{2,2\}}/\kappa_{\epsilon,\{2,2\}}}{\sum_{j\#=1}^{3}\sum_{\epsilon=\mathrm{l,r}} d_{\epsilon,\{2,j\#\}}/\kappa_{\epsilon,\{2,j\#\}}} \\ \dfrac{1}{3} = \dfrac{\sum_{\epsilon=\mathrm{l,r}} d_{\epsilon,\{2,3\}}/\kappa_{\epsilon,\{2,3\}}}{\sum_{j\#=1}^{3}\sum_{\epsilon=\mathrm{l,r}} d_{\epsilon,\{2,j\#\}}/\kappa_{\epsilon,\{2,j\#\}}} \end{cases} . \tag{S40}$$

For $i = 3$, we set $m = 3$

$$\begin{cases} 0.7 = \dfrac{1}{3}\left[1\Big/\left(\dfrac{d_{\mathrm{l},\{3,1\}}}{d_{\mathrm{r},\{3,1\}}}\dfrac{\kappa_{\mathrm{r},\{3,1\}}}{\kappa_{\mathrm{l},\{3,1\}}}+1\right)-1\right]+1 \\ 0.4 = \dfrac{1}{3}\left[1\Big/\left(\dfrac{d_{\mathrm{l},\{3,2\}}}{d_{\mathrm{r},\{3,2\}}}\dfrac{\kappa_{\mathrm{r},\{3,2\}}}{\kappa_{\mathrm{l},\{3,2\}}}+1\right)-2\right]+1 \\ 0.1 = \dfrac{1}{3}\left[1\Big/\left(\dfrac{d_{\mathrm{l},\{3,3\}}}{d_{\mathrm{r},\{3,3\}}}\dfrac{\kappa_{\mathrm{r},\{3,3\}}}{\kappa_{\mathrm{l},\{3,3\}}}+1\right)-3\right]+1 \\ \dfrac{1}{3} = \dfrac{\sum_{\epsilon=\mathrm{l,r}} d_{\epsilon,\{3,1\}}/\kappa_{\epsilon,\{3,1\}}}{\sum_{j\#=1}^{3}\sum_{\epsilon=\mathrm{l,r}} d_{\epsilon,\{3,j\#\}}/\kappa_{\epsilon,\{3,j\#\}}} \\ \dfrac{1}{3} = \dfrac{\sum_{\epsilon=\mathrm{l,r}} d_{\epsilon,\{3,2\}}/\kappa_{\epsilon,\{3,2\}}}{\sum_{j\#=1}^{3}\sum_{\epsilon=\mathrm{l,r}} d_{\epsilon,\{3,j\#\}}/\kappa_{\epsilon,\{3,j\#\}}} \\ \dfrac{1}{3} = \dfrac{\sum_{\epsilon=\mathrm{l,r}} d_{\epsilon,\{3,3\}}/\kappa_{\epsilon,\{3,3\}}}{\sum_{j\#=1}^{3}\sum_{\epsilon=\mathrm{l,r}} d_{\epsilon,\{3,j\#\}}/\kappa_{\epsilon,\{3,j\#\}}} \end{cases} . \tag{S41}$$

In the calculation case, $\left[d_{\mathrm{l},\{i,j\}}\right]_{3\times 3} = \left[d_{\mathrm{r},\{i,j\}}\right]_{3\times 3} = [20]_{3\times 3}$ mm. We finally obtained

$$\left[\kappa_{\mathrm{l},\{i,j\}}\right]_{3\times 3} = \begin{bmatrix} 0.075 & 0.112 & 0.224 \\ 0.016 & 0.020 & 0.025 \\ 0.009 & 0.010 & 0.011 \end{bmatrix} \mathrm{~W~m^{-1}~K^{-1}}, \tag{S42}$$

and

$$\left[\kappa_{\mathrm{r},\{i,j\}}\right]_{3\times 3} = \begin{bmatrix} 0.032 & 0.028 & 0.025 \\ 0.025 & 0.020 & 0.016 \\ 0.077 & 0.039 & 0.026 \end{bmatrix} \mathrm{~W~m^{-1}~K^{-1}}. \tag{S43}$$

5.4 Approach 4

The above approaches are established on the temperature expression under the temperature-uniform condition. Actually, we can also use the original temperature expression to calculate the thermal/structural parameters without temperature-uniform condition.

According to Eq. (6) and Eq. (S25), for $i = 1$, we set $m = 1$,

$$\begin{cases} 390 = T_\mathrm{A} - \dfrac{T_\mathrm{A} - T_\mathrm{B}}{\sum_{j^\#=1}^{3}\sum_{\epsilon=\mathrm{l,c,r}} d_{\epsilon,\{1,j^\#\}}/\kappa_{\epsilon,\{1,j^\#\}}} \left(-\dfrac{d_{\mathrm{r},\{1,1\}}}{\kappa_{\mathrm{r},\{1,1\}}}\right. \\ \qquad \left.\left. - \dfrac{d_{\mathrm{c},\{1,1\}}/2 - x_{\{1,1\}}}{\kappa_{\mathrm{c},\{1,1\}}} + \displaystyle\sum_{j^*=1}^{1}\sum_{\epsilon=\mathrm{l,c,r}} \dfrac{d_{\epsilon,\{1,j^*\}}}{\kappa_{\epsilon,\{1,j^*\}}}\right)\right|_{x_{\{1,1\}}=0} \\ 360 = T_\mathrm{A} - \dfrac{T_\mathrm{A} - T_\mathrm{B}}{\sum_{j^\#=1}^{3}\sum_{\epsilon=\mathrm{l,c,r}} d_{\epsilon,\{1,j^\#\}}/\kappa_{\epsilon,\{1,j^\#\}}} \left(-\dfrac{d_{\mathrm{r},\{1,2\}}}{\kappa_{\mathrm{r},\{1,2\}}}\right. \\ \qquad \left.\left. - \dfrac{d_{\mathrm{c},\{1,2\}}/2 - x_{\{1,2\}}}{\kappa_{\mathrm{c},\{1,2\}}} + \displaystyle\sum_{j^*=1}^{2}\sum_{\epsilon=\mathrm{l,c,r}} \dfrac{d_{\epsilon,\{1,j^*\}}}{\kappa_{\epsilon,\{1,j^*\}}}\right)\right|_{x_{\{1,2\}}=0} \\ 330 = T_\mathrm{A} - \dfrac{T_\mathrm{A} - T_\mathrm{B}}{\sum_{j^\#=1}^{3}\sum_{\epsilon=\mathrm{l,c,r}} d_{\epsilon,\{1,j^\#\}}/\kappa_{\epsilon,\{1,j^\#\}}} \left(-\dfrac{d_{\mathrm{r},\{1,3\}}}{\kappa_{\mathrm{r},\{1,3\}}}\right. \\ \qquad \left.\left. - \dfrac{d_{\mathrm{c},\{1,3\}}/2 - x_{\{1,3\}}}{\kappa_{\mathrm{c},\{1,3\}}} + \displaystyle\sum_{j^*=1}^{3}\sum_{\epsilon=\mathrm{l,c,r}} \dfrac{d_{\epsilon,\{1,j^*\}}}{\kappa_{\epsilon,\{1,j^*\}}}\right)\right|_{x_{\{1,3\}}=0} \end{cases} . \tag{S44}$$

For $i = 2$, we set $m = 2$,

$$\begin{cases} 380 = T_\mathrm{A} - \dfrac{T_\mathrm{A} - T_\mathrm{B}}{\sum_{j^\#=1}^{3}\sum_{\epsilon=\mathrm{l,c,r}} d_{\epsilon,\{2,j^\#\}}/\kappa_{\epsilon,\{2,j^\#\}}} \left(-\dfrac{d_{\mathrm{r},\{2,1\}}}{\kappa_{\mathrm{r},\{2,1\}}}\right. \\ \qquad \left.\left. - \dfrac{d_{\mathrm{c},\{2,1\}}/2 - x_{\{2,1\}}}{\kappa_{\mathrm{c},\{2,1\}}} + \displaystyle\sum_{j^*=1}^{1}\sum_{\epsilon=\mathrm{l,c,r}} \dfrac{d_{\epsilon,\{2,j^*\}}}{\kappa_{\epsilon,\{2,j^*\}}}\right)\right|_{x_{\{2,1\}}=0} \\ 350 = T_\mathrm{A} - \dfrac{T_\mathrm{A} - T_\mathrm{B}}{\sum_{j^\#=1}^{3}\sum_{\epsilon=\mathrm{l,c,r}} d_{\epsilon,\{2,j^\#\}}/\kappa_{\epsilon,\{2,j^\#\}}} \left(-\dfrac{d_{\mathrm{r},\{2,2\}}}{\kappa_{\mathrm{r},\{2,2\}}}\right. \\ \qquad \left.\left. - \dfrac{d_{\mathrm{c},\{2,2\}}/2 - x_{\{2,2\}}}{\kappa_{\mathrm{c},\{2,2\}}} + \displaystyle\sum_{j^*=1}^{2}\sum_{\epsilon=\mathrm{l,c,r}} \dfrac{d_{\epsilon,\{2,j^*\}}}{\kappa_{\epsilon,\{2,j^*\}}}\right)\right|_{x_{\{2,2\}}=0} \\ 320 = T_\mathrm{A} - \dfrac{T_\mathrm{A} - T_\mathrm{B}}{\sum_{j^\#=1}^{3}\sum_{\epsilon=\mathrm{l,c,r}} d_{\epsilon,\{2,j^\#\}}/\kappa_{\epsilon,\{2,j^\#\}}} \left(-\dfrac{d_{\mathrm{r},\{2,3\}}}{\kappa_{\mathrm{r},\{2,3\}}}\right. \\ \qquad \left.\left. - \dfrac{d_{\mathrm{c},\{2,3\}}/2 - x_{\{2,3\}}}{\kappa_{\mathrm{c},\{2,3\}}} + \displaystyle\sum_{j^*=1}^{3}\sum_{\epsilon=\mathrm{l,c,r}} \dfrac{d_{\epsilon,\{2,j^*\}}}{\kappa_{\epsilon,\{2,j^*\}}}\right)\right|_{x_{\{2,3\}}=0} \end{cases} . \tag{S45}$$

For $i = 3$, we set $m = 3$,

$$\begin{cases} 370 = T_\mathrm{A} - \dfrac{T_\mathrm{A} - T_\mathrm{B}}{\sum_{j^\#=1}^{3} \sum_{\epsilon=\mathrm{l,c,r}} d_{\epsilon,\{3,j^\#\}}/\kappa_{\epsilon,\{3,j^\#\}}} \left(-\dfrac{d_{\mathrm{r},\{3,1\}}}{\kappa_{\mathrm{r},\{3,1\}}} \right. \\ \quad \left. \left. -\dfrac{d_{\mathrm{c},\{3,1\}}/2 - x_{\{3,1\}}}{\kappa_{\mathrm{c},\{3,1\}}} + \sum_{j^*=1}^{1} \sum_{\epsilon=\mathrm{l,c,r}} \dfrac{d_{\epsilon,\{3,j^*\}}}{\kappa_{\epsilon,\{3,j^*\}}} \right) \right|_{x_{\{3,1\}}=0} \\ 340 = T_\mathrm{A} - \dfrac{T_\mathrm{A} - T_\mathrm{B}}{\sum_{j^\#=1}^{3} \sum_{\epsilon=\mathrm{l,c,r}} d_{\epsilon,\{3,j^\#\}}/\kappa_{\epsilon,\{3,j^\#\}}} \left(-\dfrac{d_{\mathrm{r},\{3,2\}}}{\kappa_{\mathrm{r},\{3,2\}}} \right. \\ \quad \left. \left. -\dfrac{d_{\mathrm{c},\{3,2\}}/2 - x_{\{3,2\}}}{\kappa_{\mathrm{c},\{3,2\}}} + \sum_{j^*=1}^{2} \sum_{\epsilon=\mathrm{l,c,r}} \dfrac{d_{\epsilon,\{3,j^*\}}}{\kappa_{\epsilon,\{3,j^*\}}} \right) \right|_{x_{\{3,2\}}=0} \\ 310 = T_\mathrm{A} - \dfrac{T_\mathrm{A} - T_\mathrm{B}}{\sum_{j^\#=1}^{3} \sum_{\epsilon=\mathrm{l,c,r}} d_{\epsilon,\{3,j^\#\}}/\kappa_{\epsilon,\{3,j^\#\}}} \left(-\dfrac{d_{\mathrm{r},\{3,3\}}}{\kappa_{\mathrm{r},\{3,3\}}} \right. \\ \quad \left. \left. -\dfrac{d_{\mathrm{c},\{3,3\}}/2 - x_{\{3,3\}}}{\kappa_{\mathrm{c},\{3,3\}}} + \sum_{j^*=1}^{3} \sum_{\epsilon=\mathrm{l,c,r}} \dfrac{d_{\epsilon,\{3,j^*\}}}{\kappa_{\epsilon,\{3,j^*\}}} \right) \right|_{x_{\{3,3\}}=0} \end{cases} . \tag{S46}$$

Following the same operations as approach 1, we finally got

$$\left[\kappa_{\mathrm{l},\{i,j\}}\right]_{3\times 3} = \begin{bmatrix} 0.050 & 0.050 & 0.050 \\ 0.050 & 0.050 & 0.050 \\ 0.017 & 0.025 & 0.025 \end{bmatrix} \ \mathrm{W\ m^{-1}\ K^{-1}}, \tag{S47}$$

and

$$\left[\kappa_{\mathrm{r},\{i,j\}}\right]_{3\times 3} = \begin{bmatrix} 0.025 & 0.025 & 0.017 \\ 0.101 & 0.101 & 0.050 \\ 0.050 & 0.050 & 0.050 \end{bmatrix} \ \mathrm{W\ m^{-1}\ K^{-1}}. \tag{S48}$$

5.5 Approach 5

The following discusses the approach 2 without temperature-uniform condition. According to Eq. (S19), Eq. (S21), Eq. (S25), and Eq. (S33), for $i = 1$, we set $m = 1$,

$$\begin{cases} 0.9 = \theta_{\mathrm{c},\{1,1\}} \mathit{\Lambda}_{\{1,1\}} + 1 - \sum_{j^*=1}^{1} \mathit{\Lambda}_{\{1,j^*\}} \\ 0.6 = \theta_{\mathrm{c},\{1,2\}} \mathit{\Lambda}_{\{1,2\}} + 1 - \sum_{j^*=1}^{2} \mathit{\Lambda}_{\{1,j^*\}} \\ 0.3 = \theta_{\mathrm{c},\{1,3\}} \mathit{\Lambda}_{\{1,3\}} + 1 - \sum_{j^*=1}^{3} \mathit{\Lambda}_{\{1,j^*\}} \\ \theta_{\mathrm{c},\{1,1\}} = \left[\left(\dfrac{d_{\mathrm{r},\{1,1\}}}{\kappa_{\mathrm{r},\{1,1\}}} + \dfrac{d_{\mathrm{c},\{1,1\}}/2 - x_{\{1,1\}}}{\kappa_{\mathrm{c},\{1,1\}}} \right) \Big/ \left(\sum_{\epsilon=\mathrm{l,c,r}} \dfrac{d_{\epsilon,\{1,1\}}}{\kappa_{\epsilon,\{1,1\}}} \right) \right] \Bigg|_{x_{\{1,1\}}=0} \\ \theta_{\mathrm{c},\{1,2\}} = \left[\left(\dfrac{d_{\mathrm{r},\{1,2\}}}{\kappa_{\mathrm{r},\{1,2\}}} + \dfrac{d_{\mathrm{c},\{1,2\}}/2 - x_{\{1,2\}}}{\kappa_{\mathrm{c},\{1,2\}}} \right) \Big/ \left(\sum_{\epsilon=\mathrm{l,c,r}} \dfrac{d_{\epsilon,\{1,2\}}}{\kappa_{\epsilon,\{1,2\}}} \right) \right] \Bigg|_{x_{\{1,2\}}=0} \\ \theta_{\mathrm{c},\{1,3\}} = \left[\left(\dfrac{d_{\mathrm{r},\{1,3\}}}{\kappa_{\mathrm{r},\{1,3\}}} + \dfrac{d_{\mathrm{c},\{1,3\}}/2 - x_{\{1,3\}}}{\kappa_{\mathrm{c},\{1,3\}}} \right) \Big/ \left(\sum_{\epsilon=\mathrm{l,c,r}} \dfrac{d_{\epsilon,\{1,3\}}}{\kappa_{\epsilon,\{1,3\}}} \right) \right] \Bigg|_{x_{\{1,3\}}=0} \\ \mathit{\Lambda}_{\{1,1\}} = \dfrac{\sum_{\epsilon=\mathrm{l,c,r}} d_{\epsilon,\{1,1\}}/\kappa_{\epsilon,\{1,1\}}}{\sum_{j^\#=1}^{3} \sum_{\epsilon=\mathrm{l,c,r}} d_{\epsilon,\{1,j^\#\}}/\kappa_{\epsilon,\{1,j^\#\}}} \\ \mathit{\Lambda}_{\{1,2\}} = \dfrac{\sum_{\epsilon=\mathrm{l,c,r}} d_{\epsilon,\{1,2\}}/\kappa_{\epsilon,\{1,2\}}}{\sum_{j^\#=1}^{3} \sum_{\epsilon=\mathrm{l,c,r}} d_{\epsilon,\{1,j^\#\}}/\kappa_{\epsilon,\{1,j^\#\}}} \\ \mathit{\Lambda}_{\{1,3\}} = \dfrac{\sum_{\epsilon=\mathrm{l,c,r}} d_{\epsilon,\{1,3\}}/\kappa_{\epsilon,\{1,3\}}}{\sum_{j^\#=1}^{3} \sum_{\epsilon=\mathrm{l,c,r}} d_{\epsilon,\{1,j^\#\}}/\kappa_{\epsilon,\{1,j^\#\}}} \end{cases} . \quad \text{(S49)}$$

For $i = 2$, we set $m = 2$,

$$\begin{cases} 0.8 = \theta_{\mathrm{c},\{2,1\}}\Lambda_{\{2,1\}} + 1 - \sum_{j^*=1}^{1} \Lambda_{\{2,j^*\}} \\ 0.5 = \theta_{\mathrm{c},\{2,2\}}\Lambda_{\{2,2\}} + 1 - \sum_{j^*=1}^{2} \Lambda_{\{2,j^*\}} \\ 0.2 = \theta_{\mathrm{c},\{2,3\}}\Lambda_{\{2,3\}} + 1 - \sum_{j^*=1}^{3} \Lambda_{\{2,j^*\}} \\ \theta_{\mathrm{c},\{2,1\}} = \left[\left(\frac{d_{\mathrm{r},\{2,1\}}}{\kappa_{\mathrm{r},\{2,1\}}} + \frac{d_{\mathrm{c},\{2,1\}}/2 - x_{\{2,1\}}}{\kappa_{\mathrm{c},\{2,1\}}} \right) \Bigg/ \left(\sum_{\epsilon=\mathrm{l,c,r}} \frac{d_{\epsilon,\{2,1\}}}{\kappa_{\epsilon,\{2,1\}}} \right) \right] \Bigg|_{x_{\{2,1\}}=0} \\ \theta_{\mathrm{c},\{2,2\}} = \left[\left(\frac{d_{\mathrm{r},\{2,2\}}}{\kappa_{\mathrm{r},\{2,2\}}} + \frac{d_{\mathrm{c},\{2,2\}}/2 - x_{\{2,2\}}}{\kappa_{\mathrm{c},\{2,2\}}} \right) \Bigg/ \left(\sum_{\epsilon=\mathrm{l,c,r}} \frac{d_{\epsilon,\{2,2\}}}{\kappa_{\epsilon,\{2,2\}}} \right) \right] \Bigg|_{x_{\{2,2\}}=0} \\ \theta_{\mathrm{c},\{2,3\}} = \left[\left(\frac{d_{\mathrm{r},\{2,3\}}}{\kappa_{\mathrm{r},\{2,3\}}} + \frac{d_{\mathrm{c},\{2,3\}}/2 - x_{\{2,3\}}}{\kappa_{\mathrm{c},\{2,3\}}} \right) \Bigg/ \left(\sum_{\epsilon=\mathrm{l,c,r}} \frac{d_{\epsilon,\{2,3\}}}{\kappa_{\epsilon,\{2,3\}}} \right) \right] \Bigg|_{x_{\{2,3\}}=0} \\ \Lambda_{\{2,1\}} = \frac{\sum_{\epsilon=\mathrm{l,c,r}} d_{\epsilon,\{2,1\}}/\kappa_{\epsilon,\{2,1\}}}{\sum_{j^\#=1}^{3} \sum_{\epsilon=\mathrm{l,c,r}} d_{\epsilon,\{2,j^\#\}}/\kappa_{\epsilon,\{2,j^\#\}}} \\ \Lambda_{\{2,2\}} = \frac{\sum_{\epsilon=\mathrm{l,c,r}} d_{\epsilon,\{2,2\}}/\kappa_{\epsilon,\{2,2\}}}{\sum_{j^\#=1}^{3} \sum_{\epsilon=\mathrm{l,c,r}} d_{\epsilon,\{2,j^\#\}}/\kappa_{\epsilon,\{2,j^\#\}}} \\ \Lambda_{\{2,3\}} = \frac{\sum_{\epsilon=\mathrm{l,c,r}} d_{\epsilon,\{2,3\}}/\kappa_{\epsilon,\{2,3\}}}{\sum_{j^\#=1}^{3} \sum_{\epsilon=\mathrm{l,c,r}} d_{\epsilon,\{2,j^\#\}}/\kappa_{\epsilon,\{2,j^\#\}}} \end{cases} . \tag{S50}$$

For $i=3$, we set $m=3$,

$$\begin{cases} 0.7=\theta_{\mathrm{c},\{3,1\}}\mathit{\Lambda}_{\{3,1\}}+1-\sum_{j^*=1}^{1}\mathit{\Lambda}_{\{3,j^*\}} \\ 0.4=\theta_{\mathrm{c},\{3,2\}}\mathit{\Lambda}_{\{3,2\}}+1-\sum_{j^*=1}^{2}\mathit{\Lambda}_{\{3,j^*\}} \\ 0.1=\theta_{\mathrm{c},\{3,3\}}\mathit{\Lambda}_{\{3,3\}}+1-\sum_{j^*=1}^{3}\mathit{\Lambda}_{\{3,j^*\}} \\ \theta_{\mathrm{c},\{3,1\}}=\left.\left[\left(\frac{d_{\mathrm{r},\{3,1\}}}{\kappa_{\mathrm{r},\{3,1\}}}+\frac{d_{\mathrm{c},\{3,1\}}/2-x_{\{3,1\}}}{\kappa_{\mathrm{c},\{3,1\}}}\right)\Big/\left(\sum_{\epsilon=\mathrm{l,c,r}}\frac{d_{\epsilon,\{3,1\}}}{\kappa_{\epsilon,\{3,1\}}}\right)\right]\right|_{x_{\{3,1\}}=0} \\ \theta_{\mathrm{c},\{3,2\}}=\left.\left[\left(\frac{d_{\mathrm{r},\{3,2\}}}{\kappa_{\mathrm{r},\{3,2\}}}+\frac{d_{\mathrm{c},\{3,2\}}/2-x_{\{3,2\}}}{\kappa_{\mathrm{c},\{3,2\}}}\right)\Big/\left(\sum_{\epsilon=\mathrm{l,c,r}}\frac{d_{\epsilon,\{3,2\}}}{\kappa_{\epsilon,\{3,2\}}}\right)\right]\right|_{x_{\{3,2\}}=0} \\ \theta_{\mathrm{c},\{3,3\}}=\left.\left[\left(\frac{d_{\mathrm{r},\{3,3\}}}{\kappa_{\mathrm{r},\{3,3\}}}+\frac{d_{\mathrm{c},\{3,3\}}/2-x_{\{3,3\}}}{\kappa_{\mathrm{c},\{3,3\}}}\right)\Big/\left(\sum_{\epsilon=\mathrm{l,c,r}}\frac{d_{\epsilon,\{3,3\}}}{\kappa_{\epsilon,\{3,3\}}}\right)\right]\right|_{x_{\{3,3\}}=0} \\ \mathit{\Lambda}_{\{3,1\}}=\frac{\sum_{\epsilon=\mathrm{l,c,r}}d_{\epsilon,\{3,1\}}/\kappa_{\epsilon,\{3,1\}}}{\sum_{j^\#=1}^{3}\sum_{\epsilon=\mathrm{l,c,r}}d_{\epsilon,\{3,j^\#\}}/\kappa_{\epsilon,\{3,j^\#\}}} \\ \mathit{\Lambda}_{\{3,2\}}=\frac{\sum_{\epsilon=\mathrm{l,c,r}}d_{\epsilon,\{3,2\}}/\kappa_{\epsilon,\{3,2\}}}{\sum_{j^\#=1}^{3}\sum_{\epsilon=\mathrm{l,c,r}}d_{\epsilon,\{3,j^\#\}}/\kappa_{\epsilon,\{3,j^\#\}}} \\ \mathit{\Lambda}_{\{3,3\}}=\frac{\sum_{\epsilon=\mathrm{l,c,r}}d_{\epsilon,\{3,3\}}/\kappa_{\epsilon,\{3,3\}}}{\sum_{j^\#=1}^{3}\sum_{\epsilon=\mathrm{l,c,r}}d_{\epsilon,\{3,j^\#\}}/\kappa_{\epsilon,\{3,j^\#\}}} \end{cases}. \tag{S51}$$

Setting $\mathit{\Lambda}_{\{i,j\}}=1/3$, we finally obtained the results.

5.6 Approach 6

The above approaches are constructed mainly to calculate the thermal/structural parameters of the multi-temperature control system. The following gives another method to obtain more parameters according to Eq. (5a), Eq. (6), and Eq. (S16).

For $i=1$, we set $m=1$

$$\begin{cases} q_1 = \dfrac{T_\mathrm{A}-T_\mathrm{B}}{\sum_{j^\#=1}^{3}\sum_{\epsilon=\mathrm{l,c,r}} d_{\epsilon,\{1,j^\#\}}/\kappa_{\epsilon,\{1,j^\#\}}} \\ T^{+}_{\mathrm{c},\{1,1\}} = T_\mathrm{A} - q_1\left(-\dfrac{d_{\mathrm{r},\{1,1\}}}{\kappa_{\mathrm{r},\{1,1\}}} - \dfrac{d_{\mathrm{c},\{1,1\}}/2 - x_{\{1,1\}}}{\kappa_{\mathrm{c},\{1,1\}}} + \sum_{j^*=1}^{1}\sum_{\epsilon=\mathrm{l,c,r}}\dfrac{d_{\epsilon,\{1,j^*\}}}{\kappa_{\epsilon,\{1,j^*\}}}\right)\Bigg|_{x_{\{1,1\}}=x^{+}_{\mathrm{c},\{1,1\}}} \\ T^{0}_{\mathrm{c},\{1,1\}} = T_\mathrm{A} - q_1\left(-\dfrac{d_{\mathrm{r},\{1,1\}}}{\kappa_{\mathrm{r},\{1,1\}}} - \dfrac{d_{\mathrm{c},\{1,1\}}/2 - x_{\{1,1\}}}{\kappa_{\mathrm{c},\{1,1\}}} + \sum_{j^*=1}^{1}\sum_{\epsilon=\mathrm{l,c,r}}\dfrac{d_{\epsilon,\{1,j^*\}}}{\kappa_{\epsilon,\{1,j^*\}}}\right)\Bigg|_{x_{\{1,1\}}=0} \\ T^{-}_{\mathrm{c},\{1,1\}} = T_\mathrm{A} - q_1\left(-\dfrac{d_{\mathrm{r},\{1,1\}}}{\kappa_{\mathrm{r},\{1,1\}}} - \dfrac{d_{\mathrm{c},\{1,1\}}/2 - x_{\{1,1\}}}{\kappa_{\mathrm{c},\{1,1\}}} + \sum_{j^*=1}^{1}\sum_{\epsilon=\mathrm{l,c,r}}\dfrac{d_{\epsilon,\{1,j^*\}}}{\kappa_{\epsilon,\{1,j^*\}}}\right)\Bigg|_{x_{\{1,1\}}=x^{-}_{\mathrm{c},\{1,1\}}} \\ T^{+}_{\mathrm{c},\{1,2\}} = T_\mathrm{A} - q_1\left(-\dfrac{d_{\mathrm{r},\{1,2\}}}{\kappa_{\mathrm{r},\{1,2\}}} - \dfrac{d_{\mathrm{c},\{1,2\}}/2 - x_{\{1,2\}}}{\kappa_{\mathrm{c},\{1,2\}}} + \sum_{j^*=1}^{2}\sum_{\epsilon=\mathrm{l,c,r}}\dfrac{d_{\epsilon,\{1,j^*\}}}{\kappa_{\epsilon,\{1,j^*\}}}\right)\Bigg|_{x_{\{1,2\}}=x^{+}_{\mathrm{c},\{1,2\}}} \\ T^{0}_{\mathrm{c},\{1,2\}} = T_\mathrm{A} - q_1\left(-\dfrac{d_{\mathrm{r},\{1,2\}}}{\kappa_{\mathrm{r},\{1,2\}}} - \dfrac{d_{\mathrm{c},\{1,2\}}/2 - x_{\{1,2\}}}{\kappa_{\mathrm{c},\{1,2\}}} + \sum_{j^*=1}^{2}\sum_{\epsilon=\mathrm{l,c,r}}\dfrac{d_{\epsilon,\{1,j^*\}}}{\kappa_{\epsilon,\{1,j^*\}}}\right)\Bigg|_{x_{\{1,2\}}=0} \\ T^{-}_{\mathrm{c},\{1,2\}} = T_\mathrm{A} - q_1\left(-\dfrac{d_{\mathrm{r},\{1,2\}}}{\kappa_{\mathrm{r},\{1,2\}}} - \dfrac{d_{\mathrm{c},\{1,2\}}/2 - x_{\{1,2\}}}{\kappa_{\mathrm{c},\{1,2\}}} + \sum_{j^*=1}^{2}\sum_{\epsilon=\mathrm{l,c,r}}\dfrac{d_{\epsilon,\{1,j^*\}}}{\kappa_{\epsilon,\{1,j^*\}}}\right)\Bigg|_{x_{\{1,2\}}=x^{-}_{\mathrm{c},\{1,2\}}} \\ T^{+}_{\mathrm{c},\{1,3\}} = T_\mathrm{A} - q_1\left(-\dfrac{d_{\mathrm{r},\{1,3\}}}{\kappa_{\mathrm{r},\{1,3\}}} - \dfrac{d_{\mathrm{c},\{1,3\}}/2 - x_{\{1,3\}}}{\kappa_{\mathrm{c},\{1,3\}}} + \sum_{j^*=1}^{3}\sum_{\epsilon=\mathrm{l,c,r}}\dfrac{d_{\epsilon,\{1,j^*\}}}{\kappa_{\epsilon,\{1,j^*\}}}\right)\Bigg|_{x_{\{1,3\}}=x^{+}_{\mathrm{c},\{1,3\}}} \\ T^{0}_{\mathrm{c},\{1,3\}} = T_\mathrm{A} - q_1\left(-\dfrac{d_{\mathrm{r},\{1,3\}}}{\kappa_{\mathrm{r},\{1,3\}}} - \dfrac{d_{\mathrm{c},\{1,3\}}/2 - x_{\{1,3\}}}{\kappa_{\mathrm{c},\{1,3\}}} + \sum_{j^*=1}^{3}\sum_{\epsilon=\mathrm{l,c,r}}\dfrac{d_{\epsilon,\{1,j^*\}}}{\kappa_{\epsilon,\{1,j^*\}}}\right)\Bigg|_{x_{\{1,3\}}=0} \\ T^{-}_{\mathrm{c},\{1,3\}} = T_\mathrm{A} - q_1\left(-\dfrac{d_{\mathrm{r},\{1,3\}}}{\kappa_{\mathrm{r},\{1,3\}}} - \dfrac{d_{\mathrm{c},\{1,3\}}/2 - x_{\{1,3\}}}{\kappa_{\mathrm{c},\{1,3\}}} + \sum_{j^*=1}^{3}\sum_{\epsilon=\mathrm{l,c,r}}\dfrac{d_{\epsilon,\{1,j^*\}}}{\kappa_{\epsilon,\{1,j^*\}}}\right)\Bigg|_{x_{\{1,3\}}=x^{-}_{\mathrm{c},\{1,3\}}} \\ 0.9 = \dfrac{T^{0}_{\{1,1\}} - T_\mathrm{B}}{T_\mathrm{A}-T_\mathrm{B}} \\ 0.6 = \dfrac{T^{0}_{\{1,2\}} - T_\mathrm{B}}{T_\mathrm{A}-T_\mathrm{B}} \\ 0.3 = \dfrac{T^{0}_{\{1,3\}} - T_\mathrm{B}}{T_\mathrm{A}-T_\mathrm{B}} \end{cases} . \tag{S52}$$

For $i = 2$, we set $m = 2$

$$\begin{cases} q_2 = \dfrac{T_\mathrm{A} - T_\mathrm{B}}{\sum_{j^\#=1}^{3} \sum_{\epsilon=\mathrm{l,c,r}} d_{\epsilon,\{2,j^\#\}} / \kappa_{\epsilon,\{2,j^\#\}}} \\ T^+_{\mathrm{c},\{2,1\}} = T_\mathrm{A} - q_2 \left(-\dfrac{d_{\mathrm{r},\{2,1\}}}{\kappa_{\mathrm{r},\{2,1\}}} - \dfrac{d_{\mathrm{c},\{2,1\}}/2 - x_{\{2,1\}}}{\kappa_{\mathrm{c},\{2,1\}}} + \sum_{j^*=1}^{1} \sum_{\epsilon=\mathrm{l,c,r}} \dfrac{d_{\epsilon,\{2,j^*\}}}{\kappa_{\epsilon,\{2,j^*\}}} \right) \Bigg|_{x_{\{2,1\}}=x^+_{\mathrm{c},\{2,1\}}} \\ T^0_{\mathrm{c},\{2,1\}} = T_\mathrm{A} - q_2 \left(-\dfrac{d_{\mathrm{r},\{2,1\}}}{\kappa_{\mathrm{r},\{2,1\}}} - \dfrac{d_{\mathrm{c},\{2,1\}}/2 - x_{\{2,1\}}}{\kappa_{\mathrm{c},\{2,1\}}} + \sum_{j^*=1}^{1} \sum_{\epsilon=\mathrm{l,c,r}} \dfrac{d_{\epsilon,\{2,j^*\}}}{\kappa_{\epsilon,\{2,j^*\}}} \right) \Bigg|_{x_{\{2,1\}}=0} \\ T^-_{\mathrm{c},\{2,1\}} = T_\mathrm{A} - q_2 \left(-\dfrac{d_{\mathrm{r},\{2,1\}}}{\kappa_{\mathrm{r},\{2,1\}}} - \dfrac{d_{\mathrm{c},\{2,1\}}/2 - x_{\{2,1\}}}{\kappa_{\mathrm{c},\{2,1\}}} + \sum_{j^*=1}^{1} \sum_{\epsilon=\mathrm{l,c,r}} \dfrac{d_{\epsilon,\{2,j^*\}}}{\kappa_{\epsilon,\{2,j^*\}}} \right) \Bigg|_{x_{\{2,1\}}=x^-_{\mathrm{c},\{2,1\}}} \\ T^+_{\mathrm{c},\{2,2\}} = T_\mathrm{A} - q_2 \left(-\dfrac{d_{\mathrm{r},\{2,2\}}}{\kappa_{\mathrm{r},\{2,2\}}} - \dfrac{d_{\mathrm{c},\{2,2\}}/2 - x_{\{2,2\}}}{\kappa_{\mathrm{c},\{2,2\}}} + \sum_{j^*=1}^{2} \sum_{\epsilon=\mathrm{l,c,r}} \dfrac{d_{\epsilon,\{2,j^*\}}}{\kappa_{\epsilon,\{2,j^*\}}} \right) \Bigg|_{x_{\{2,2\}}=x^+_{\mathrm{c},\{2,2\}}} \\ T^0_{\mathrm{c},\{2,2\}} = T_\mathrm{A} - q_2 \left(-\dfrac{d_{\mathrm{r},\{2,2\}}}{\kappa_{\mathrm{r},\{2,2\}}} - \dfrac{d_{\mathrm{c},\{2,2\}}/2 - x_{\{2,2\}}}{\kappa_{\mathrm{c},\{2,2\}}} + \sum_{j^*=1}^{2} \sum_{\epsilon=\mathrm{l,c,r}} \dfrac{d_{\epsilon,\{2,j^*\}}}{\kappa_{\epsilon,\{2,j^*\}}} \right) \Bigg|_{x_{\{2,2\}}=0} \\ T^-_{\mathrm{c},\{2,2\}} = T_\mathrm{A} - q_2 \left(-\dfrac{d_{\mathrm{r},\{2,2\}}}{\kappa_{\mathrm{r},\{2,2\}}} - \dfrac{d_{\mathrm{c},\{2,2\}}/2 - x_{\{2,2\}}}{\kappa_{\mathrm{c},\{2,2\}}} + \sum_{j^*=1}^{2} \sum_{\epsilon=\mathrm{l,c,r}} \dfrac{d_{\epsilon,\{2,j^*\}}}{\kappa_{\epsilon,\{2,j^*\}}} \right) \Bigg|_{x_{\{2,2\}}=x^-_{\mathrm{c},\{2,2\}}} \\ T^+_{\mathrm{c},\{2,3\}} = T_\mathrm{A} - q_2 \left(-\dfrac{d_{\mathrm{r},\{2,3\}}}{\kappa_{\mathrm{r},\{2,3\}}} - \dfrac{d_{\mathrm{c},\{2,3\}}/2 - x_{\{2,3\}}}{\kappa_{\mathrm{c},\{2,3\}}} + \sum_{j^*=1}^{3} \sum_{\epsilon=\mathrm{l,c,r}} \dfrac{d_{\epsilon,\{2,j^*\}}}{\kappa_{\epsilon,\{2,j^*\}}} \right) \Bigg|_{x_{\{2,3\}}=x^+_{\mathrm{c},\{2,3\}}} \\ T^0_{\mathrm{c},\{2,3\}} = T_\mathrm{A} - q_2 \left(-\dfrac{d_{\mathrm{r},\{2,3\}}}{\kappa_{\mathrm{r},\{2,3\}}} - \dfrac{d_{\mathrm{c},\{2,3\}}/2 - x_{\{2,3\}}}{\kappa_{\mathrm{c},\{2,3\}}} + \sum_{j^*=1}^{3} \sum_{\epsilon=\mathrm{l,c,r}} \dfrac{d_{\epsilon,\{2,j^*\}}}{\kappa_{\epsilon,\{2,j^*\}}} \right) \Bigg|_{x_{\{2,3\}}=0} \\ T^-_{\mathrm{c},\{2,3\}} = T_\mathrm{A} - q_2 \left(-\dfrac{d_{\mathrm{r},\{2,3\}}}{\kappa_{\mathrm{r},\{2,3\}}} - \dfrac{d_{\mathrm{c},\{2,3\}}/2 - x_{\{2,3\}}}{\kappa_{\mathrm{c},\{2,3\}}} + \sum_{j^*=1}^{3} \sum_{\epsilon=\mathrm{l,c,r}} \dfrac{d_{\epsilon,\{2,j^*\}}}{\kappa_{\epsilon,\{2,j^*\}}} \right) \Bigg|_{x_{\{2,3\}}=x^-_{\mathrm{c},\{2,3\}}} \\ 0.8 = \dfrac{T^0_{\{2,1\}} - T_\mathrm{B}}{T_\mathrm{A} - T_\mathrm{B}} \\ 0.5 = \dfrac{T^0_{\{2,2\}} - T_\mathrm{B}}{T_\mathrm{A} - T_\mathrm{B}} \\ 0.2 = \dfrac{T^0_{\{2,3\}} - T_\mathrm{B}}{T_\mathrm{A} - T_\mathrm{B}} \end{cases} . \tag{S53}$$

For $i = 3$, we set $m = 3$

$$\begin{cases} q_3 = \dfrac{T_\mathrm{A} - T_\mathrm{B}}{\sum_{j^\#=1}^{3} \sum_{\epsilon=\mathrm{l,c,r}} d_{\epsilon,\{3,j^\#\}} / \kappa_{\epsilon,\{3,j^\#\}}} \\ T^+_{\mathrm{c},\{3,1\}} = T_\mathrm{A} - q_3 \left(-\dfrac{d_{\mathrm{r},\{3,1\}}}{\kappa_{\mathrm{r},\{3,1\}}} - \dfrac{d_{\mathrm{c},\{3,1\}}/2 - x_{\{3,1\}}}{\kappa_{\mathrm{c},\{3,1\}}} + \sum_{j^*=1}^{1} \sum_{\epsilon=\mathrm{l,c,r}} \dfrac{d_{\epsilon,\{3,j^*\}}}{\kappa_{\epsilon,\{3,j^*\}}} \right) \Bigg|_{x_{\{3,1\}} = x^+_{\mathrm{c},\{3,1\}}} \\ T^0_{\mathrm{c},\{3,1\}} = T_\mathrm{A} - q_3 \left(-\dfrac{d_{\mathrm{r},\{3,1\}}}{\kappa_{\mathrm{r},\{3,1\}}} - \dfrac{d_{\mathrm{c},\{3,1\}}/2 - x_{\{3,1\}}}{\kappa_{\mathrm{c},\{3,1\}}} + \sum_{j^*=1}^{1} \sum_{\epsilon=\mathrm{l,c,r}} \dfrac{d_{\epsilon,\{3,j^*\}}}{\kappa_{\epsilon,\{3,j^*\}}} \right) \Bigg|_{x_{\{3,1\}} = 0} \\ T^-_{\mathrm{c},\{3,1\}} = T_\mathrm{A} - q_3 \left(-\dfrac{d_{\mathrm{r},\{3,1\}}}{\kappa_{\mathrm{r},\{3,1\}}} - \dfrac{d_{\mathrm{c},\{3,1\}}/2 - x_{\{3,1\}}}{\kappa_{\mathrm{c},\{3,1\}}} + \sum_{j^*=1}^{1} \sum_{\epsilon=\mathrm{l,c,r}} \dfrac{d_{\epsilon,\{3,j^*\}}}{\kappa_{\epsilon,\{3,j^*\}}} \right) \Bigg|_{x_{\{3,1\}} = x^-_{\mathrm{c},\{3,1\}}} \\ T^+_{\mathrm{c},\{3,2\}} = T_\mathrm{A} - q_3 \left(-\dfrac{d_{\mathrm{r},\{3,2\}}}{\kappa_{\mathrm{r},\{3,2\}}} - \dfrac{d_{\mathrm{c},\{3,2\}}/2 - x_{\{3,2\}}}{\kappa_{\mathrm{c},\{3,2\}}} + \sum_{j^*=1}^{2} \sum_{\epsilon=\mathrm{l,c,r}} \dfrac{d_{\epsilon,\{3,j^*\}}}{\kappa_{\epsilon,\{3,j^*\}}} \right) \Bigg|_{x_{\{3,2\}} = x^+_{\mathrm{c},\{3,2\}}} \\ T^0_{\mathrm{c},\{3,2\}} = T_\mathrm{A} - q_3 \left(-\dfrac{d_{\mathrm{r},\{3,2\}}}{\kappa_{\mathrm{r},\{3,2\}}} - \dfrac{d_{\mathrm{c},\{3,2\}}/2 - x_{\{3,2\}}}{\kappa_{\mathrm{c},\{3,2\}}} + \sum_{j^*=1}^{2} \sum_{\epsilon=\mathrm{l,c,r}} \dfrac{d_{\epsilon,\{3,j^*\}}}{\kappa_{\epsilon,\{3,j^*\}}} \right) \Bigg|_{x_{\{3,2\}} = 0} \\ T^-_{\mathrm{c},\{3,2\}} = T_\mathrm{A} - q_3 \left(-\dfrac{d_{\mathrm{r},\{3,2\}}}{\kappa_{\mathrm{r},\{3,2\}}} - \dfrac{d_{\mathrm{c},\{3,2\}}/2 - x_{\{3,2\}}}{\kappa_{\mathrm{c},\{3,2\}}} + \sum_{j^*=1}^{2} \sum_{\epsilon=\mathrm{l,c,r}} \dfrac{d_{\epsilon,\{3,j^*\}}}{\kappa_{\epsilon,\{3,j^*\}}} \right) \Bigg|_{x_{\{3,2\}} = x^-_{\mathrm{c},\{3,2\}}} \\ T^+_{\mathrm{c},\{3,3\}} = T_\mathrm{A} - q_3 \left(-\dfrac{d_{\mathrm{r},\{3,3\}}}{\kappa_{\mathrm{r},\{3,3\}}} - \dfrac{d_{\mathrm{c},\{3,3\}}/2 - x_{\{3,3\}}}{\kappa_{\mathrm{c},\{3,3\}}} + \sum_{j^*=1}^{3} \sum_{\epsilon=\mathrm{l,c,r}} \dfrac{d_{\epsilon,\{3,j^*\}}}{\kappa_{\epsilon,\{3,j^*\}}} \right) \Bigg|_{x_{\{3,3\}} = x^+_{\mathrm{c},\{3,3\}}} \\ T^0_{\mathrm{c},\{3,3\}} = T_\mathrm{A} - q_3 \left(-\dfrac{d_{\mathrm{r},\{3,3\}}}{\kappa_{\mathrm{r},\{3,3\}}} - \dfrac{d_{\mathrm{c},\{3,3\}}/2 - x_{\{3,3\}}}{\kappa_{\mathrm{c},\{3,3\}}} + \sum_{j^*=1}^{3} \sum_{\epsilon=\mathrm{l,c,r}} \dfrac{d_{\epsilon,\{3,j^*\}}}{\kappa_{\epsilon,\{3,j^*\}}} \right) \Bigg|_{x_{\{3,3\}} = 0} \\ T^-_{\mathrm{c},\{3,3\}} = T_\mathrm{A} - q_3 \left(-\dfrac{d_{\mathrm{r},\{3,3\}}}{\kappa_{\mathrm{r},\{3,3\}}} - \dfrac{d_{\mathrm{c},\{3,3\}}/2 - x_{\{3,3\}}}{\kappa_{\mathrm{c},\{3,3\}}} + \sum_{j^*=1}^{3} \sum_{\epsilon=\mathrm{l,c,r}} \dfrac{d_{\epsilon,\{3,j^*\}}}{\kappa_{\epsilon,\{3,j^*\}}} \right) \Bigg|_{x_{\{3,3\}} = x^-_{\mathrm{c},\{3,3\}}} \\ 0.7 = \dfrac{T^0_{\{3,1\}} - T_\mathrm{B}}{T_\mathrm{A} - T_\mathrm{B}} \\ 0.4 = \dfrac{T^0_{\{3,2\}} - T_\mathrm{B}}{T_\mathrm{A} - T_\mathrm{B}} \\ 0.1 = \dfrac{T^0_{\{3,3\}} - T_\mathrm{B}}{T_\mathrm{A} - T_\mathrm{B}} \end{cases} . \tag{S54}$$

According to the calculation case (Supplementary Note 4), we obtained $T_\mathrm{A} = 400$ K, $T_\mathrm{B} = 300$ K, and $\left[d_{\mathrm{l},\{i,j\}}\right]_{3\times3} = \left[d_{\mathrm{r},\{i,j\}}\right]_{3\times3} = [20]_{3\times3}$ mm. Setting $\kappa_{\mathrm{l},\{1,1\}} = \kappa_{\mathrm{l},\{1,2\}} = \kappa_{\mathrm{l},\{1,3\}} =$

$0.050\ \mathrm{W\ m^{-1}\ K^{-1}}$, Eq. (S52) was solved. We have

$$\begin{cases} q_1 = 24.845\ \mathrm{W\ m^{-2}} \\ T^{+}_{\mathrm{c},\{1,1\}} = 389.938\ \mathrm{K} \\ T^{0}_{\mathrm{c},\{1,1\}} = 390.000\ \mathrm{K} \\ T^{-}_{\mathrm{c},\{1,1\}} = 390.062\ \mathrm{K} \\ T^{+}_{\mathrm{c},\{1,2\}} = 359.938\ \mathrm{K} \\ T^{0}_{\mathrm{c},\{1,2\}} = 360.000\ \mathrm{K} \\ T^{-}_{\mathrm{c},\{1,2\}} = 360.062\ \mathrm{K} \\ T^{+}_{\mathrm{c},\{1,3\}} = 329.938\ \mathrm{K} \\ T^{0}_{\mathrm{c},\{1,3\}} = 330.000\ \mathrm{K} \\ T^{-}_{\mathrm{c},\{1,3\}} = 330.062\ \mathrm{K} \\ \kappa_{\mathrm{r},\{1,1\}} = 0.025\ \mathrm{W\ m^{-1}\ K^{-1}} \\ \kappa_{\mathrm{r},\{1,2\}} = 0.025\ \mathrm{W\ m^{-1}\ K^{-1}} \\ \kappa_{\mathrm{r},\{1,3\}} = 0.017\ \mathrm{W\ m^{-1}\ K^{-1}} \end{cases} . \tag{S55}$$

Setting $\kappa_{\mathrm{l},\{2,1\}} = \kappa_{\mathrm{l},\{2,2\}} = \kappa_{\mathrm{l},\{2,3\}} = 0.050\ \mathrm{W\ m^{-1}\ K^{-1}}$, Eq. (S53) was solved. We have

$$\begin{cases} q_2 = 49.689\ \mathrm{W\ m^{-2}} \\ T^{+}_{\mathrm{c},\{2,1\}} = 379.876\ \mathrm{K} \\ T^{0}_{\mathrm{c},\{2,1\}} = 380.000\ \mathrm{K} \\ T^{-}_{\mathrm{c},\{2,1\}} = 380.124\ \mathrm{K} \\ T^{+}_{\mathrm{c},\{2,2\}} = 349.876\ \mathrm{K} \\ T^{0}_{\mathrm{c},\{2,2\}} = 350.000\ \mathrm{K} \\ T^{-}_{\mathrm{c},\{2,2\}} = 350.124\ \mathrm{K} \\ T^{+}_{\mathrm{c},\{2,3\}} = 319.876\ \mathrm{K} \\ T^{0}_{\mathrm{c},\{2,3\}} = 320.000\ \mathrm{K} \\ T^{-}_{\mathrm{c},\{2,3\}} = 320.124\ \mathrm{K} \\ \kappa_{\mathrm{r},\{2,1\}} = 0.101\ \mathrm{W\ m^{-1}\ K^{-1}} \\ \kappa_{\mathrm{r},\{2,2\}} = 0.101\ \mathrm{W\ m^{-1}\ K^{-1}} \\ \kappa_{\mathrm{r},\{2,3\}} = 0.050\ \mathrm{W\ m^{-1}\ K^{-1}} \end{cases} . \tag{S56}$$

Setting $\kappa_{\mathrm{r},\{3,1\}} = \kappa_{\mathrm{r},\{3,2\}} = \kappa_{\mathrm{r},\{3,3\}} = 0.050\ \mathrm{W\ m^{-1}\ K^{-1}}$, Eq. (S54) was solved. We have

$$\begin{cases} q_3 = 24.845\ \mathrm{W\ m^{-2}} \\ T^{+}_{\mathrm{c},\{3,1\}} = 369.938\ \mathrm{K} \\ T^{0}_{\mathrm{c},\{3,1\}} = 370.000\ \mathrm{K} \\ T^{-}_{\mathrm{c},\{3,1\}} = 370.062\ \mathrm{K} \\ T^{+}_{\mathrm{c},\{3,2\}} = 339.938\ \mathrm{K} \\ T^{0}_{\mathrm{c},\{3,2\}} = 340.000\ \mathrm{K} \\ T^{-}_{\mathrm{c},\{3,2\}} = 340.062\ \mathrm{K} \\ T^{+}_{\mathrm{c},\{3,3\}} = 309.938\ \mathrm{K} \\ T^{0}_{\mathrm{c},\{3,3\}} = 310.000\ \mathrm{K} \\ T^{-}_{\mathrm{c},\{3,3\}} = 310.062\ \mathrm{K} \\ \kappa_{\mathrm{r},\{3,1\}} = 0.017\ \mathrm{W\ m^{-1}\ K^{-1}} \\ \kappa_{\mathrm{r},\{3,2\}} = 0.025\ \mathrm{W\ m^{-1}\ K^{-1}} \\ \kappa_{\mathrm{r},\{3,3\}} = 0.025\ \mathrm{W\ m^{-1}\ K^{-1}} \end{cases}. \tag{S57}$$

Arranging the thermal parameters, we got

$$\left[\kappa_{\mathrm{l},\{i,j\}}\right]_{3\times 3} = \begin{bmatrix} 0.050 & 0.050 & 0.050 \\ 0.050 & 0.050 & 0.050 \\ 0.017 & 0.025 & 0.025 \end{bmatrix}\ \mathrm{W\ m^{-1}\ K^{-1}}, \tag{S58}$$

and

$$\left[\kappa_{\mathrm{r},\{i,j\}}\right]_{3\times 3} = \begin{bmatrix} 0.025 & 0.025 & 0.017 \\ 0.101 & 0.101 & 0.050 \\ 0.050 & 0.050 & 0.050 \end{bmatrix}\ \mathrm{W\ m^{-1}\ K^{-1}}. \tag{S59}$$

The precise temperature control in $x_{\{i,j\}} = 0$ and the low dispersion among $T^{+}_{\mathrm{c},\{i,j\}}$, $T^{0}_{\mathrm{c},\{i,j\}}$, and $T^{-}_{\mathrm{c},\{i,j\}}$ indicates that the multi-temperature control system has an excellent multi-temperature control capability.

5.7 Summary

In this section, we introduced six approaches to determine the thermal parameters of the multi-temperature control system. Leveraging these solutions, we designed multi-temperature control systems and executed associated finite element simulations. The maximum/average/minimum temperatures of the goods stored in the temperature control zones are presented in Supplementary Table 1. The low discrepancies between the simulation results and the multi-temperature requirements [Eq. (S25)] affirm the accuracy of all the approaches.

6 Arbitrary and precise multi-temperature control capability of AMTC

Regarding the multi-temperature control system illustrated in Supplementary Fig. 2a1, the temperature $T_{\mathrm{o},\{i,j\}}$ can be adaptively adjusted through the modification of thermal parameters, namely $\kappa_{\mathrm{l},\{i,j\}}$ and $\kappa_{\mathrm{r},\{i,j\}}$, to meet diverse temperature requirements. A few scenarios are enumerated below:

$$\begin{cases} \left[T_{\mathrm{o},\{i,j\},\mathrm{re}}\right]_{3\times3} = \begin{bmatrix} 395 & 365 & 330 \\ 385 & 355 & 325 \\ 375 & 345 & 315 \end{bmatrix} \mathrm{K} \\ \left[\kappa_{\mathrm{l},\{i,j\}}\right]_{3\times3} = \begin{bmatrix} 0.640 & 2.286 & 1.000 \\ 0.149 & 0.193 & 0.273 \\ 0.060 & 0.069 & 0.082 \end{bmatrix} \mathrm{W\,m^{-1}\,K^{-1}} \qquad \text{Case 1,} \\ \left[\kappa_{\mathrm{r},\{i,j\}}\right]_{3\times3} = \begin{bmatrix} 0.106 & 0.095 & 0.100 \\ 0.122 & 0.103 & 0.089 \\ 0.182 & 0.129 & 0.100 \end{bmatrix} \mathrm{W\,m^{-1}\,K^{-1}} \end{cases} \tag{S60}$$

$$\begin{cases} \left[T_{\mathrm{o},\{i,j\},\mathrm{re}}\right]_{3\times3} = \begin{bmatrix} 391 & 364 & 328 \\ 382 & 345 & 322 \\ 369 & 336 & 305 \end{bmatrix} \mathrm{K} \\ \left[\kappa_{\mathrm{l},\{i,j\}}\right]_{3\times3} = \begin{bmatrix} 0.320 & 1.191 & 0.554 \\ 0.123 & 0.102 & 0.196 \\ 0.016 & 0.016 & 0.017 \end{bmatrix} \mathrm{W\,m^{-1}\,K^{-1}} \qquad \text{Case 2,} \\ \left[\kappa_{\mathrm{r},\{i,j\}}\right]_{3\times3} = \begin{bmatrix} 0.115 & 0.091 & 0.100 \\ 0.144 & 0.191 & 0.100 \\ 0.217 & 0.190 & 0.100 \end{bmatrix} \mathrm{W\,m^{-1}\,K^{-1}} \end{cases} \tag{S61}$$

and

$$\begin{cases} \left[T_{\mathrm{o},\{i,j\},\mathrm{re}}\right]_{3\times3} = \begin{bmatrix} 390 & 365 & 333 \\ 385 & 352 & 315 \\ 372 & 345 & 309 \end{bmatrix} \mathrm{K} \\ \left[\kappa_{\mathrm{l},\{i,j\}}\right]_{3\times3} = \begin{bmatrix} 0.063 & 0.396 & 2.466 \\ 0.100 & 0.102 & 0.082 \\ 0.032 & 0.041 & 0.037 \end{bmatrix} \mathrm{W\,m^{-1}\,K^{-1}} \qquad \text{Case 3.} \\ \left[\kappa_{\mathrm{r},\{i,j\}}\right]_{3\times3} = \begin{bmatrix} 0.027 & 0.020 & 0.019 \\ 0.082 & 0.080 & 0.100 \\ 0.170 & 0.077 & 0.100 \end{bmatrix} \mathrm{W\,m^{-1}\,K^{-1}} \end{cases} \tag{S62}$$

Please refer to the corresponding finite element simulation results depicted in Supplementary Fig. 2c1-c3. For an in-depth insight, we extracted the maximum/average/minimum temperatures of the goods within $\mathrm{C}_{\{i,j\}}$ (Supplementary Fig. 2d).

Moreover, the number of $\mathrm{C}_{\{i,j\}}$ can be flexibly diminished or augmented based on specific demands. For clarity, diverse multi-temperature control systems with varying zones have been

conceptualized (as seen in Supplementary Fig. 2a2-a4, b2-b4)

$$\begin{cases} \left[T_{\mathrm{o},\{i,j\},\mathrm{re}}\right]_{1\times3} = \begin{bmatrix}381 & 349 & 312\end{bmatrix}\ \mathrm{K} \\ \left[\kappa_{\mathrm{l},\{i,j\}}\right]_{1\times3} = \begin{bmatrix}0.063 & 0.068 & 0.056\end{bmatrix}\ \mathrm{W\ m^{-1}\ K^{-1}} \\ \left[\kappa_{\mathrm{r},\{i,j\}}\right]_{1\times3} = \begin{bmatrix}0.084 & 0.076 & 0.100\end{bmatrix}\ \mathrm{W\ m^{-1}\ K^{-1}} \end{cases} \quad \text{Case A,} \tag{S63}$$

$$\begin{cases} \left[T_{\mathrm{o},\{i,j\},\mathrm{re}}\right]_{2\times2} = \begin{bmatrix}385 & 340 \\ 355 & 320\end{bmatrix}\ \mathrm{K} \\ \left[\kappa_{\mathrm{l},\{i,j\}}\right]_{2\times2} = \begin{bmatrix}0.255 & 0.388 \\ 0.044 & 0.066\end{bmatrix}\ \mathrm{W\ m^{-1}\ K^{-1}} \\ \left[\kappa_{\mathrm{r},\{i,j\}}\right]_{2\times2} = \begin{bmatrix}0.107 & 0.094 \\ 0.416 & 0.100\end{bmatrix}\ \mathrm{W\ m^{-1}\ K^{-1}} \end{cases} \quad \text{Case B,} \tag{S64}$$

and

$$\begin{cases} \left[T_{\mathrm{o},\{i,j\},\mathrm{r}}\right]_{3\times3-2} = \begin{bmatrix}393 & 359 & 328 \\ 384 & 343 & 321 \\ 371 & / & /\end{bmatrix}\ \mathrm{K} \\ \left[\kappa_{\mathrm{l},\{i,j\}}\right]_{3\times3-2} = \begin{bmatrix}0.416 & 0.378 & 0.554 \\ 0.132 & 0.089 & 0.172 \\ 0.025 & / & /\end{bmatrix}\ \mathrm{W\ m^{-1}\ K^{-1}} \\ \left[\kappa_{\mathrm{l},\{i,j\}}\right]_{3\times3-2} = \begin{bmatrix}0.106 & 0.109 & 0.100 \\ 0.121 & 0.220 & 0.100 \\ 0.010 & / & /\end{bmatrix}\ \mathrm{W\ m^{-1}\ K^{-1}} \end{cases} \quad \text{Case C.} \tag{S65}$$

Subsequent finite element simulations, showcased in Supplementary Fig. 2c4-c6, accentuate the multi-temperature control prowess of the systems. By comparing the data in Supplementary Fig. 2d with the multi-temperature prerequisites [Eqs. (S60-S65)], the alignment between the two sets of data demonstrates the universality of AMTC.

7 Meaning of the net heat flux q_N

For a 2-D multi-temperature control system (Fig. 4), we define the net heat flux q_N of each temperature control zone as

$$q_\mathrm{N} = q_\mathrm{L} - q_\mathrm{R} + q_\mathrm{D} - q_\mathrm{U}, \tag{S66}$$

where q_L, q_R, q_D, and q_U represent heat fluxes across the temperature control zone from the left, right, downward, and upward directions, respectively (inset in Fig. 4d). A value congruent with the assumed direction is taken as positive, while a contrasting one is negative. Simultaneously, the net heat flow of each temperature control zone is captured as:

$$Q_\mathrm{N} = Q_\mathrm{L} - Q_\mathrm{R} + Q_\mathrm{D} - Q_\mathrm{U}, \tag{S67}$$

with Q_L, Q_R, Q_D, and Q_U denoting heat flows in the left, right, down, up directions, respectively. Incorporating the relationship between the heat flux and heat flow, Eq. (S67) reads

$$Q_\mathrm{N} = q_\mathrm{L}\mathscr{L}_\mathrm{L} - q_\mathrm{R}\mathscr{L}_\mathrm{R} + q_\mathrm{D}\mathscr{L}_\mathrm{D} - q_\mathrm{U}\mathscr{L}_\mathrm{U}, \tag{S68}$$

where $\mathscr{L}_{\mathrm{L}}$, $\mathscr{L}_{\mathrm{R}}$, $\mathscr{L}_{\mathrm{D}}$, and $\mathscr{L}_{\mathrm{U}}$ are the lengths of the temperature control zone in the left, right, down, and up sides, respectively. For the system in Fig. 4a, $\mathscr{L}_{\mathrm{L}} = \mathscr{L}_{\mathrm{R}} = \mathscr{L}_{\mathrm{D}} = \mathscr{L}_{\mathrm{U}} = \tilde{\mathscr{L}}$. Then, Eq. (S68) reads

$$Q_{\mathrm{N}} = \tilde{\mathscr{L}} \left(q_{\mathrm{L}} - q_{\mathrm{R}} + q_{\mathrm{D}} - q_{\mathrm{U}} \right) = \tilde{\mathscr{L}} q_{\mathrm{N}}. \tag{S69}$$

Eq. (S69) illustrates a proportional relationship between q_{N} and Q_{N}. Since Q_{N} reflects the heat exchange intensity between the internal and external realms of the temperature control zone, employing q_{N} is equally effective for this purpose.

For a 3-D multi-temperature control system (Figs. 5, 7), we define the net heat flux q_{N} of each temperature control zone as

$$q_{\mathrm{N}} = q_{\mathrm{L}} - q_{\mathrm{R}} + q_{\mathrm{D}} - q_{\mathrm{U}} + q_{\mathrm{F}} - q_{\mathrm{B}}, \tag{S70}$$

where additional terms q_{F} and q_{B} denote the heat fluxes from the front and back sides of the temperature control zone, respectively (inset in Fig. 7b). Conversely, the net heat flow for each temperature control zone is formulated as:

$$\begin{aligned} Q_{\mathrm{N}} &= Q_{\mathrm{L}} - Q_{\mathrm{R}} + Q_{\mathrm{D}} - Q_{\mathrm{U}} + Q_{\mathrm{F}} - Q_{\mathrm{B}} \\ &= q_{\mathrm{L}} A_{\mathrm{L}} - q_{\mathrm{R}} A_{\mathrm{R}} + q_{\mathrm{D}} A_{\mathrm{D}} - q_{\mathrm{U}} A_{\mathrm{U}} + q_{\mathrm{F}} A_{\mathrm{F}} - q_{\mathrm{B}} A_{\mathrm{B}}, \end{aligned} \tag{S71}$$

where Q_{F} and Q_{B} are the heat flows across the temperature control zone in the front and back directions, respectively;A_{L}, A_{R}, A_{D}, A_{U}, A_{F} and A_{B} are the areas of the temperature control zone in the left, right, down, up, front and back directions, respectively. Considering the system delineated in Fig. 5b, c, $A_{\mathrm{L}} = A_{\mathrm{R}} = A_{\mathrm{F}} = A_{\mathrm{B}} = \tilde{A}$ and $A_{\mathrm{U}} = A_{\mathrm{D}} = \hat{A}$, Eq. (S71) can be further condensed to:

$$Q_{\mathrm{N}} = \tilde{A} \left(q_{\mathrm{L}} - q_{\mathrm{R}} + q_{\mathrm{F}} - q_{\mathrm{B}} \right) + \hat{A} \left(q_{\mathrm{D}} - q_{\mathrm{U}} \right). \tag{S72}$$

By integrating Eq. (S70) and Eq. (S72), the derived equation is

$$\begin{aligned} Q_{\mathrm{N}} &= \tilde{A} \left[q_{\mathrm{N}} - \left(q_{\mathrm{D}} - q_{\mathrm{U}} \right) \right] + \hat{A} \left(q_{\mathrm{D}} - q_{\mathrm{U}} \right) \\ &= \tilde{A} q_{\mathrm{N}} + (\hat{A} - \tilde{A}) \left(q_{\mathrm{D}} - q_{\mathrm{U}} \right), \end{aligned} \tag{S73}$$

leading to the expression for net heat flux

$$q_{\mathrm{N}} = \frac{Q_{\mathrm{N}} - (\hat{A} - \tilde{A}) \left(q_{\mathrm{D}} - q_{\mathrm{U}} \right)}{\tilde{A}}. \tag{S74}$$

Given the robust thermal insulation of the upper and lower facets of the temperature control zone, both q_{D} and q_{U} tend to be diminutive. Furthermore, as $\hat{A}$ approximates $\tilde{A}$, the differential $(\hat{A} - \tilde{A})$ remains small. Thus, the term $(\hat{A} - \tilde{A}) \left(q_{\mathrm{D}} - q_{\mathrm{U}} \right) / \tilde{A} \to 0$. The simplified version of Eq. (S74) can be treated as

$$q_{\mathrm{N}} \to \frac{Q_{\mathrm{N}}}{\tilde{A}}. \tag{S75}$$

Mirroring the earlier conclusion, q_{N} aptly depicts the overall heat exchange intensity between the inner and outer realms of the temperature control zone in real-time.

8 Characteristics and mechanisms of AMTC.

We further analyze the system in Fig. 4a. For comprehensive understanding, we employed a normalized temperature expression for universality. As outlined in the manuscript, the required normalized temperature, denoted as Ψ, for each designated zone, represented by γ, is given by

$$[\Psi_{\gamma,\mathrm{re}}]_{3\times3} = \begin{bmatrix} \Psi_{①} & \Psi_{④} & \Psi_{⑦} \\ \Psi_{②} & \Psi_{⑤} & \Psi_{⑧} \\ \Psi_{③} & \Psi_{⑥} & \Psi_{⑨} \end{bmatrix} = \begin{bmatrix} 0.9 & 0.6 & 0.3 \\ 0.8 & 0.5 & 0.2 \\ 0.7 & 0.4 & 0.1 \end{bmatrix}. \tag{S76}$$

To cater to the temperature needs of the goods, we organized them such that their temperature requirements align with the elements presented in Eq. (S76). In this arrangement, goods were placed in their corresponding temperature control zones. Supplementary Fig. 5a depicts a scenario where goods entirely occupy storage spaces. The normalized temperature of the heat (cold) source was set at 1.0 (0.0) or equivalently, 400 K (300 K). Using the AMTC principle, we discerned the thermal conductivities for both blue and red zones (detailed in Methods, Supplementary Note 4). Subsequent steady and transient finite element simulations were then conducted.

From a steady-state perspective, the normalized temperatures of the goods aligned with the elements in Eq. (S76), as showcased in Supplementary Fig. 5b, c. The normalized temperature distributions along the marked characteristic lines in Supplementary Fig. 5b revealed a terraced pattern, consistent with the idea in Fig. 1c, d (Supplementary Fig. 5d). An apt design, satisfying the temperature uniformity condition (referenced in Supplementary Note 1.3), ensures that the multi-temperature control's efficacy is largely unaffected by variances in goods' thermal conductivities, phases, proportions, shapes, and quantities (see Supplementary Note 9). Hence, even in the absence of goods within the storage space (with the void filled by air), the terraced normalized temperature distributions remain fairly stable (Supplementary Fig. 5d-f). This flexibility in placement or removal of goods from storage areas augments its practical utility. Further investigations suggest that enhancing the thermal conductivities of packaging structures can elevate the effective thermal conductivities of temperature control zones, thereby bolstering the precision of AMTC (delved deeper in Supplementary Note 10). By selecting materials with optimal thermal conductivities for crafting the package structure, the quality of transported goods is ensured.

Transitioning to a transient analysis, we set the initial normalized temperatures of goods (as in Supplementary Fig. 5a) to 0.5 (Methods). The system will increase, reduce, and maintain the goods's temperature according to each zone's preset normalized temperature [Eq. (S76)] and the goods's initial normalized temperature (Supplementary Fig. 5g). Upon all goods reaching their predetermined normalized temperature, the system stabilizes, underscoring its multi-temperature maintenance capability. To unravel the underlying mechanism of AMTC, we graphed the temporal evolution of heat fluxes across each temperature control zone. A defined net heat flux encapsulated the cumulative effect of these fluxes (see Supplementary Fig. 5h and Supplementary Note 7). It mirrors the intensity of heat exchange between the inner and outer regions of the temperature control zone. Supplementary Fig. 5i highlights the consistent patterns between transient net heat fluxes across each zone and temperature variations in the corresponding goods. A more pronounced deviation between a good's normalized temperature

and its preset value results in a more significant net heat flux. Intriguingly, when the system reaches a steady state, the heat fluxes across the temperature control zones, from both right and left directions, balance dynamically. Their cumulative impact on net heat flux nears zero, implying that temperature interactions among differently-tempered goods play a pivotal role in multi-temperature control. This validates our claim of achieving multi-temperature control while harnessing temperature interactions. Additionally, a factor, denoted as β, to represent the thermal diffusivity of the system components (Methods). A higher β signifies increased thermal diffusivity. Analyzing the influence of β on the transient multi-temperature control process, we ascertain that a system with a heightened β can more rapidly align the goods' temperature to their requisite values (Supplementary Fig. 5j). Thus, amplifying the thermal diffusivities of system components can bolster the system's multi-temperature regulation rate amidst temperature interactions. This trait proves invaluable in countering temperature fluctuations of transported goods in initial stages. Specifically, it's essential that all goods are pre-cooled (or pre-heated) to their required temperatures prior to transportation[1,2]. Nonetheless, the multi-temperature control system's initial temperature may deviate from that of the goods (Fig. 4a). Post-placement of goods into the system, their temperature might experience perturbations. The rapid temperature regulation attribute of AMTC proves beneficial in rectifying these temperature fluctuations, ensuring consistent multi-temperature maintenance.

9 Influence of the thermal conductivities, phases, proportions (area/volume fractions), shapes, and numbers of the goods in the temperature control zones on the multi-temperature control performance

To ensure the multi-temperature control performance of the multi-temperature control system, we introduced the temperature-uniform condition (Supplementary Note 1.3). Under this condition, the multi-temperature control performance of the multi-temperature control system is not influenced by the thermal/structural parameters of $\mathrm{C}_{\{m,j\}}$. As a result, we can store goods with different thermal conductivities and structures in $\mathrm{C}_{\{i,j\}}$ and maintain them at the required temperatures. Nonetheless, practical applications will require a more detailed examination.

In Fig. 3a-c, for simplicity, we assume that $\kappa_{\mathrm{c},\{i,j\}} = \kappa_{\mathrm{o},\{i,j\}} = \kappa_{\mathrm{pac},\{i,j\}} = 1\ \mathrm{W\ m^{-1}\ K^{-1}}$ (Methods). When contrasted with $\kappa_{\mathrm{l},\{i,j\}}$ and $\kappa_{\mathrm{r},\{i,j\}}$, it becomes evident that the temperature-uniform condition is upheld, ensuring precise multi-temperature control performance. However, how would $T_{\mathrm{o},\{i,j\}}$ be affected if we modify $\kappa_{\mathrm{o},\{i,j\}}$? Our aim is to achieve multi-temperature control for a broader range of goods with different thermal conductivities using this system. The lowest known thermal conductivity magnitude order for commercial materials is approximately $10^{-3}\ \mathrm{W\ m^{-1}\ K^{-1}}$ [3,4]. If $\kappa_{\mathrm{o},\{i,j\}}$ approaches $10^{-3}\ \mathrm{W\ m^{-1}\ K^{-1}}$, it becomes challenging to locate materials that strictly adhere to the temperature-uniform condition, potentially compromising the system's multi-temperature control capabilities. Additionally, our ambition extends to controlling temperatures for goods with varying phases, proportions (in terms of area fractions/volume fractions), shapes, and quantities, to enhance the versatility of the multi-temperature control system. Consequently, we will be analyzing the impact of these factors on

the system's performance.

9.1 Influence of thermal conductivities

Based on the multi-temperature control system depicted in Supplementary Fig. 2a, we configure $\left[\kappa_{\mathrm{o},\{i,j\}}\right]_{3\times3}$ to a range of values: $[0.001]_{3\times3}$, $[0.01]_{3\times3}$, $[0.05]_{3\times3}$, $[0.1]_{3\times3}$, $[0.2]_{3\times3}$, $[0.5]_{3\times3}$, $[1]_{3\times3}$, $[5]_{3\times3}$, $[10]_{3\times3}$, $[100]_{3\times3}$, and $[1000]_{3\times3}$ $\mathrm{W\ m^{-1}\ K^{-1}}$. All other parameters remain consistent with the original ones. We then examine the influence of $\kappa_{\mathrm{o},\{i,j\}}$ on the multi-temperature control performance by finite element simulations.

We present temperature distributions for two scenarios: $\left[\kappa_{\mathrm{o},\{i,j\}}\right]_{3\times3} = [0.001]_{3\times3}$ $\mathrm{W\ m^{-1}\ K^{-1}}$ and $\left[\kappa_{\mathrm{o},\{i,j\}}\right]_{3\times3} = [1000]_{3\times3}$ $\mathrm{W\ m^{-1}\ K^{-1}}$ (Supplementary Fig. 6a1, a2). Further insights into the maximum/average/minimum temperatures of the goods in $\mathrm{C}_{\{i,j\}}$ can be found in Supplementary Fig. 6a3. When the temperature-uniform condition is strictly met, changes in $\kappa_{\mathrm{o},\{i,j\}}$ have not impact on $T_{\mathrm{o},\{i,j\}}$. Consequently, in practical applications, it is advisable for the thermal conductivities of actual materials used in $\mathrm{L}_{\{i,j\}}$ & $\mathrm{R}_{\{i,j\}}$ to be as low as possible.

However, as previously mentioned, the lowest thermal conductivity for commercial (insulating) materials generally falls around 10^{-3} $\mathrm{W\ m^{-1}\ K^{-1}}$. When $\kappa_{\mathrm{o},\{i,j\}}$ approximates this value, strict adherence to the temperature-uniform condition might be compromised, resulting in temperature deviations between simulation outcomes and the desired values (Supplementary Fig. 6a3). Fortunately, these deviations are relatively minor, making them tolerable when stringent requirements for multi-temperature control are not paramount.

Our ultimate aim is to attain flawless multi-temperature control performance from a design standpoint. To achieve this, we propose several solutions for scenarios where $\kappa_{\mathrm{o},\{i,j\}}$ reaches low levels in subsequent discussions.

9.2 Influence of phases

In the "Principle of AMTC," our study primarily centers on a conduction heat transfer system for the sake of simplicity. Yet, in practical applications, we anticipate the need to store goods across different phases. For those goods in fluid states, the inherent convective heat transfer can be effectively equated to an enhancement in $\kappa_{\mathrm{o},\{i,j\}}$ $\left(\kappa_{\mathrm{c},\{i,j\},\mathrm{eff}}\right)$, thereby augmenting the multi-temperature performance (Supplementary Note 9.1). Thus, the multi-temperature control system is aptly suited for storing goods in fluid states.

Delving deeper, the convective heat transfer intensity in the fluid is positively correlated with the Nu number. When dealing with weak convective heat transfer (taking Nu=1 as an example), the negative impact of a low $\kappa_{\mathrm{o},\{i,j\}}$ on multi-temperature control performance are not fully mitigated (as illustrated in Supplementary Fig. 6b1-b3). This implies that for achieving optimal multi-temperature control, the thermal conductivity of goods in fluid states should not be overly low. However, from a design viewpoint, imposing specifications on the thermal conductivities of goods may seem counterintuitive. In such scenarios, elevating the Nu number could be a viable solution. By introducing a rotor into the liquid to induce internal flow, pronounced convective heat transfer can be achieved. The intensity of this flow is directly proportional to the Nu number. Such a mechanism can substantially amplify the heat transfer within the

fluid, which can be likened to a boost in $\kappa_{\mathrm{o},\{i,j\}}$ $\left(\kappa_{\mathrm{c},\{i,j\},\mathrm{eff}}\right)$, favorably impacting the multi-temperature control performance (Supplementary Fig. 6c1-c3, Solution 1).

9.3 Influence of area fractions

The area fraction of the goods in $\mathrm{C}_{\{i,j\}}$ $\left(f_{\mathrm{o},\{i,j\}}\right)$ pivotal to the multi-temperature control performance, especially when $\kappa_{\mathrm{o},\{i,j\}}$ is low. We executed finite element simulations, treating the area fractions as variables (refer to Supplementary Figs. 7-9). A decline in $f_{\mathrm{o},\{i,j\}}$ results in a corresponding rise in $f_{\mathrm{pac},\{i,j\}}$. Consequently, the influence of the packaging structure on the effective thermal conductivity of $\mathrm{C}_{\{i,j\}}$ amplifies. Therefore, when $\kappa_{\mathrm{o},\{i,j\}}$ is low, diminishing $f_{\mathrm{o},\{i,j\}}$ emerges as a viable strategy to enhance the multi-temperature control performance (Solution 2).

9.4 Influence of shapes and numbers

When the temperature-uniform condition is met, the shapes (whether circle, square, or triangle) and quantities of goods do not affect the multi-temperature control performance (Supplementary Figs.7-10). However, if $\kappa_{\mathrm{o},\{i,j\}}$ is low, reducing the number of goods proves advantageous for enhancing the multi-temperature control performance (Supplementary Fig. 10f1).

9.5 Summary and discussion

Our analysis underscores that, when the temperature-uniform condition is met, $T_{\mathrm{o},\{i,j\}}$ can be rigorously regulated as per the stipulated requirements. In such instances, the multi-temperature control performance of the system remains independent of the thermal conductivities, phases, area fractions, shapes, and quantities of the goods.

To ensure a comprehensive assessment, we also contemplate an extreme scenario wherein $\kappa_{\mathrm{o},\{i,j\}}$ is quite low. Through an exhaustive analysis complemented by corresponding simulations, we discern that elevating the Nu number for goods in fluid states and minimizing the area fraction (or number) of goods are conducive for multi-temperature performance. However, both methods possess certain limitations pertaining to the nature of the goods; one mandates that the goods be in a fluid state, while the other necessitates a decrease in $f_{\mathrm{o},\{i,j\}}$. This is not ideal for the multi-temperature control system. So, what alternative strategies might we deploy to address this challenge? As per the effective medium theory, the effective thermal conductivity of $\mathrm{C}_{\{i,j\}}$ $\left(\kappa_{\mathrm{c},\{i,j\},\mathrm{eff}}\right)$ hinges upon $\kappa_{\mathrm{o},\{i,j\}}$, $\kappa_{\mathrm{pac},\{i,j\}}$, $f_{\mathrm{o},\{i,j\}}$, and $f_{\mathrm{pac},\{i,j\}}$. In instances where $\kappa_{\mathrm{o},\{i,j\}}$ is low there is a desire to retain a high $f_{\mathrm{o},\{i,j\}}$, amplifying $\kappa_{\mathrm{pac},\{i,j\}}$ might emerge as a viable solution. To validate this hypothesis, we embarked on associated finite element simulations. We set $\left[\kappa_{\mathrm{pac},\{i,j\}}\right]_{3\times3}$ to $[10]_{3\times3}$ and $[1000]_{3\times3}$ $\mathrm{W\ m^{-1}\ K^{-1}}$, while other thermal/structural parameters mirror the original settings. Subsequently, we evaluated the influence of $\kappa_{\mathrm{pac},\{i,j\}}$ on the multi-temperature control performance based on Supplementary Note 9.4 (Supplementary Fig. 10c1-c3, e1-e3, f1, f3). The multi-temperature control system's performance enhances with an increment in $\kappa_{\mathrm{pac},\{i,j\}}$. Since we possess the flexibility to select materials to design the package structure in practical scenarios, this strategy boasts broad adaptability. Here are the consolidated strategies to boost the multi-temperature control performance:

- Elevate the Nu of the goods in fluid states to effectively augment $\kappa_{\mathrm{o},\{i,j\}}$.
- Trim down the proportions or quantity of the goods in $\mathrm{C}_{\{i,j\}}$ to minimize $f_{\mathrm{o},\{i,j\}}$.
- Opt for a package structure with superior thermal conductivity to elevate $\kappa_{\mathrm{pac},\{i,j\}}$.

In essence, AMTC enables precise multi-temperature control performance for a plethora of goods varying in thermal conductivities, phases, area fractions, shapes, and quantities in 2-D scenarios. Consequently, we surmise that this conclusion also holds true for 3-D contexts. We then crafted a 3-D multi-temperature control system to accommodate diverse goods in $\mathrm{C}_{\{i,j\}}$ (Supplementary Fig. 11). The meticulous multi-temperature control performance suggests the system's suitability for practical applications.

Further, we consider a more pragmatic scenario where air is introduced above the goods, representing areas where temperature control zones are not densely populated with goods and package structures (Supplementary Fig. 12). The steady-state simulation indicates that the presence of air does not sway the multi-temperature control performance. For transient simulations, the air's low density and specific heat capacity even expedite the temperature modulation time of the system.

These scenarios substantiate that the multi-temperature control system, crafted using our methodologies, is adept for 3-D environments.

10 Calculation of effective thermal conductivity for the temperature control zone

The effective medium approximation[5–7] can help us to calculate $\kappa_{\mathrm{c},\{i,j\},\mathrm{eff}}$. For simplicity, we explain it by a 2-D Maxwell-Garnett approximation

$$\frac{\kappa_{\mathrm{c},\{i,j\},\mathrm{eff}} - \kappa_{\mathrm{pac},\{i,j\}}}{\kappa_{\mathrm{c},\{i,j\},\mathrm{eff}} + \kappa_{\mathrm{pac},\{i,j\}}} = f_{\mathrm{o},\{i,j\}} \frac{\kappa_{\mathrm{o},\{i,j\}} - \kappa_{\mathrm{pac},\{i,j\}}}{\kappa_{\mathrm{o},\{i,j\}} + \kappa_{\mathrm{pac},\{i,j\}}}, \tag{S77}$$

and a 2-D Bruggeman approximation

$$f_{\mathrm{o},\{i,j\}} \frac{\kappa_{\mathrm{o},\{i,j\}} - \kappa_{\mathrm{c},\{i,j\},\mathrm{eff}}}{\kappa_{\mathrm{o},\{i,j\}} + \kappa_{\mathrm{c},\{i,j\},\mathrm{eff}}} + f_{\mathrm{pac},\{i,j\}} \frac{\kappa_{\mathrm{pac},\{i,j\}} - \kappa_{\mathrm{c},\{i,j\},\mathrm{eff}}}{\kappa_{\mathrm{pac},\{i,j\}} + \kappa_{\mathrm{c},\{i,j\},\mathrm{eff}}} = 0. \tag{S78}$$

Setting different combinations of $\kappa_{\mathrm{o},\{i,j\}}$ and $\kappa_{\mathrm{pac},\{i,j\}}$, we get a relationship between $\kappa_{\mathrm{c},\{i,j\},\mathrm{eff}}$ and $f_{\mathrm{o},\{i,j\}}$ (Supplementary Fig. 13). Case 1 studies the situation where the thermal conductivities of the storing goods are low. It shows that reducing $f_{\mathrm{o},\{i,j\}}$ is an effective way to increase $\kappa_{\mathrm{c},\{i,j\},\mathrm{eff}}$. Case 2 is ideal where $\kappa_{\mathrm{o},\{i,j\}}$ and $\kappa_{\mathrm{pac},\{i,j\}}$ are significantly higher than $\kappa_{\mathrm{l},\{i,j\}}$ and $\kappa_{\mathrm{r},\{i,j\}}$. The $\kappa_{\mathrm{c},\{i,j\},\mathrm{eff}}$ is obviously at a high value, which fully satisfies the temperature-uniform condition. Case 3 indicates that increasing $\kappa_{\mathrm{pac},\{i,j\}}$ can increase $\kappa_{\mathrm{c},\{i,j\},\mathrm{eff}}$ when $\kappa_{\mathrm{o},\{i,j\}}$ is low. The above results agree with the finite element simulations in Supplementary Note 9. Therefore, these two approximations might guide the design of the multi-temperature control system.

In addition, the $\kappa_{\mathrm{c},\{i,j\},\mathrm{eff}}$ calculated by the above two approximations are different. For instance, in case 3, when $f_{\mathrm{o},\{i,j\}} > 50\%$, $\kappa_{\mathrm{c},\{i,j\},\mathrm{eff}}$ calculated by Maxwell-Garnett approximation is higher than the Bruggeman approximation. It depends on the specific arrangement of

the goods and the package structures in $\mathrm{C}_{\{i,j\}}$. Regarding the multi-temperature control system discussed in this work, the Maxwell-Garnett approximation is more suitable for calculating $\kappa_{\mathrm{c},\{i,j\},\mathrm{eff}}$ than Bruggeman approximation. The heat flow can traverse $\mathrm{C}_{\{i,j\}}$ along with the high thermal conductivity package structure, which keeps $\kappa_{\mathrm{c},\{i,j\},\mathrm{eff}}$ at a high value. This discussion implies that when we place goods in $\mathrm{C}_{\{i,j\}}$, we should not break the connection of the package structure. It is beneficial for multi-temperature control performance.

11 Design method of the multi-temperature maintenance container

11.1 Extended theory for an actual 2-D multi-temperature control system

For practical applications, we turn our focus to a multi-temperature control system comprised of five distinct zones $\left(\mathrm{M}_{\{i,j\}},\ \mathrm{L}_{\{i,j\}},\ \mathrm{C}_{\{i,j\}},\ \mathrm{R}_{\{i,j\}},\ \text{and S}\right)$, designed for a multi-temperature maintenance container, as delineated in Supplementary Fig. 17a. In contrast to Fig. 2, the system has integrated zones $\mathrm{M}_{\{i,j\}}$. For ease in real-world applications, these zones are filled with low thermal conductivity materials.

With the incorporation of $\mathrm{M}_{\{i,j\}}$, the foundational theory for AMTC warrants an expansion. Taking the instance where n is odd $[n=\{1,3,5\cdots 2h+1\}\ (h=0,1,2,3\cdots)]$. We combine $\mathrm{M}_{\{i,j\}}$ and its closest neighbor $\mathrm{L}_{\{i,j\}}$ $\left(\mathrm{R}_{\{i,j\}}\right)$ to formulate $\mathrm{L}'_{\{i,j\}}$ $\left(\mathrm{R}'_{\{i,j\}}\right)$, as illustrated in Supplementary Fig. 17b. This modification ensures the continued viability of our original methodology.

Establishing the relationships in thermal/structural parameters between $\mathrm{L}_{\{i,j\}}$ and $\mathrm{L}'_{\{i,j\}}$ $\left(\mathrm{R}_{\{i,j\}}\text{ and }\mathrm{R}'_{\{i,j\}}\right)$, we have

$$\frac{d_{\mathrm{m},\{i,j\}}}{\kappa_{\mathrm{m},\{i,j\}}}+\frac{d_{\mathrm{l},\{i,j\}}}{\kappa_{\mathrm{l},\{i,j\}}}=\frac{d_{\mathrm{m},\{i,j\}}+d_{\mathrm{l},\{i,j\}}}{\kappa_{\mathrm{l}',\{i,j\}}}=\frac{d_{\mathrm{l}',\{i,j\}}}{\kappa_{\mathrm{l}',\{i,j\}}} \tag{S79}$$

and

$$\frac{d_{\mathrm{m},\{i,j\}}}{\kappa_{\mathrm{m},\{i,j\}}}+\frac{d_{\mathrm{r},\{i,j\}}}{\kappa_{\mathrm{r},\{i,j\}}}=\frac{d_{\mathrm{m},\{i,j\}}+d_{\mathrm{r},\{i,j\}}}{\kappa_{\mathrm{r}',\{i,j\}}}=\frac{d_{\mathrm{r}',\{i,j\}}}{\kappa_{\mathrm{r}',\{i,j\}}}, \tag{S80}$$

where $\kappa_{\mathrm{l}',\{i,j\}}$, $\kappa_{\mathrm{r}',\{i,j\}}$, and $\kappa_{\mathrm{m},\{i,j\}}$ are thermal conductivities of $\mathrm{L}'_{\{i,j\}}$, $\mathrm{R}'_{\{i,j\}}$, and $\mathrm{M}_{\{i,j\}}$, respectively. $d_{\mathrm{l}',\{i,j\}}$, $d_{\mathrm{r}',\{i,j\}}$, and $d_{\mathrm{m},\{i,j\}}$ are the length traversed by heat flow of $\mathrm{L}'_{\{i,j\}}$, $\mathrm{R}'_{\{i,j\}}$, and $\mathrm{M}_{\{i,j\}}$, respectively. Following the temperature-uniform condition, the Eq. (S21) and Eq. (S33) can be rewritten as

$$\begin{cases}\theta_{\mathrm{c},\{m,j\}}\big|_{j=j_\mathrm{a}}=\left(\dfrac{d_{\mathrm{r}',\{m,j_\mathrm{a}\}}}{\kappa_{\mathrm{r}',\{m,j_\mathrm{a}\}}}+\dfrac{d_{\mathrm{c},\{m,j_\mathrm{a}\}}/2}{\kappa_{\mathrm{c},\{m,j_\mathrm{a}\}}}\right)\Big/\displaystyle\sum_{\epsilon'=\mathrm{l}',\mathrm{c},\mathrm{r}'}\dfrac{d_{\epsilon',\{m,j_\mathrm{a}\}}}{\kappa_{\epsilon',\{m,j_\mathrm{a}\}}}\\ \theta_{\mathrm{c},\{m,j\}}\big|_{j=j_\mathrm{b}}=\left(\dfrac{d_{\mathrm{r},\{m,j_\mathrm{b}\}}}{\kappa_{\mathrm{r},\{m,j_\mathrm{b}\}}}+\dfrac{d_{\mathrm{c},\{m,j_\mathrm{b}\}}/2}{\kappa_{\mathrm{c},\{m,j_\mathrm{b}\}}}\right)\Big/\displaystyle\sum_{\epsilon=\mathrm{l},\mathrm{c},\mathrm{r}}\dfrac{d_{\epsilon,\{m,j_\mathrm{b}\}}}{\kappa_{\epsilon,\{m,j_\mathrm{b}\}}}\end{cases}, \tag{S81}$$

and

$$\begin{cases} \Lambda_{\{m,j\}}\big|_{j=j_\mathrm{a}} = \dfrac{\sum_{\epsilon'=\mathrm{l}',\mathrm{c},\mathrm{r}'} d_{\epsilon',\{m,j_\mathrm{a}\}}/\kappa_{\epsilon',\{m,j_\mathrm{a}\}}}{\sum_{\epsilon'=\mathrm{l}',\mathrm{c},\mathrm{r}'}\sum_{j'_\mathrm{a}=1}^{2h+1} d_{\epsilon',\{m,j'_\mathrm{a}\}}/\kappa_{\epsilon',\{m,j'_\mathrm{a}\}} + \sum_{\epsilon=\mathrm{l},\mathrm{c},\mathrm{r}}\sum_{j'_\mathrm{b}=2}^{2h} d_{\epsilon,\{m,j'_\mathrm{b}\}}/\kappa_{\epsilon,\{m,j'_\mathrm{b}\}}} \\ \Lambda_{\{m,j\}}\big|_{j=j_\mathrm{b}} = \dfrac{\sum_{\epsilon=\mathrm{l},\mathrm{c},\mathrm{r}} d_{\epsilon,\{m,j_\mathrm{b}\}}/\kappa_{\epsilon,\{m,j_\mathrm{b}\}}}{\sum_{\epsilon'=\mathrm{l}',\mathrm{c},\mathrm{r}'}\sum_{j'_\mathrm{a}=1}^{2h+1} d_{\epsilon',\{m,j'_\mathrm{a}\}}/\kappa_{\epsilon',\{m,j'_\mathrm{a}\}} + \sum_{\epsilon=\mathrm{l},\mathrm{c},\mathrm{r}}\sum_{j'_\mathrm{b}=2}^{2h} d_{\epsilon,\{m,j'_\mathrm{b}\}}/\kappa_{\epsilon,\{m,j'_\mathrm{b}\}}} \end{cases}, \tag{S82}$$

where $j_\mathrm{a}(j'_\mathrm{a}) = \{1, 3, 5, \cdots, 2h+1\}$ and $j_\mathrm{b}(j'_\mathrm{b}) = \{2, 4, 6, \cdots, 2h\}$ ($n = 2h+1$, $h = 0, 1, 2, \cdots$)[1]; ϵ' denotes zone E' ($E' = \{\mathrm{L}', \mathrm{C}, \mathrm{R}'\}$). $\epsilon' = \mathrm{l}'$, $\epsilon' = \mathrm{c}$, and $\epsilon' = \mathrm{r}'$ represent $E' = \mathrm{L}'$, $E' = \mathrm{C}$, and $E' = \mathrm{R}'$, respectively; $\kappa_{\epsilon'}$ is thermal conductivity of E'; $d_{\epsilon'}$ is the length traversed by heat flow of E'. Following the core idea of the approaches in Supplementary Note 5, all the thermal/structural parameters of the multi-temperature control system can be obtained.

11.2 Selecting two kinds of high-performance phase change materials (PCMs) with different phase transition temperatures

The manuscript outlines the fundamental requirements for PCMs. Here, we delve into more details. For PCM A, its solidification process allows it to function as a heat source. As such, the solidification temperature of PCM A must remain stable. After evaluating the thermal properties of various PCMs, including inorganic PCM, organic PCM, and eutectic PCM, we opted for stearic acid (an organic PCM type). This choice is motivated by its suitable phase transition temperature[8] and the benefits it offers, such as negligible supercooling, absence of phase separation, and cost-effectiveness[9–11]. As for PCM B, its role as a cold source is realized through its melting process. Given that the melting processes of PCMs are typically stable, we selected distilled water, favoring its high latent heat. The PCM selection strategy discussed above can be used as a reference for real-world applications. Future developments in PCM, such as enhancing heat transfer, minimizing supercooling, and boosting thermal and chemical stability, are certainly advantageous[11–20].

11.3 Selecting actual materials for the multi-temperature control system

In line with Supplementary Note 11.1, we select actual materials for a multi-temperature control system comprising 3×3 zones. Given the known structural parameters of the glassware, we can base our system on a suitable-sized commercial thermal insulating container (provided by Tongling Zhongpai Electronic Commerce Co., Ltd., China) of a suitable size as a basis. Leaving appropriate space for the mobile heat/cold source, the rest is allocated to the system (Supplementary Fig. 18, step 1).

The key challenge here is to design the system using general commercial homogeneous isotropic materials. Utilizing vacuum insulation plates, a commonly used low thermal conductivity material (thermal conductivity: 0.008 W m^{-1} K^{-1}), we filled zone S. Ceramic fiber papers (thermal conductivity: 0.080 W m^{-1} K^{-1}) of specific dimensions were chosen for $\mathrm{M}_{\{i,j\}}$.

[1] If n is even, $j_\mathrm{b}(j'_\mathrm{b}) = \{2, 4, 6, \cdots, 2h+2\}$ ($n = 2h+2$).

With these in place, the structural parameters for the remaining materials were ascertained (Supplementary Fig. 18, step 2). All the structural parameters of the system were then obtained

$$\begin{cases} \left[d_{\mathrm{m},\{i,j\}}\right]_{3\times3} = [10]_{3\times3}\ \mathrm{mm} \\ \left[d_{\mathrm{l},\{i,j\}}\right]_{3\times3} = [10]_{3\times3}\ \mathrm{mm} \\ \left[d_{\mathrm{r},\{i,j\}}\right]_{3\times3} = [10]_{3\times3}\ \mathrm{mm} \end{cases}. \tag{S83}$$

Assuming that the temperature of the heat (cold) source T_{A} (T_{B}) is consistent with the phase transition temperature of PCM A (PCM B), we derived

$$\begin{cases} T_{\mathrm{A}} = 340.15\ \mathrm{K} \\ T_{\mathrm{B}} = 273.15\ \mathrm{K} \end{cases}. \tag{S84}$$

Following the core concepts laid out in Supplementary Notes 5 and 11.1, our final thermal conductivities are

$$\left[\kappa_{\mathrm{l},\{i,j\}}\right]_{3\times3} = \left[\kappa_{\mathrm{r},\{i,j\}}\right]_{3\times3} = \begin{bmatrix} 0.020 & 0.026 & 0.011 \\ 0.010 & 0.018 & 0.010 \\ 0.008 & 0.019 & 0.014 \end{bmatrix}\ \mathrm{W\ m^{-1}\ K^{-1}}. \tag{S85}$$

Given the equation

$$\begin{cases} \dfrac{d_{\epsilon,\{i,j\}}}{\kappa_{\epsilon,\{i,j\}}} = \displaystyle\sum_{k=1}^{p} \dfrac{d_{\epsilon,\{i,j\},k}}{\kappa_{\epsilon,\{i,j\},k}} \\ d_{\epsilon,\{i,j\}} = \displaystyle\sum_{k=1}^{p} d_{\epsilon,\{i,j\},k} \end{cases}, \tag{S86}$$

where $\kappa_{\epsilon,\{i,j\},k}$ and $d_{\epsilon,\{i,j\},k}$ represent the thermal conductivity and the length traversed by heat flow of actual material k ($k = \{1, 2, 3\cdots p\}$, p is the number of the actual materials), we selected the actual materials for the system's remaining parts (Supplementary Fig. 18, step 3). Thermal and structural parameters of these materials are detailed in Supplementary Tables 3-4.

A steady-state simulation was subsequently conducted to validate our design (Supplementary Fig. 19). The error between the simulation (experimental) result $\left[\Omega_{\mathrm{si(ex)}}\right]$ and the requisite value Ω_{re} was calculated using

$$Err = \frac{\Omega_{\mathrm{si(ex)}} - \Omega_{\mathrm{re}}}{\Omega_{\mathrm{re}}} \times 100\%. \tag{S87}$$

The minimal errors, ranging from -1.34% to 1.30%, indicate that our multi-temperature control system, founded on the method described, can be feasibly realized (Supplementary Fig. 19c).

11.4 Multi-temperature maintenance test: a finite element simulation

We employed finite element simulation to assess the performance of the multi-temperature maintenance container. We constructed a 2-D rendition of this container (Fig. 5c). For comparison, a standard 2-D commercial thermal insulating container was utilized as a control group. Subsequent finite element simulations were executed (Supplementary Fig. 20).

The findings illustrate that the rate of temperature variations for goods stored in the multi-temperature maintenance container is considerably lower than those in the commercial thermal insulating container (Supplementary Fig. 20a1, a2). This suggests that the multi-temperature maintenance performance of our specialized container outperforms the commercial variant. These results signal that a practical multi-temperature maintenance container can be crafted using the aforementioned procedure. Complementing the discourse on the "Multi-temperature maintenance container based on AMTC" within the main manuscript, we have also presented 3-D schematics of the container both with and without AMTC for real-world applications (Supplementary Fig. 21).

Moreover, the distribution of temperature and phase across the containers unravels the mechanism underpinning the multi-temperature maintenance abilities of our container. When the left/right aspects of the multi-temperature control system are sustained at their desired temperatures (340.15 K/273.15 K), the multi-temperature maintenance container achieves exceptional performance for an extended period (roughly 0-40 hours). As a result, any technological advancements that prolong the maintenance of the left/right side temperatures can enhance the multi-temperature maintenance capabilities. Potential avenues include increasing the energy density of the PCMs or managing the phase change interface to remain proximate to the left/right side[13, 14].

12 Comparison of a multi-temperature maintenance container with a commercial thermal insulating container and a phase change thermal insulating container

Within the scope of this work, given that the AMTC effectively achieves multi-temperature control for diverse goods with varying temperature necessities, we anticipate its application in the thermal insulation domain, culminating in the creation of a multi-temperature maintenance container. Comparing the principles of a standard commercial thermal insulating container, a phase change thermal insulating container, and our proposed multi-temperature maintenance container (Supplementary Fig. 14), we delineate the unique attributes of the multi-temperature maintenance container.

To start, let us delve into the commercial thermal insulating container. Its primary function is to mitigate temperature fluctuations of the stored goods. This is achieved by enhancing the thermal resistance between the goods and the external environment.

Next, we turn our attention to the phase-change thermal insulating container. To augment the container's temperature-maintenance prowess, researchers have introduced a high-performance phase change material (PCM) — one whose phase transition temperature closely aligns with the temperature needs of the stored goods — into the standard thermal insulating container [21–23]. The substantial latent heat harnessed during the phase transitions of the PCMs can modulate the temperature within the control zone, substantially extending the temperature maintenance duration of the goods. When dealing with goods that have multi-temperature requirements, it becomes imperative to formulate high-performance PCMs with a variety of phase transition temperatures. Consider, for instance, goods with nine distinct temperature requirements: A spe-

cialized container can be designed using nine PCMs, each with a phase transition temperature that aligns with these specific demands. As highlighted in the main text, the primary challenges for such an arrangement stem from space efficiency, PCM development and inevitable heat transfer.

Lastly, we present our proposition: the multi-temperature maintenance container. As delineated in the manuscript, the AMTC can consistently maintain different zones at their requisite temperatures amidst a temperature differential. By merging the temperature-control capability of PCMs (Fig. 1b), we can instigate a temperature difference within the commercial thermal insulating container. This is achieved by employing two kinds of PCMs, each having either high or low phase transition temperatures. By aligning the temperature of the storage realm with the goods' initial temperature (or their temperature requirements), we can ensure multi-temperature maintenance for goods with diverse temperature prerequisites. As per the operational principles of the multi-temperature maintenance container, only two types of PCMs are necessary. Selecting two high-performance PCMs that fulfill these stipulations is easy. Even more crucially, in line with the traits of the multi-temperature control system, parameters like the number, dimensions, and temperature of the storage spaces remain adaptable. These facets set our multi-temperature maintenance container apart from conventional commercial thermal insulating containers and phase change thermal insulating containers.

13 Experimental setup for the container with and without AMTC.

13.1 Preparing the water with different initial temperatures

To counteract the effects of heat dissipation, we combined hot/cold water with normal-temperature water, enabling rapid preparation of water at varied initial temperatures. In line with the multi-temperature requirements [Eq. (2)], the volume of the normal-temperature water $\left(V_{\mathrm{nor}}\right)$ for each glassware was determined as

$$\begin{cases} V_\lambda T_\lambda + V_{\mathrm{nor}} T_{\mathrm{nor}} = V_{\mathrm{tot}} T_{\mathrm{o},\{i,j\}} \\ V_\lambda + V_{\mathrm{nor}} = V_{\mathrm{tot}} \end{cases}, \tag{S88}$$

where $\lambda = \mathbb{H},\ \mathbb{L}$; $\mathbb{H}$ $(\mathbb{L})$ denotes hot (cold) water; when $T_{\mathrm{o},\{i,j\}} > T_{\mathrm{nor}}$, $\lambda = \mathbb{H}$; when $T_{\mathrm{o},\{i,j\}} < T_{\mathrm{nor}}$, $\lambda = \mathbb{L}$; V_λ (T_λ) is volume (temperature) of hot/cold water; T_{nor} is temperature of normal-temperature water; V_{tot} is the total volume of the water in each glassware; $T_{\mathbb{H}}$, $T_{\mathbb{L}}$, and T_{nor} are 333.15 K, 278.15 K, and 294.15 K, respectively; V_{tot} is 300 mL. See V_{nor} for each glassware in Supplementary Table 5.

We introduced the predetermined volume of normal-temperature water into the respective glassware. Following that, cold/hot water was promptly poured up to the designated waterline (Supplementary Fig. 24). The deviations between the experimental outcomes and the desired values ranged from -1.90% to 1.37% (Supplementary Fig. 25).

13.2 Fabricating, preparation and installation of mobile heat and cold sources

In real-world applications, the packaging of PCMs must be addressed (Supplementary Fig. 16a). Initially, we added granulated stearic acid (PCM A) to beakers and subsequently placed them in a water bath ranging between 353.15 and 363.15 K. Once the stearic acid fully melted, we poured it into high-density polyethylene containers and allowed it to solidify. In a procedure similar to this, we filled these containers with distilled water (PCM B). Following the mentioned procedures, PCM A served efficiently as a mobile heat source, while PCM B functioned as a mobile cold source.

Subsequently, we detail the methods for preparing and installing the heat/cold sources. The heat source was immersed in a water bath, maintained at approximately 353.15 to 363.15 K, while the cold source underwent cooling in a refrigerator at temperatures below 250 K, as depicted in Supplementary Fig. 16b. When PCM A (PCM B) had completely melted (or solidified), it was removed from the water bath (or refrigerator) and positioned appropriately for use.

13.3 Experiment procedures for the multi-temperature maintenance test

The schematic representation for the methodology of the multi-temperature maintenance test is depicted in Supplementary Fig. 23.

The test procedure for the container equipped with AMTC (multi-temperature maintenance container) is as follows:

(1) Mark the position of the multi-temperature control system in a thermal insulating container (white regions).

(2) Fabricate the multi-temperature control system and place it into the specific position following the above mark;

(3) Place the glassware into the temperature control zone;

(4) Add the normal-temperature water of specific volumes into the corresponding glassware (Supplementary Note 13.1);

(5) Add hot/cold water up to the waterline into the corresponding glassware (Supplementary Note 13.1).

(6) Put the thermocouple through the foam cover into the water and then cover the foam cover.

(7) Place the heat/cold source (pre-heated/cooled) into the corresponding position. Then, insert thermocouples into the corresponding position to test the temperature of the heat/cold source.

(8) Close the lid of the container. Then, turn on the data collector and the computer to obtain the temperature-time curves of each simulacrum and the heat (cold) source in real-time.

For comparison, the multi-temperature maintenance test for the container without AMTC (referring to commercial thermal insulating container) should be executed under identical conditions:

(1) The same as step (1) of the container with AMTC.

(2) Place glassware according to the above mark.

(3) The same as step (4) of the container with AMTC.
(4) The same as step (5) of the container with AMTC.
(5) The same as step (6) of the container with AMTC.
(6) The same as step (8) of the container with AMTC.

It's worth noting that the experimental process for the container integrated with LTCM aligns closely with that of the AMTC container. The only distinctions involve substituting the heat and cold sources, as well as the AMTC, with materials exhibiting low thermal conductivity.

14 Additional research for multi-temperature maintenance container

14.1 Size enlargement

It is noted that the size of the glassware used is approximately 80mm×80mm×80mm. Using this site can demonstrate the effectiveness of AMTC for multi-temperature maintenance in practical applications. However, it is a larger storage space might be more appealing to users. To address this, we now include a brief discussion regarding AMTC with larger storage space. The discussion can be divided into two aspects: the analysis of the multi-temperature control mechanism of AMTC and the validation through finite element simulations.

In the first aspect, we provide a concise theoretical analysis. Referring to the conduction heat transfer system described in Fig. 2, the temperature difference in each temperature control zone can be expressed as

$$\Delta T_{\mathrm{c},\{m,j\}} = \frac{(T_{\mathrm{A}} - T_{\mathrm{B}}) d_{\mathrm{c},\{m,j\}} / \kappa_{\mathrm{c},\{m,j\}}}{\sum_{j\#=1}^{n} \sum_{\epsilon=\mathrm{l,c,r}} d_{\epsilon,\{m,j\#\}} / \kappa_{\epsilon,\{m,j\#\}}} \tag{S89}$$

It is evident that $\Delta T_{\mathrm{c},\{m,j\}}$ is independent of the size of the multi-temperature control system. In a steady state, maintaining the ratio of all structural parameters along the X-axis unchanged will preserve the original multi-temperature control performance.

For clarity, we establish two sets of structural parameters: one for a small size denoted as $d^{\mathrm{A}}_{\epsilon,\{m,j\}}$, and the other for a large size denoted as $d^{\mathrm{B}}_{\epsilon,\{m,j\}}$, where $d^{\mathrm{B}}_{\epsilon,\{m,j\}} = 1000 d^{\mathrm{A}}_{\epsilon,\{m,j\}}$. Now, let us consider a specific scenario. If our current operation is designed for a small size case with the structural parameters $d^{\mathrm{A}}_{\epsilon,\{m,j\}}$ determined based on requirements, as described in Supplementary Note 5, we can solve all the thermal parameters $\kappa_{\epsilon,\{m,j\}}$. Consequently, the multi-temperature control system for the small-size case is successfully designed. Subsequently, we consider the large-size case. A straightforward approach is to replace $d^{\mathrm{A}}_{\epsilon,\{m,j\}}$ with $d^{\mathrm{B}}_{\epsilon,\{m,j\}}$. Rewriting Eq. (S89), we obtain

$$\Delta T_{\mathrm{c},\{m,j\}} = \frac{(T_{\mathrm{A}} - T_{\mathrm{B}}) d^{\mathrm{B}}_{\mathrm{c},\{m,j\}} / \kappa_{\mathrm{c},\{m,j\}}}{\sum_{j\#=1}^{n} \sum_{\epsilon=\mathrm{l,c,r}} d^{\mathrm{B}}_{\epsilon,\{m,j\#\}} / \kappa_{\epsilon,\{m,j\#\}}} \tag{S90}$$

Taking into account the relationship between $d^{\mathrm{A}}_{\epsilon,\{m,j\}}$ and $d^{\mathrm{B}}_{\epsilon,\{m,j\}}$, Eq. (S90) can be rewritten as

$$\Delta T_{\mathrm{c},\{m,j\}} = \frac{(T_{\mathrm{A}} - T_{\mathrm{B}})1000 d^{\mathrm{A}}_{\mathrm{c},\{m,j\}} / \kappa_{\mathrm{c},\{m,j\}}}{\sum_{j\#=1}^{n} \sum_{\epsilon=\mathrm{l,c,r}} 1000 d^{\mathrm{A}}_{\epsilon,\{m,j\#\}} / \kappa_{\epsilon,\{m,j\#\}}} = \frac{(T_{\mathrm{A}} - T_{\mathrm{B}}) d^{\mathrm{A}}_{\mathrm{c},\{m,j\}} / \kappa_{\mathrm{c},\{m,j\}}}{\sum_{j\#=1}^{n} \sum_{\epsilon=\mathrm{l,c,r}} d^{\mathrm{A}}_{\epsilon,\{m,j\#\}} / \kappa_{\epsilon,\{m,j\#\}}} \tag{S91}$$

The consistency between Eq. (S90) and Eq. (S91) ensures that when the device is scaled up, the multi-temperature control effect can be maintained in a steady state. Taking the case depicted in Fig. 2a as an example, we enlarged the system size from ”mm” to ”m” and conducted simulations. The comparison between these two cases is presented in Supplementary Fig. 27. Supplementary Table 6 displays the maximum/average/minimum temperatures of each temperature control zone. Notably, the temperature distribution in both cases is identical, demonstrating strong consistency with the aforementioned results.

Additionally, if users aim to further increase the storage space in the aforementioned large-size case while keeping the overall system size constant, they may consider reducing the sizes of zones $d_{\mathrm{l},\{m,j\}}$ and $d_{\mathrm{r},\{m,j\}}$. Although this adjustment would disrupt the aforementioned ratio of structural parameters, our theory can solve this issue by redesigning the thermal parameters $\kappa_{\epsilon,\{m,j\}}$, as shown in Supplementary Note 5.

From an application perspective, it is essential to examine the transient state of the multi-temperature maintenance performance of the container with AMTC. Following the simulation method of Fig. 6b, we enlarged the container size from ”mm” to ”m” and conducted simulations, as shown in Supplementary Fig. 28a. The results demonstrate that within two hours, the temperature of objects in each zone can be effectively maintained at a constant level.

To further investigate, we set up a specific case wherein objects in the high-temperature region are removed and replaced with normal-temperature air, as shown in Supplementary Fig. 28b. It is observed that the temperature of the objects in the remaining areas can still be adequately maintained. Based on the analysis, while enlarging the size prolongs the action time of the heat source and cold source on temperature control in each zone, the increased storage volume of objects in each zone also enhances the sensible heat storage capacity. Consequently, the system remains less susceptible to the external environment over a short period, exhibiting stable multi-temperature maintenance performance. These findings indicate that enlarging the size of a multi-temperature maintenance container does not compromise its performance, thus benefiting practical applications.

14.2 Thermal inertia

Thermal inertia is crucial in the context of the AMTC system as it affects the cascading of temperature levels and plays a significant role in maintaining stable temperature conditions. In this discussion, we aim to explore the factors influencing thermal inertia and their specific effects.

The motivation behind optimizing thermal inertia is to enhance the effectiveness of multi-temperature maintenance by improving the sensible heat (cold) storage capacity, represented by Q_{HS} (Q_{CS}). Increasing the thermal inertia allows for better temperature control and reduces temperature variations of the simulacrum in each zone.

There are two main factors that can be optimized to increase thermal inertia:

1. Increase the specific heat capacity c_p and density ρ of each simulacrum: By increasing c_p and ρ of each simulacrum (goods), Q_HS (Q_CS) can be increased. This results in larger thermal inertia and better temperature maintenance. For example, setting c_p to 1000 J kg^{-1} K^{-1}, 2000 J kg^{-1} K^{-1}, and 3000 J kg^{-1} K^{-1}, and ρ to 2000 $\mathrm{kg\,m^{-3}}$, 3000 $\mathrm{kg\,m^{-3}}$, and 4000 $\mathrm{kg\,m^{-3}}$, allows us to observe the effects of c_p and ρ on multi-temperature maintenance by analyzing the temperature-time curves of each simulacrum in the container.

2. Adjust the initial temperatures $T_{\gamma,\mathrm{in}}$ of simulacra: Another approach to optimize thermal inertia is by adjusting $T_{\gamma,\mathrm{in}}$ of simulacra in the high- and low-temperature regions. Increasing the temperatures of simulacra in the high-temperature region by 1 K, 2 K, or 3 K, while simultaneously decreasing the temperatures of simulacra in the low-temperature region by 1 K, 2 K, or 3 K, allows us to evaluate the impact of these temperature adjustments on multi-temperature maintenance.

The simulation results are presented in Supplementary Fig. 29. It can be observed that increasing the sensible heat (cold) storage capacity by increasing the specific heat capacity and density of each simulacrum is beneficial for reducing temperature variations. Additionally, adjusting the initial temperatures of simulacra in the high- and low-temperature regions can further increase the effective time of multi-temperature maintenance. This adjustment acts as pre-cooling and pre-heating, compensating for heat or cooling losses caused by heat transfer between the simulacra and the system in the initial stage.

In conclusion, optimizing thermal inertia through increased sensible heat (cold) storage capacity can enhance the performance of the AMTC system, leading to improved multi-temperature maintenance and reduced temperature variations.

14.3 Wrong placement

The motivation behind studying wrongly placed simulacra is to understand the thermal consequences of misplacement and its practical implications for multi-temperature maintenance. Experiments were conducted to explore this scenario.

In the experiments, two cases of misplacement were considered, as shown in Supplementary Fig. 30a.

1. The simulacra were misplaced in the high-, middle-, and low-temperature regions, respectively.

2. The simulacrum was misplaced in the low-temperature region when it should be in the high-temperature region. Conversely, the simulacrum intended for the low-temperature region was mistakenly placed in the high-temperature region.

The experimental results, shown in Supplementary Fig. 30b, indicate that the first case does not exhibit a significant effect. However, in the second case, the temperature of the simulacra at the misplaced positions changed considerably. To provide a quantitative description, the temperature variation rate of each simulacrum was analyzed after two hours, as depicted in Supplementary Fig. 30c. The correct results were also marked as circles in the figure for reference.

Based on the analysis, it can be concluded that if a misplaced simulacrum is within the same temperature region (high temperature/middle temperature/low temperature), it does not have a

significant impact on the temperature maintenance performance. However, if a misplaced simulacrum crosses different temperature regions, such as the highest temperature object and the lowest temperature object being placed incorrectly, it affects only the temperature maintenance performance of those two misplaced simulacra, while having no impact on that of other simulacra. The extent of the impact depends on the deviation between the preset temperature of the temperature control zone and the temperature of the misplaced simulacra. The deviation is small within the same temperature region but larger across different temperature zones.

In practical applications, users can easily identify the location of the high-temperature region and the low-temperature region due to the significant temperature difference between the mobile heat source and the mobile cold source. Hence, under normal circumstances, misplacement is more likely to occur within the same temperature region. Consequently, misplacement does not have a substantial impact on the multi-temperature maintenance effect.

15 Exploring the correlation between effective temperature maintenance time and system parameters

To simplify our analysis, we posited a uniform temperature distribution within the PCMs during the phase transition. The heat flow from PCM A to PCM B is given by

$$Q_{\mathrm{A}\to\mathrm{B}} = \sum_{i=1}^{m} A_{\mathrm{A}\to\mathrm{B},i} q_i = \sum_{i=1}^{m} A_{\mathrm{A}\to\mathrm{B},i} \frac{T_{\mathrm{p,A}} - T_{\mathrm{p,B}}}{\sum_{j=1}^{n} \sum_{\epsilon=\mathrm{l,c,r}} \frac{d_{\epsilon,\{i,j\}}}{\kappa_{\epsilon,\{i,j\}}}}. \tag{S92}$$

Here, $A_{\mathrm{A}\to\mathrm{B},i}$ and q_i represent the heat transfer area and heat flux from PCM A to PCM B on row i, respectively.

The heat flow from PCM A to the environment and that from the environment to PCM B are given by

$$\begin{cases} Q_{\mathrm{A}\to\mathrm{E}} = \dfrac{A_{\mathrm{A}\to\mathrm{E}}(T_{\mathrm{p,A}} - T_{\mathrm{E}})}{R_{\mathrm{A}\to\mathrm{E}}} \\ Q_{\mathrm{E}\to\mathrm{B}} = \dfrac{A_{\mathrm{E}\to\mathrm{B}}(T_{\mathrm{E}} - T_{\mathrm{p,B}})}{R_{\mathrm{E}\to\mathrm{B}}} \end{cases}, \tag{S93}$$

where $A_{\mathrm{A}\to\mathrm{E}}$ and $A_{\mathrm{E}\to\mathrm{B}}$ represent the heat transfer areas beween the PCM A and environment, and between the environment and PCM B, respectively. For simplicity, we define the thermal resistance between PCM A (and PCM B) and the environment as $R_{\mathrm{A}\to\mathrm{E}}$ ($R_{\mathrm{E}\to\mathrm{B}}$). The effective temperature control times for PCM A and PCM B are

$$\begin{cases} t_{\mathrm{A}} = \dfrac{L_{\mathrm{A}}}{Q_{\mathrm{A}\to\mathrm{B}} + Q_{\mathrm{A}\to\mathrm{E}}} \\ t_{\mathrm{B}} = \dfrac{L_{\mathrm{B}}}{Q_{\mathrm{A}\to\mathrm{B}} + Q_{\mathrm{E}\to\mathrm{B}}} \end{cases}, \tag{S94}$$

where L_{A} and L_{B} are the latent heats of PCM A and PCM B, respectively. In line with the AMTC principle, PCM A and PCM B should supply constant temperature boundaries on the

left and right, respectively. Thus, the effective temperature control (maintenance) time is the minimum of t_{A} and t_{B}

$$t_{\mathrm{eff}} = \mathrm{Min}(t_{\mathrm{A}}, t_{\mathrm{B}}). \tag{S95}$$

From Eq. (S92)-Eq. (S94), we observe that decreasing the temperature difference between the phase change temperatures of PCM A (or PCM B) and the environmental temperature, increasing the thermal resistance between PCM A and PCM B, and boosting the latent heat can enhance t_{eff}. In conjunction with the AMTC principle, opting for a PCM A (or PCM B) with a phase change temperature slightly above (or below) the maximum (or minimum) temperature requisite for the goods, and with a high latent heat, can be advantageous. In fulfilling the uniform temperature condition requirements, reducing the thermal conductivity in zones $\mathrm{L}_{\{i,j\}}$ and $\mathrm{R}_{\{i,j\}}$ aids in augmenting t_{eff}. Furthermore, augmenting the size to increase $d_{\epsilon,\{i,j\}}$ boosts the thermal resistance between PCM A and PCM B, which aligns with practical application needs for larger storage spaces.

For future advancements, integrating the thermal properties of actual composite PCMs from relevant references with the previously discussed effective temperature maintenance time may aid in selecting suitable pairs of heat and cold sources and system's thermal/structural parameters. This could ensure reliable goods transportation over extended durations.

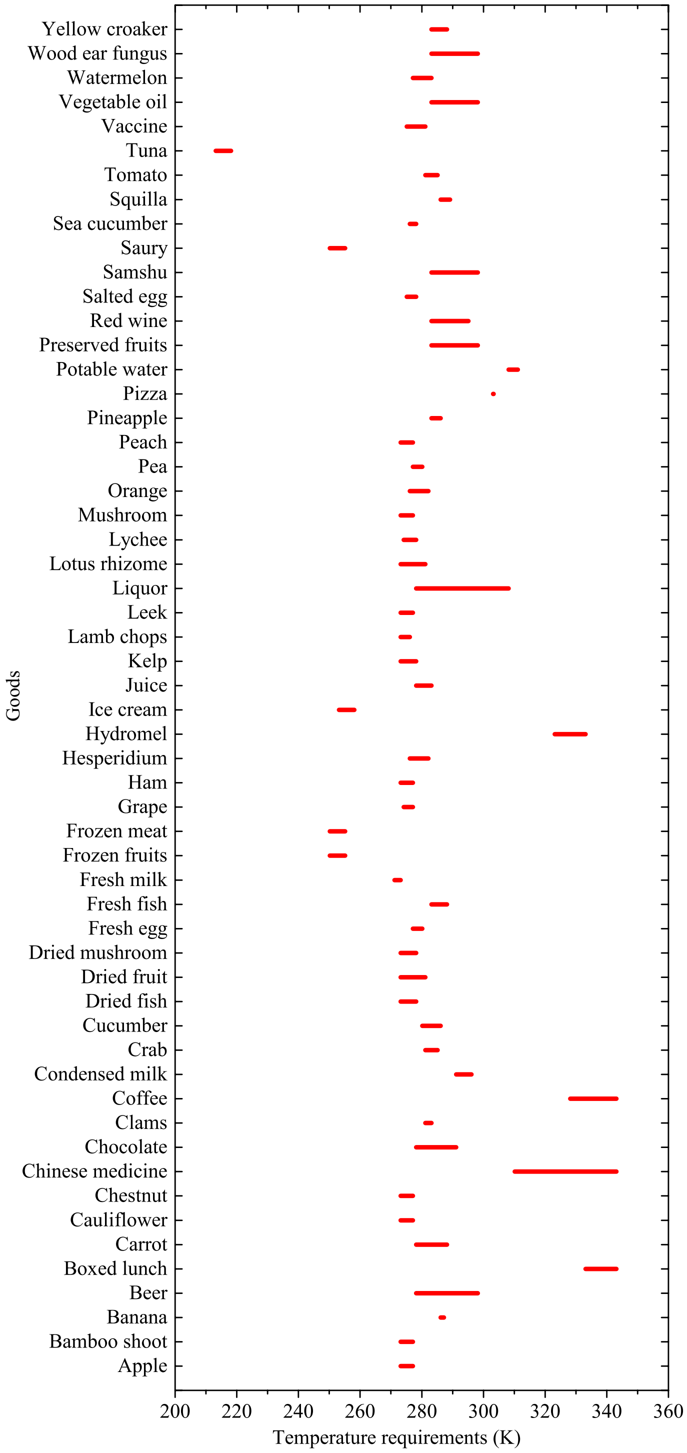

Supplementary Fig. 1|Temperature requirements of various transported goods. The red lines (dot) illustrate the temperature requirement ranges for different goods. Primary data were sourced from Refs. 24–28. Information regarding the temperature for pizza was gleaned from product descriptions of thermostats designed for fast food storage available on the Jindong platform. Data for boxed lunches were referenced from news and policy reports. When particular goods require their temperature to be either above (or below) the specific $T_{\mathscr{C}}$, for clarity in presentation, we denoted their temperature range as $[T_{\mathscr{C}}, T_{\mathscr{C}} + 5]$ K ($[T_{\mathscr{C}} - 5, T_{\mathscr{C}}]$ K). Source data are provided as a Source Data file.

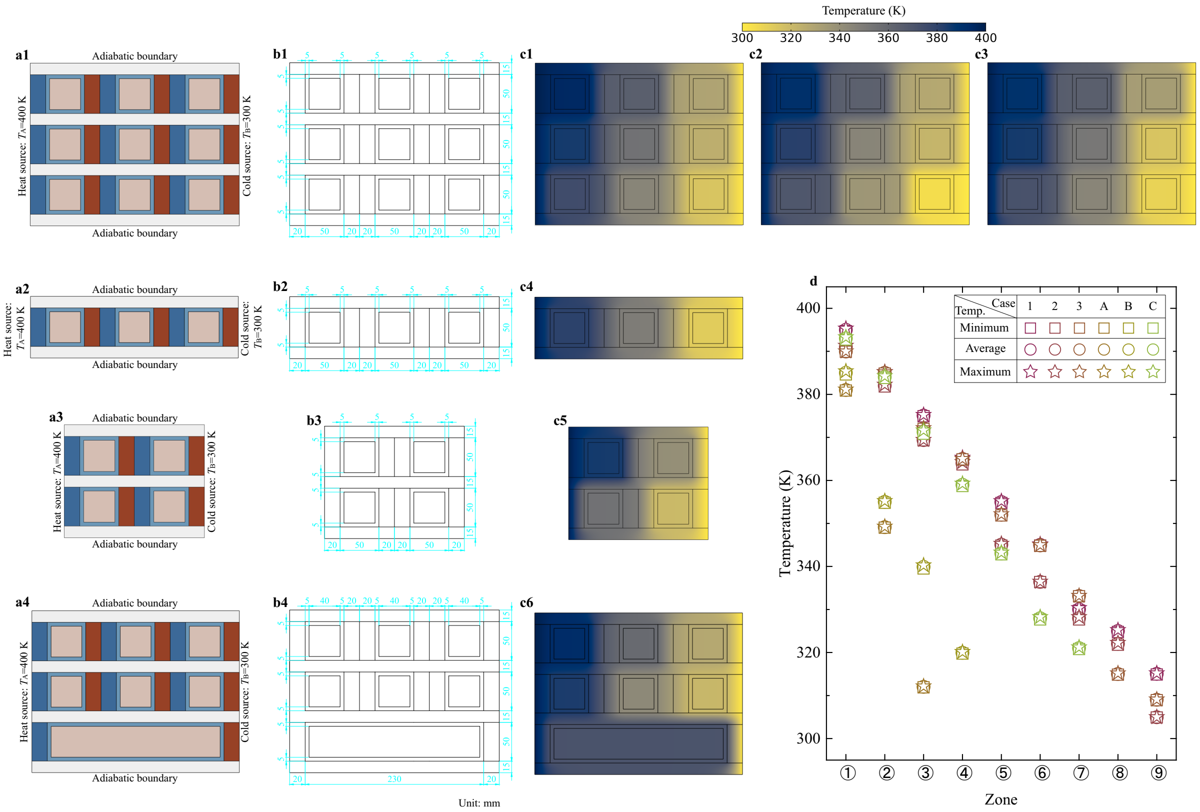

Supplementary Fig. 2|Multi-temperature control system for diverse goods requirements. a1,b1, Geometry (**a1**) and structural parameter (**b1**) of the multi-temperature control system depicted in Fig. 4a. **c1,c2,c3,** Temperature distributions of the multi-temperature control system corresponding to varied multi-temperature requirements: Eq. (S60) (**c1**), Eq. (S61) (**c2**), and Eq. (S62) (**c3**). **a2,b2,c4,** Geometry (**a2**), structural parameter (**b2**), and temperature distribution (**c4**) of the multi-temperature control system in relation to Eq. (S63). **a3,b3,c5,** Geometry (**a3**), structural parameter (**b3**), and temperature distribution (**c5**) of the multi-temperature control system in accordance with Eq. (S64). **a4,b4,c6,** Geometry (**a4**), structural parameter (**b4**), and temperature distribution (**c6**) of the multi-temperature control system pertaining to Eq. (S65). **d,** Minimum/average/maximum temperature of the goods stored in each temperature control zone correspond to cases 1 (**c1**), 2 (**c2**), 3 (**c3**), A (**c4**), B (**c5**), and C (**c6**). Except for the thermal parameters described in Eqs. (S60)-(S65), the remaining thermal parameters for simulations were consistent with those of Supplementary Fig. 5a. Note that we use the temperature range 300-400 K for simplicity in presentation. Adhering to the principle of AMTC, this approach retains its generality. Instances where the temperature control zones precisely align with the actual goods in Supplementary Fig. 1 are displayed in Fig. 3. Source data are provided as a Source Data file.

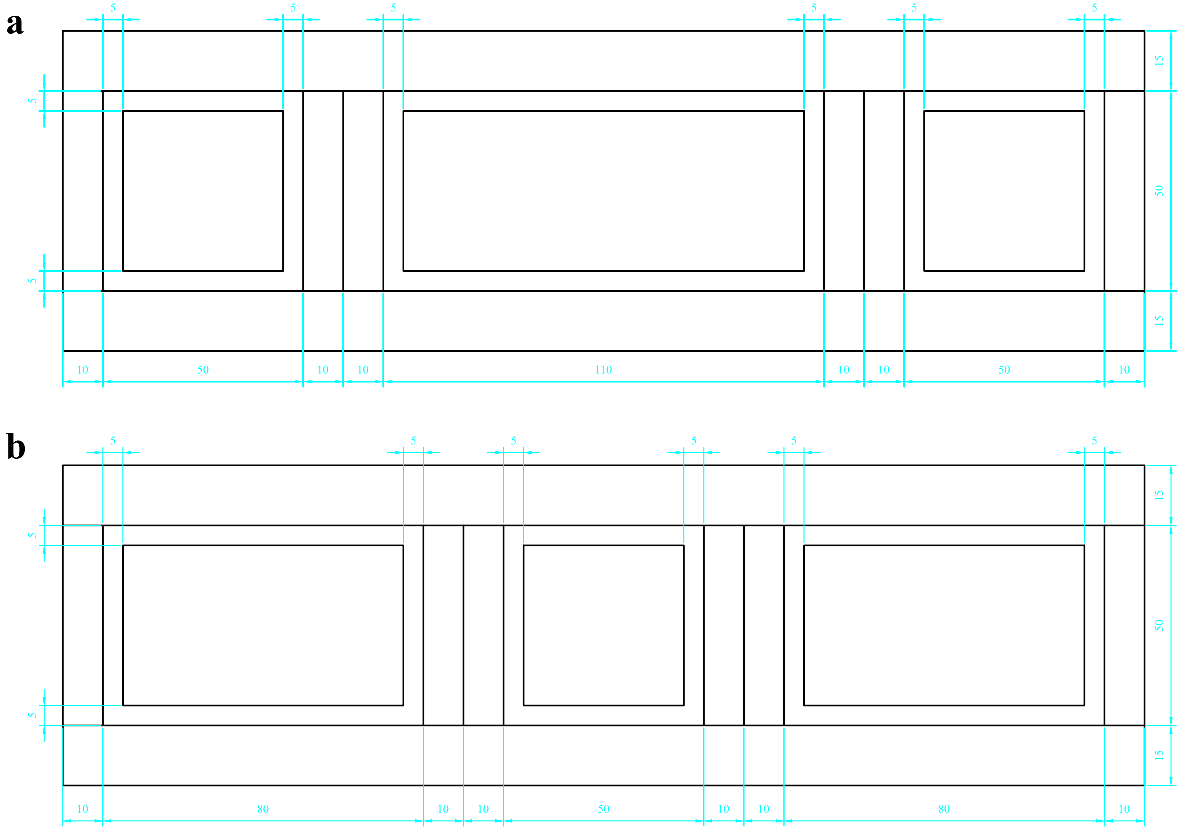

Unit: mm

Supplementary Fig. 3|Structural parameters of the multi-temperature control system. a and **b** represent the structural parameters of Fig. 3b and c, respectively.

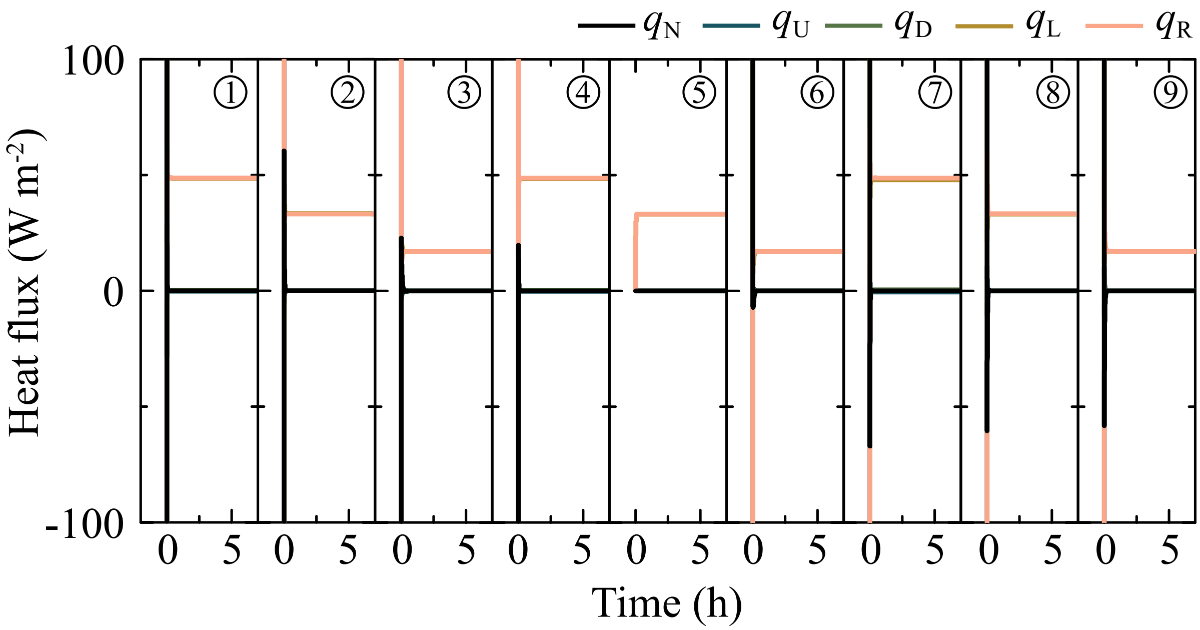

Supplementary Fig. 4|Detailed heat flux of each temperature control zone for the upper subgraph in Fig. 4d. Note that the lines for q_L are obscured by those for q_R.

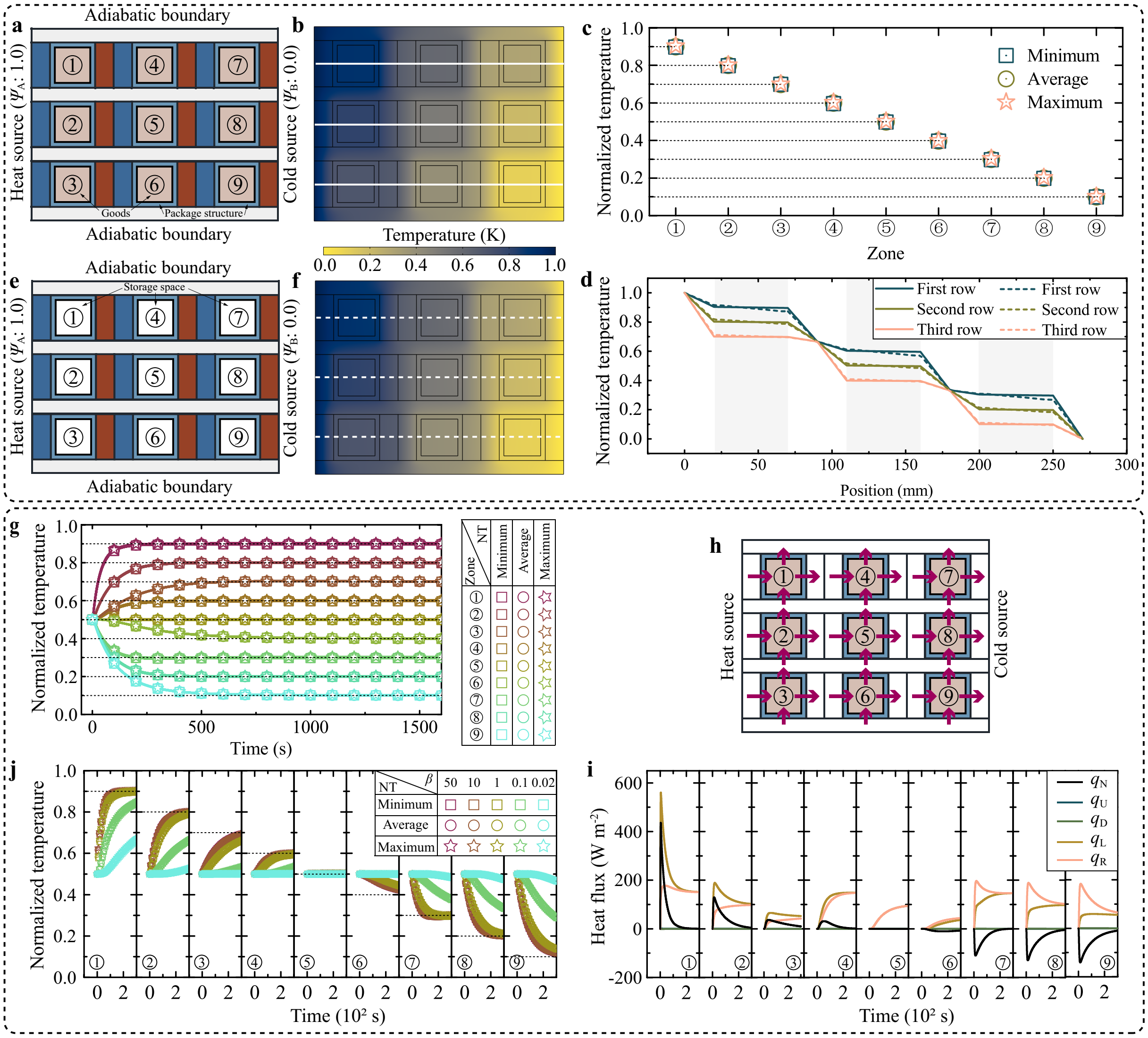

Supplementary Fig. 5|Steady and transient multi-temperature control characteristics. (**a** to **f**) A two-dimensional multi-temperature control system and its steady temperature control characteristics. The normalized temperature of the heat (cold) source is 1.0 (0.0). Refer to the desired normalized temperature of each temperature control zone in "Application of AMTC: multi-temperature maintenance" (Fig. 4a, color bar). **a,** A schematic diagram of a multi-temperature control system with nine temperature control zones filled with goods. See the thermal/structural parameters of the system in Supplementary Note 4. **b,** Normalized temperature distribution of the system corresponds to **a**. **c,** Minimum/average/maximum normalized temperature of the goods corresponding to **a**. The black dashed lines represent the required temperature of the goods in each temperature control zone (also exist in **g** and **j**). **d,** Temperature distributions on the characteristic lines in **b** and **f**. The grey background represents temperature control zones. **e,** A scenario where the storage space is empty and filled with air. **f,** Normalized temperature distribution of the system corresponding to **e**. The solid (dashed) lines in white depict the terraced normalized temperature distributions of **b** (**f**) in the first, second, and third rows, respectively. (**g** to **j**) Transient temperature control characteristics of the system and the heat flux analysis. **g,** Goods' minimum/average/maximum normalized temperature (NT) over time. **h,** A schematic diagram for the heat flux across each temperature control zone. **i,** Heat flux over time for each temperature control zone. **j,** Effect of thermal diffusivity on the normalized temperature (NT) over time. β is a characteristic parameter of the thermal diffusivity of the components in the system. The larger the β, the larger the thermal diffusivity. Note that the symbols for $\beta = 10$ nearly overlap those for $\beta = 50$. Source data are provided as a Source Data file.

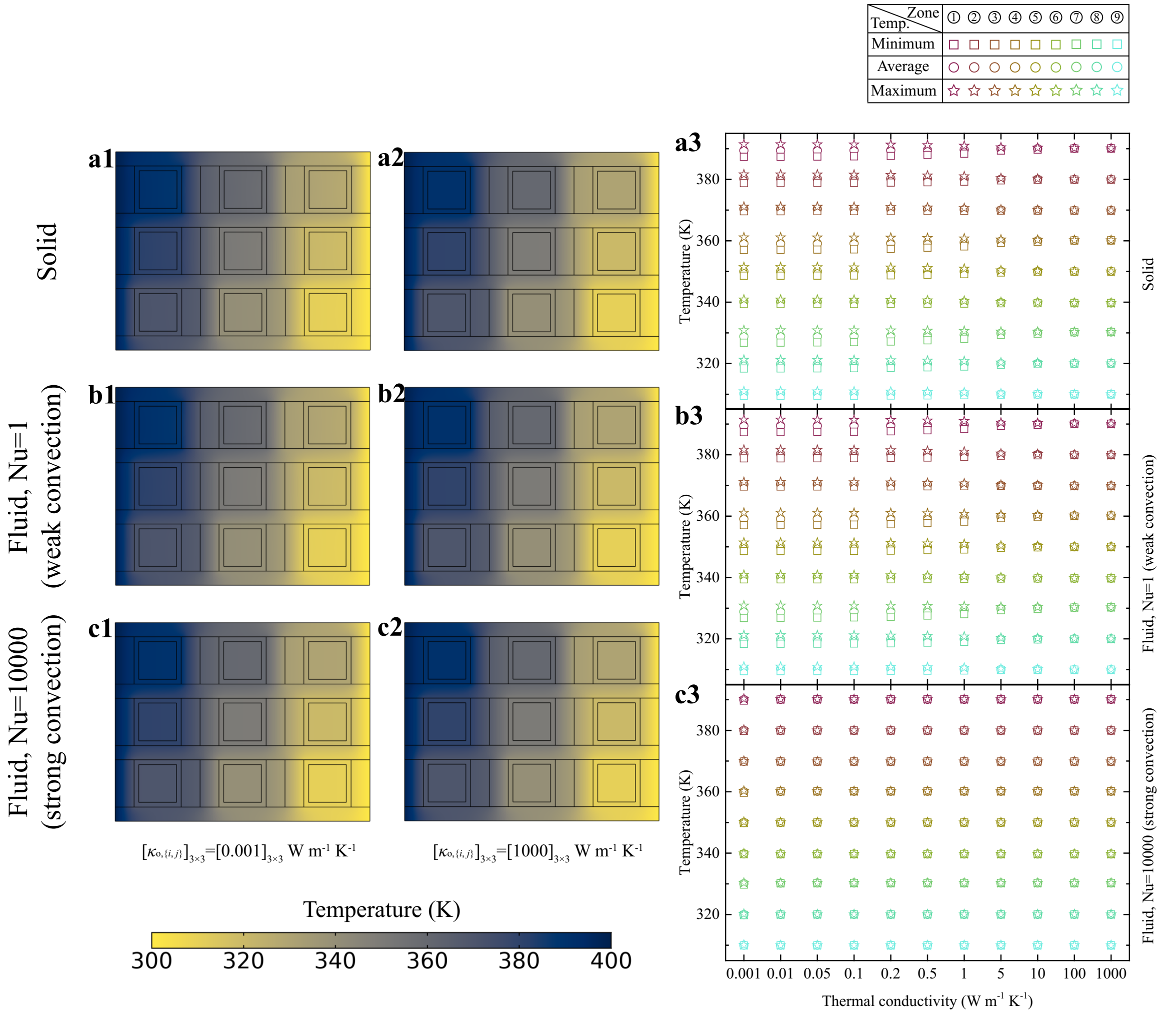

Supplementary Fig. 6|Influence of the thermal conductivities/phases of the goods on the multi-temperature control performance. a1,a2, Temperature distributions of the multi-temperature control system when the goods are solid with $\left[\kappa_{\mathrm{o},\{i,j\}}\right]_{3\times3} = [0.001]_{3\times3}$ W m^{-1} K^{-1} (**a1**) and $\left[\kappa_{\mathrm{o},\{i,j\}}\right]_{3\times3} = [1000]_{3\times3}$ W m^{-1} K^{-1} (**a2**). **b1,b2,** Temperature distributions of the multi-temperature control system when the goods are fluid (Nu=1) with $\left[\kappa_{\mathrm{o},\{i,j\}}\right]_{3\times3} = [0.001]_{3\times3}$ W m^{-1} K^{-1} (**b1**) and $\left[\kappa_{\mathrm{o},\{i,j\}}\right]_{3\times3} = [1000]_{3\times3}$ W m^{-1} K^{-1} (**b2**). **c1,c2,** Temperature distributions of the multi-temperature control system when the goods are fluid (Nu=10000) with $\left[\kappa_{\mathrm{o},\{i,j\}}\right]_{3\times3} = [0.001]_{3\times3}$ W m^{-1} K^{-1} (**c1**) and $\left[\kappa_{\mathrm{o},\{i,j\}}\right]_{3\times3} = [1000]_{3\times3}$ W m^{-1} K^{-1} (**c2**). **a3,b3,c3,** Minimum/average/maximum temperatures of the goods stored in the temperature control zones with varying thermal conductivities correspond to the solid state (**a3**), fluid state with Nu=1 (**b3**), and fluid state with Nu=10000 (**c3**). **Simulation details:** We used "convection-enhanced thermal conductivity" in COMSOL Multiphysics[29] to simulate the goods in the liquid state with differing Nu. The specific heat capacity, density, dynamic viscosity, and specific heat ratio of the goods in the fluid state were 100 J kg^{-1} K^{-1}, 100 kg m^{-3}, 0.001 Pa s, and 1, respectively. The remaining thermal parameters were consistent with those of Supplementary Fig. 5a. Source data are provided as a Source Data file.

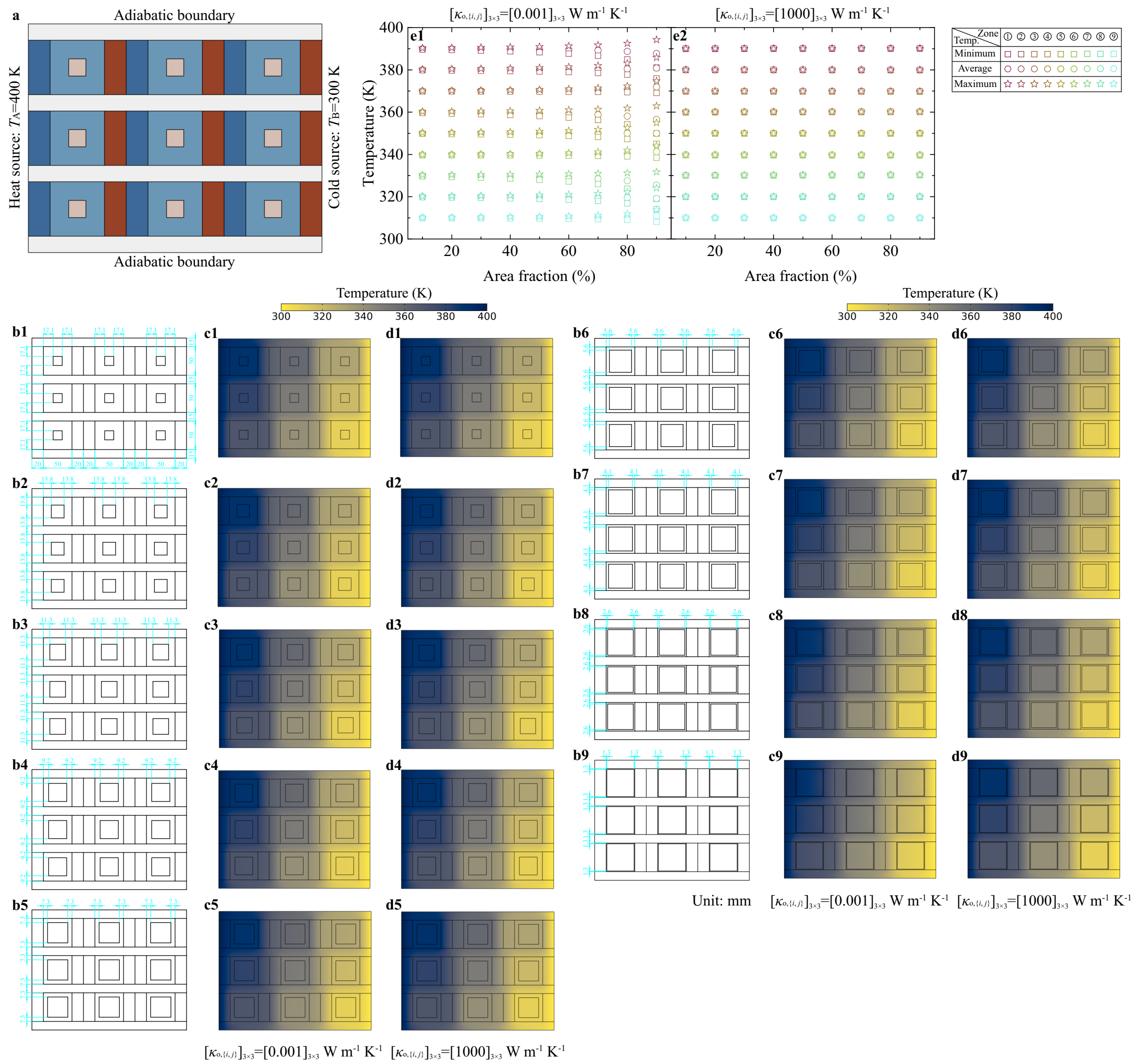

Supplementary Fig. 7|Influence of the area fractions of the square goods on the multi-temperature control performance. a, Geometry of the multi-temperature control system. The areas of the square goods are variable. **b1-b9,** Structural parameters of the multi-temperature control system with goods of different area fractions: 10% (**b1**), 20% (**b2**), 30% (**b3**), 40% (**b4**), 50% (**b5**), 60% (**b6**), 70% (**b7**), 80% (**b8**), and 90% (**b9**). **c1-c9,** Temperature distributions of the multi-temperature control system in the case where $\left[\kappa_{o,\{i,j\}}\right]_{3\times3} = [0.001]_{3\times3}$ W m^{-1} K^{-1} with the goods of different area fractions: 10% (**c1**), 20% (**c2**), 30% (**c3**), 40% (**c4**), 50% (**c5**), 60% (**c6**), 70% (**c7**), 80% (**c8**), and 90% (**c9**). **d1-d9,** Temperature distributions of the multi-temperature control system in the case where $\left[\kappa_{o,\{i,j\}}\right]_{3\times3} = [1000]_{3\times3}$ W m^{-1} K^{-1} with the goods of different area fractions: 10% (**d1**), 20% (**d2**), 30% (**d3**), 40% (**d4**), 50% (**d5**), 60% (**d6**), 70% (**d7**), 80% (**d8**), and 90% (**d9**). **e1,e2,** Minimum/average/maximum temperature of the goods stored in the temperature control zones with different area fractions in the case where $\left[\kappa_{o,\{i,j\}}\right]_{3\times3} = [0.001]_{3\times3}$ W m^{-1} K^{-1} (**e1**) and $\left[\kappa_{o,\{i,j\}}\right]_{3\times3} = [1000]_{3\times3}$ W m^{-1} K^{-1} (**e2**). The remaining thermal parameters for simulations were consistent with those in Supplementary Fig. 5a. Source data are provided as a Source Data file.

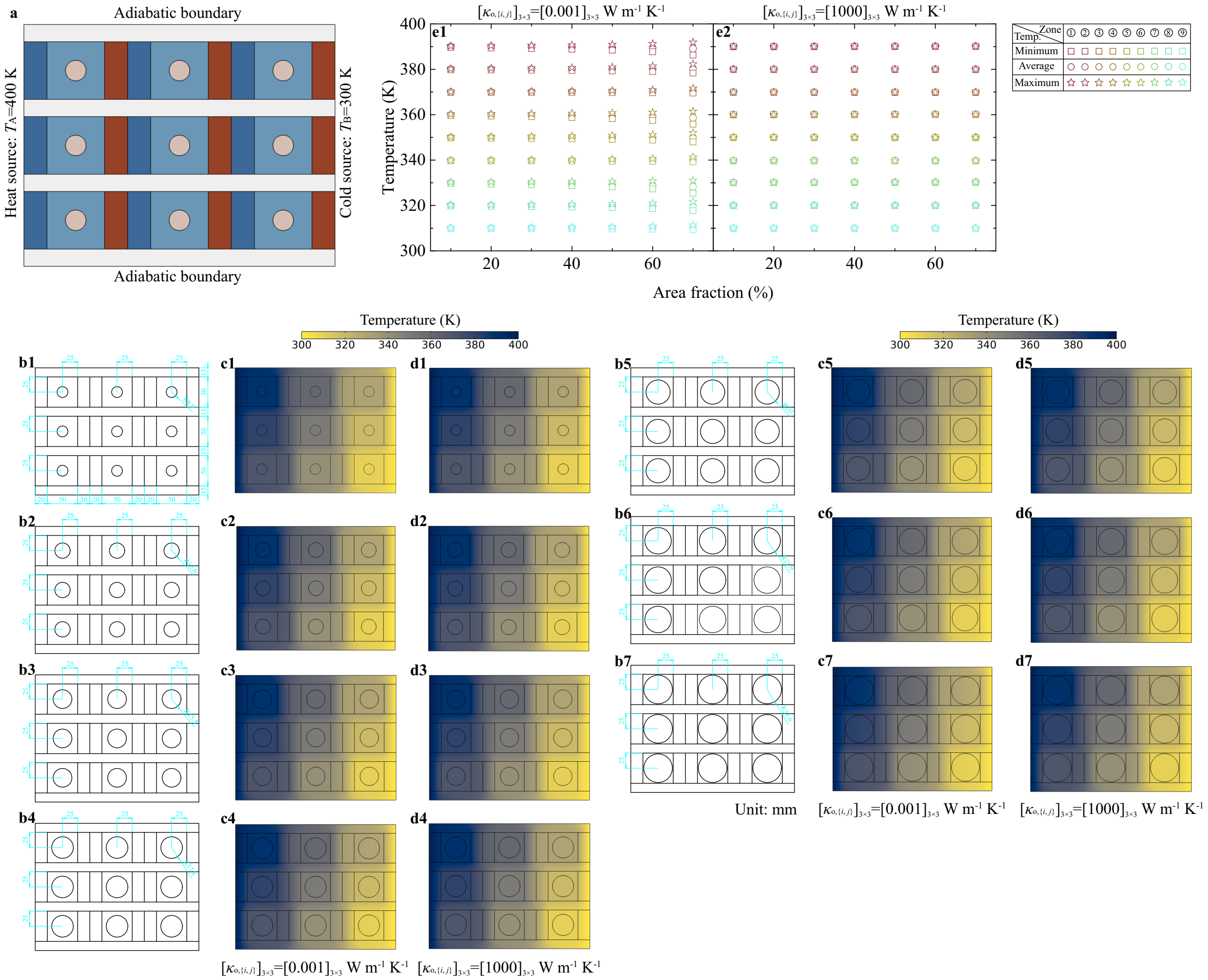

Supplementary Fig. 8|Influence of the area fractions of the circular goods on the multi-temperature control performance. a, Geometry of the multi-temperature control system. The areas of the circular goods vary. **b1-b7,** Structural parameters of the multi-temperature control system with goods of different area fractions: 10% (**b1**), 20% (**b2**), 30% (**b3**), 40% (**b4**), 50% (**b5**), 60% (**b6**), and 70% (**b7**). **c1-c7,** Temperature distributions of the multi-temperature control system when $\left[\kappa_{o,\{i,j\}}\right]_{3\times3} = [0.001]_{3\times3}$ W m^{-1} K^{-1} with goods of different area fractions: 10% (**c1**), 20% (**c2**), 30% (**c3**), 40% (**c4**), 50% (**c5**), 60% (**c6**), and 70% (**c7**). **d1-d7,** Temperature distributions of the multi-temperature control system when $\left[\kappa_{o,\{i,j\}}\right]_{3\times3} = [1000]_{3\times3}$ W m^{-1} K^{-1} with goods of different area fractions: 10% (**d1**), 20% (**d2**), 30% (**d3**), 40% (**d4**), 50% (**d5**), 60% (**d6**), and 70% (**d7**). **e1,e2,** Minimum/average/maximum temperature of the goods stored in the temperature control zones with varying area fractions when $\left[\kappa_{o,\{i,j\}}\right]_{3\times3} = [0.001]_{3\times3}$ W m^{-1} K^{-1} (**e1**) and $\left[\kappa_{o,\{i,j\}}\right]_{3\times3} = [1000]_{3\times3}$ W m^{-1} K^{-1} (**e2**). The remaining thermal parameters were identical to those in Supplementary Fig. 5a. Source data are provided as a Source Data file.

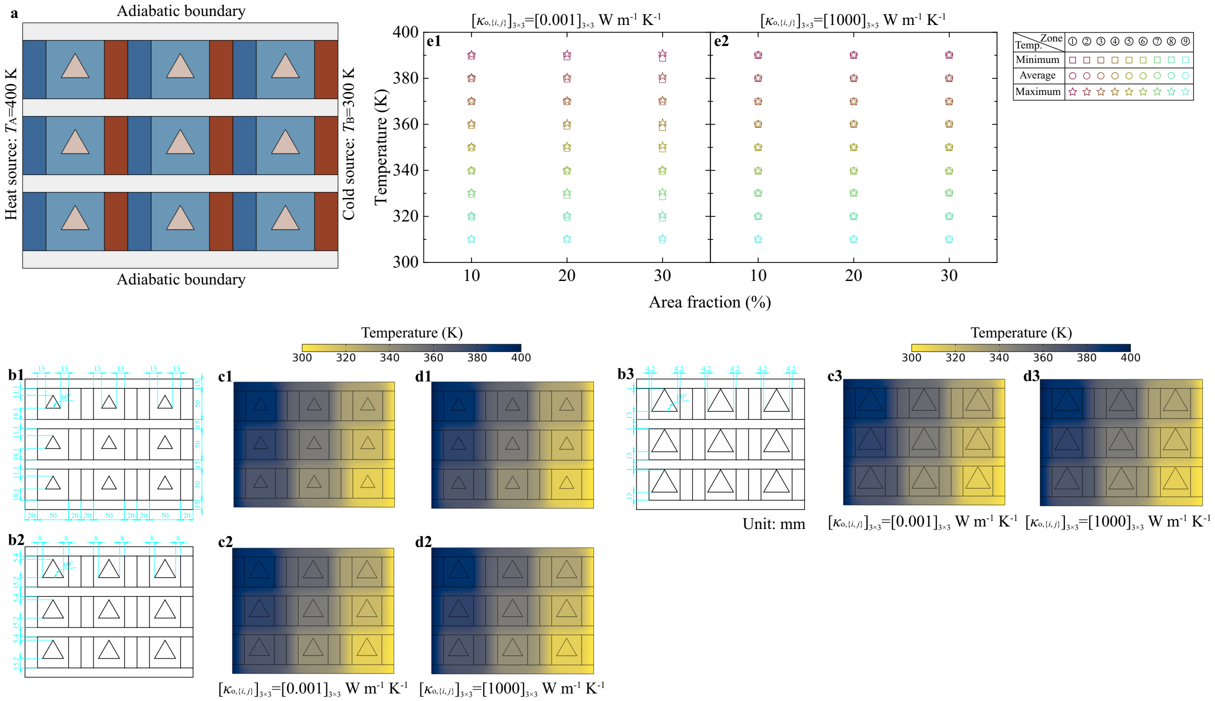

Supplementary Fig. 9|Influence of the area fractions of the triangular goods on the multi-temperature control performance. a, Geometry of the multi-temperature control system. The areas of the triangular goods vary. **b1-b3,** Structural parameters of the multi-temperature control system with goods of different area fractions: 10% (**b1**), 20% (**b2**), and 30% (**b3**). **c1-c3,** Temperature distributions of the multi-temperature control system when $[\kappa_{o,\{i,j\}}]_{3\times3} = [0.001]_{3\times3}$ W m^{-1} K^{-1} with goods of varying area fractions: 10% (**c1**), 20% (**c2**), and 30% (**c3**). **d1-d3,** Temperature distributions of the multi-temperature control system when $[\kappa_{o,\{i,j\}}]_{3\times3} = [1000]_{3\times3}$ W m^{-1} K^{-1} with goods of varying area fractions: 10% (**d1**), 20% (**d2**), and 30% (**d3**). **e1,e2,** Minimum/average/maximum temperature of the goods stored in the temperature control zones with varying area fractions when $[\kappa_{o,\{i,j\}}]_{3\times3} = [0.001]_{3\times3}$ W m^{-1} K^{-1} (**e1**) and $[\kappa_{o,\{i,j\}}]_{3\times3} = [1000]_{3\times3}$ W m^{-1} K^{-1} (**e2**). The remaining thermal parameters were identical to those in Supplementary Fig. 5a. Source data are provided as a Source Data file.

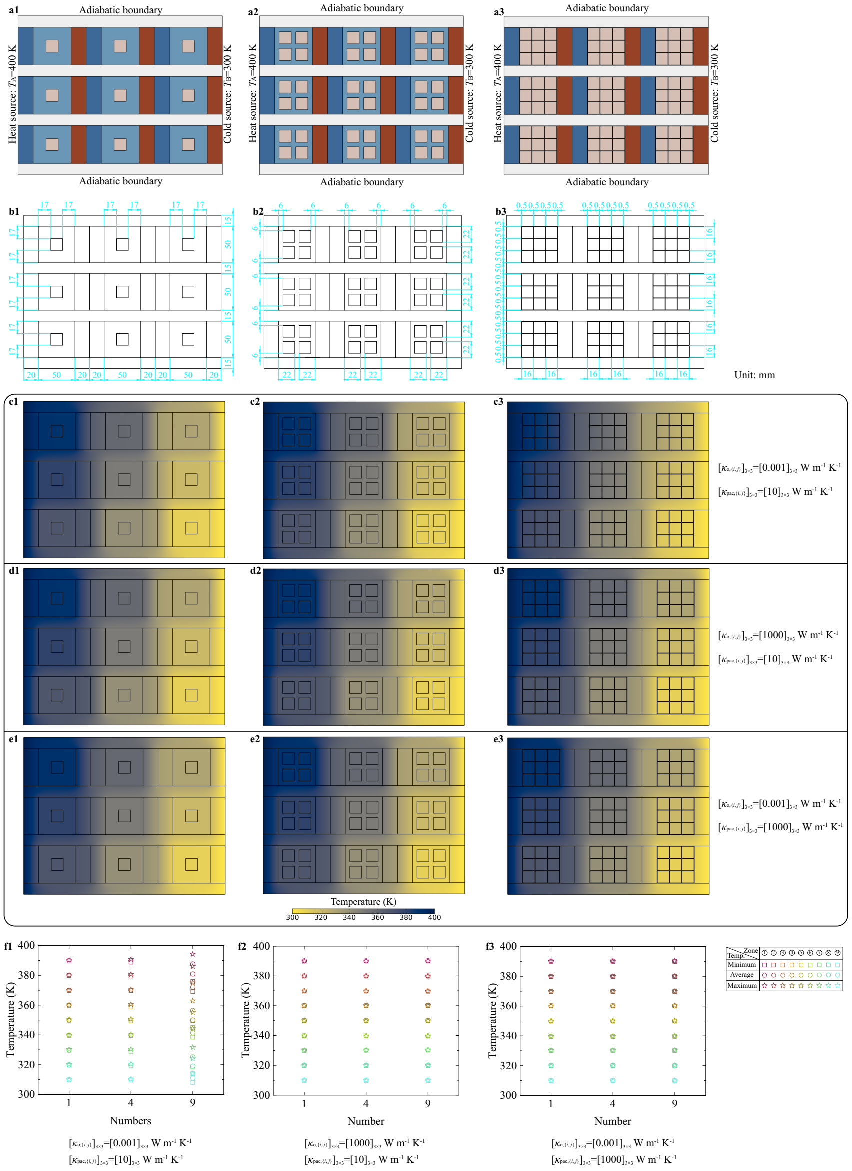

Supplementary Fig. 10|Influence of the numbers of goods in temperature control zones on the multi-temperature control performance. **a1-a3,** Geometries of the multi-temperature control system with one item of goods (**a1**), three items of goods (**a2**), and nine items of goods (**a3**). **b1-b3,** Structural parameters of the multi-temperature control system with one item of goods (**b1**), three items of goods (**b2**), and nine items of goods (**b3**). **c1-c3,** Temperature distributions of the multi-temperature control system with one item of goods (**c1**), three items of goods (**c2**), and nine items of goods (**c3**) when $\left[\kappa_{\mathrm{o},\{i,j\}}\right]_{3\times3} = [0.001]_{3\times3}$ W m^{-1} K^{-1} and $\left[\kappa_{\mathrm{pac},\{i,j\}}\right]_{3\times3} = [10]_{3\times3}$ W m^{-1} K^{-1}. **d1-d3,** Temperature distributions of the multi-temperature control system with one item of goods (**d1**), three items of goods (**d2**), and nine items of goods (**d3**) when $\left[\kappa_{\mathrm{o},\{i,j\}}\right]_{3\times3} = [1000]_{3\times3}$ W m^{-1} K^{-1} and $\left[\kappa_{\mathrm{pac},\{i,j\}}\right]_{3\times3} = [10]_{3\times3}$ W m^{-1} K^{-1}. **e1-e3,** Temperature distributions of the multi-temperature control system with one item of goods (**e1**), three items of goods (**e2**), and nine items of goods (**e3**) when $\left[\kappa_{\mathrm{o},\{i,j\}}\right]_{3\times3} = [0.001]_{3\times3}$ W m^{-1} K^{-1} and $\left[\kappa_{\mathrm{pac},\{i,j\}}\right]_{3\times3} = [1000]_{3\times3}$ W m^{-1} K^{-1}. **f1-f3,** Minimum/average/maximum temperature of the goods in the temperature control zones with different numbers when $\left[\kappa_{\mathrm{o},\{i,j\}}\right]_{3\times3} = [0.001]_{3\times3}$ W m^{-1} K^{-1} & $\left[\kappa_{\mathrm{pac},\{i,j\}}\right]_{3\times3} = [10]_{3\times3}$ W m^{-1} K^{-1} (**f1**), $\left[\kappa_{\mathrm{o},\{i,j\}}\right]_{3\times3} = [1000]_{3\times3}$ W m^{-1} K^{-1} & $\left[\kappa_{\mathrm{pac},\{i,j\}}\right]_{3\times3} = [10]_{3\times3}$ W m^{-1} K^{-1} (**f2**), and $\left[\kappa_{\mathrm{o},\{i,j\}}\right]_{3\times3} = [0.001]_{3\times3}$ W m^{-1} K^{-1} & $\left[\kappa_{\mathrm{pac},\{i,j\}}\right]_{3\times3} = [1000]_{3\times3}$ W m^{-1} K^{-1} (**f3**). The remaining thermal parameters were the same as those in Supplementary Fig. 5a. Source data are provided as a Source Data file.

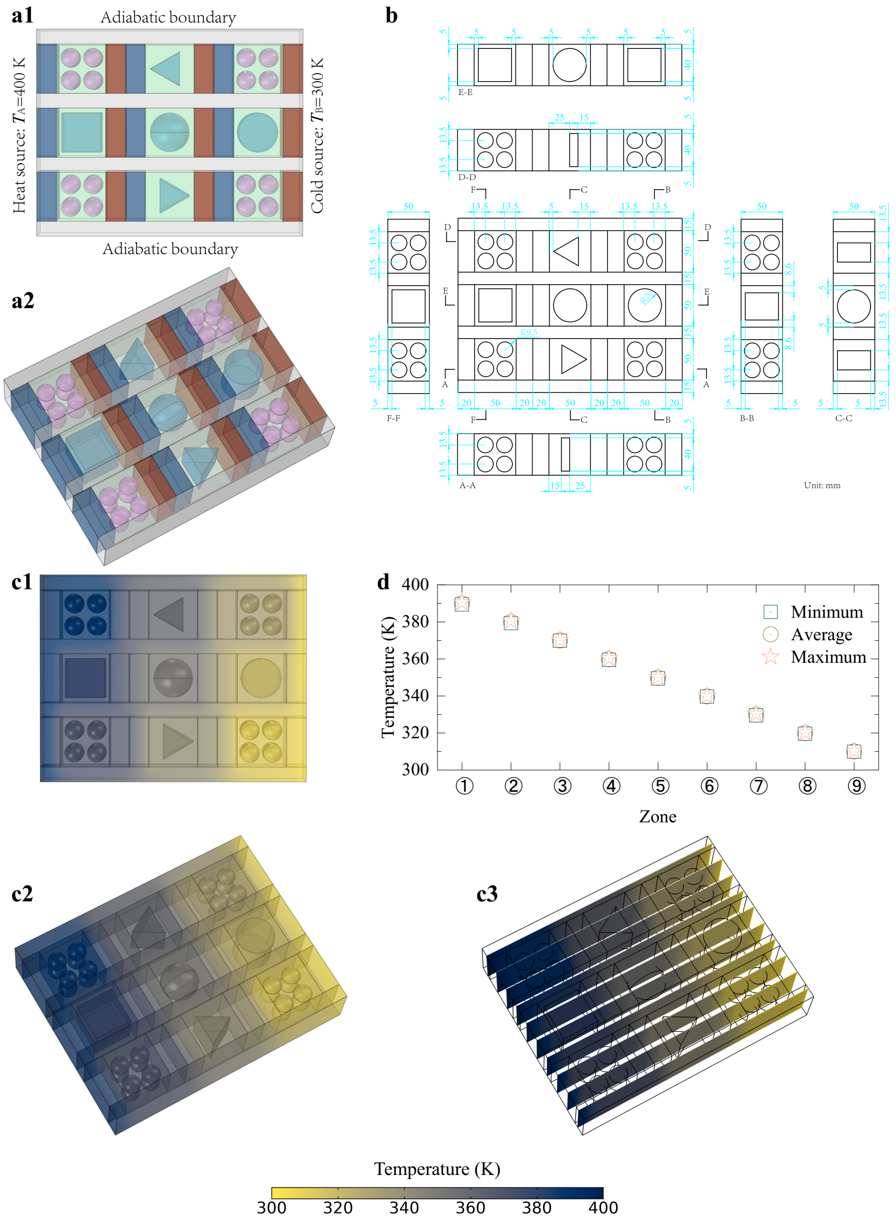

Supplementary Fig. 11|Multi-temperature control performance of a 3-D multi-temperature control system for storing various goods. a1,a2, Perspective geometry in the top (**a1**) and side (**a2**) views of a 3-D multi-temperature control system. We placed eight liquid spheres with $\kappa_{\mathrm{o},\{1,1\}} = 0.5$ W m^{-1} K^{-1} in zone ①, one solid cube with $\kappa_{\mathrm{o},\{2,1\}} = 1.0$ W m^{-1} K^{-1} in zone ②, eight liquid spheres with $\kappa_{\mathrm{o},\{3,1\}} = 1.5$ W m^{-1} K^{-1} in zone ③, one solid triangular prism with $\kappa_{\mathrm{o},\{1,2\}} = 2.0$ W m^{-1} K^{-1} in zone ④, one solid sphere with $\kappa_{\mathrm{o},\{2,2\}} = 2.5$ W m^{-1} K^{-1} in zone ⑤, one solid triangular prism with $\kappa_{\mathrm{o},\{3,2\}} = 3.0$ W m^{-1} K^{-1} in zone ⑥, eight liquid spheres with $\kappa_{\mathrm{o},\{1,3\}} = 3.5$ W m^{-1} K^{-1} in zone ⑦, one solid cylinder with $\kappa_{\mathrm{o},\{2,3\}} = 4.0$ W m^{-1} K^{-1} in zone ⑧, eight liquid spheres with $\kappa_{\mathrm{o},\{3,3\}} = 4.5$ W m^{-1} K^{-1} in zone ⑨. **b,** Structural parameters of the 3-D multi-temperature control system. **c1-c2,** Perspective temperature distributions of the multi-temperature control system in the top (**c1**) and side (**c2**) views under steady-state. **c3,** Cross-section temperature distribution of the multi-temperature control system under steady-state. **d,** Minimum/average/maximum temperature of the goods stored in each temperature control zone under steady-state. **Simulation details:** The density, heat capacity, and dynamic viscosity of the goods stored in the temperature control zone were 100 kg m^{-3}, 100 J kg^{-1} K^{-1}, and 0.001 Pa s, respectively. The remaining thermal parameters were identical to those in Supplementary Fig. 5a. We used "convection-enhanced thermal conductivity" in COMSOL Multiphysics[29] to simulate the goods in fluid states. For simplicity, spheres were approximated as cubes (vertical rectangular cavity model). Cavity height and board distance were both 0.019 m. Source data are provided as a Source Data file.

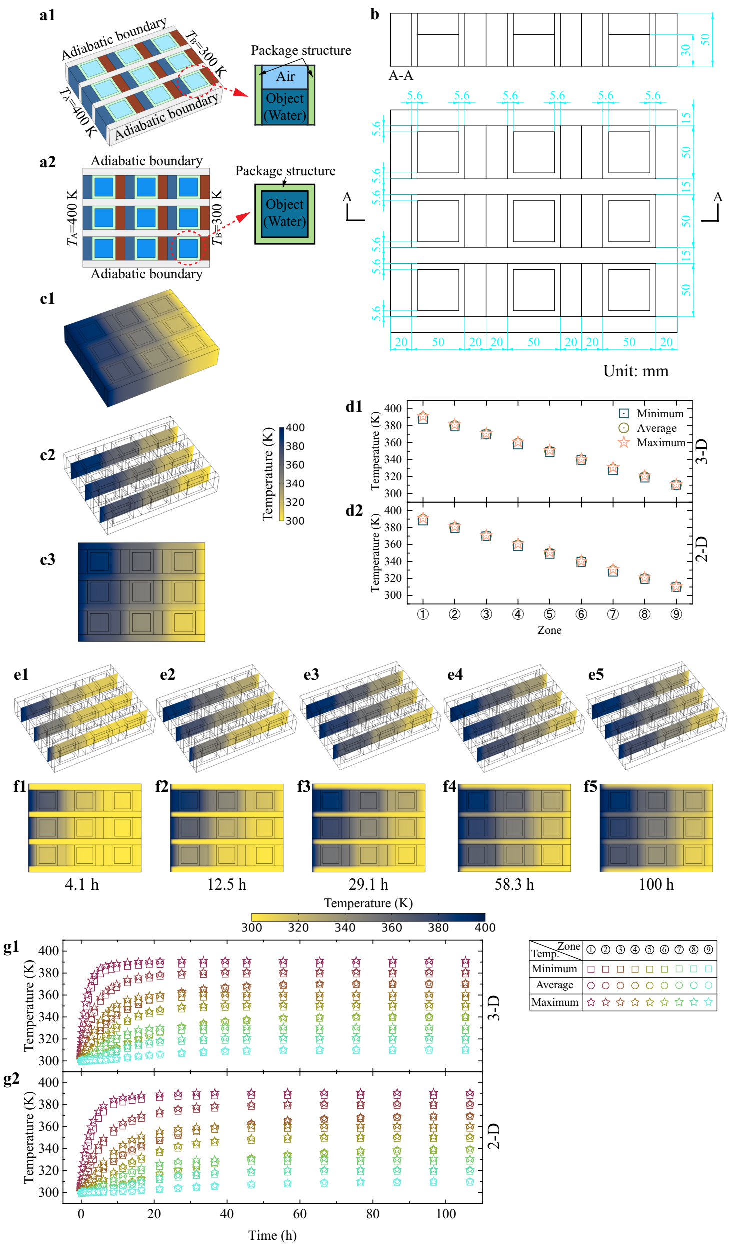

Supplementary Fig. 12|Multi-temperature control performance of a 3-D and a 2-D multi-temperature control system for practical application. **a1,a2,** Geometries of a 3-D (**a1**) and a 2-D (**a2**) multi-temperature control system. The top and ground boundaries of the 3-D multi-temperature control system are adiabatic. **b,** Structural parameters of the multi-temperature control systems. **c1,c2,** Temperature distributions of the 3-D multi-temperature control system on the surface (**c1**) and in cross section (**c2**) under steady-state. **c3,** Temperature distribution of the 2-D multi-temperature control system. **d1,d2,** Minimum/average/maximum temperature of the goods in each temperature control zone under steady-state for 3-D (**d1**) and 2-D (**d2**) multi-temperature control systems. **e1-e5,** Cross-sectional temperature distributions of the 3-D multi-temperature control system under transient-state at characteristic times: 4.1 h (**e1**), 12.5 h (**e2**), 29.1 h (**e3**), 58.3 h (**e4**), and 100 h (**e5**). **f1-f5,** Temperature distributions of the 2-D multi-temperature control system under transient-state at characteristic times: 4.1 h (**f1**), 12.5 h (**f2**), 29.1 h (**f3**), 58.3 h (**f4**), and 100 h (**f5**). **g1,g2,** Minimum/average/maximum temperature variations of the goods in each temperature control zone under transient-state for 3-D (**g1**) and 2-D (**g2**) multi-temperature control system. **Simulation details:** The initial temperature of the systems was 300 K. The thermal conductivity, density, specific heat capacity, dynamic viscosity, and specific heat rate of the goods were $0.599\ \mathrm{W\ m^{-1}\ K^{-1}}$, $998.2\ \mathrm{kg\ m^{-3}}$, $4183\ \mathrm{J\ kg^{-1}\ K^{-1}}$, $0.001\ \mathrm{Pa\ s}$, and 1, respectively[30]. The thermal conductivity, density, specific heat capacity, dynamic viscosity, and specific heat rate of the air were $0.0259\ \mathrm{W\ m^{-1}\ K^{-1}}$, $1.205\ \mathrm{kg\ m^{-3}}$, $1005\ \mathrm{J\ kg^{-1}\ K^{-1}}$, $0.00002\ \mathrm{Pa\ s}$, and 1.4, respectively[30]. The remaining thermal parameters were consistent with those in Supplementary Fig. 5a. We used "convection-enhanced thermal conductivity" in COMSOL Multiphysics[29] to simulate the water and the air using vertical rectangular cavity models. For the water, the cavity height and board distance were $0.03\ \mathrm{m}$ and $0.0387\ \mathrm{m}$, respectively. For the air, they were $0.02\ \mathrm{m}$ and $0.0387\ \mathrm{m}$, respectively. Source data are provided as a Source Data file.

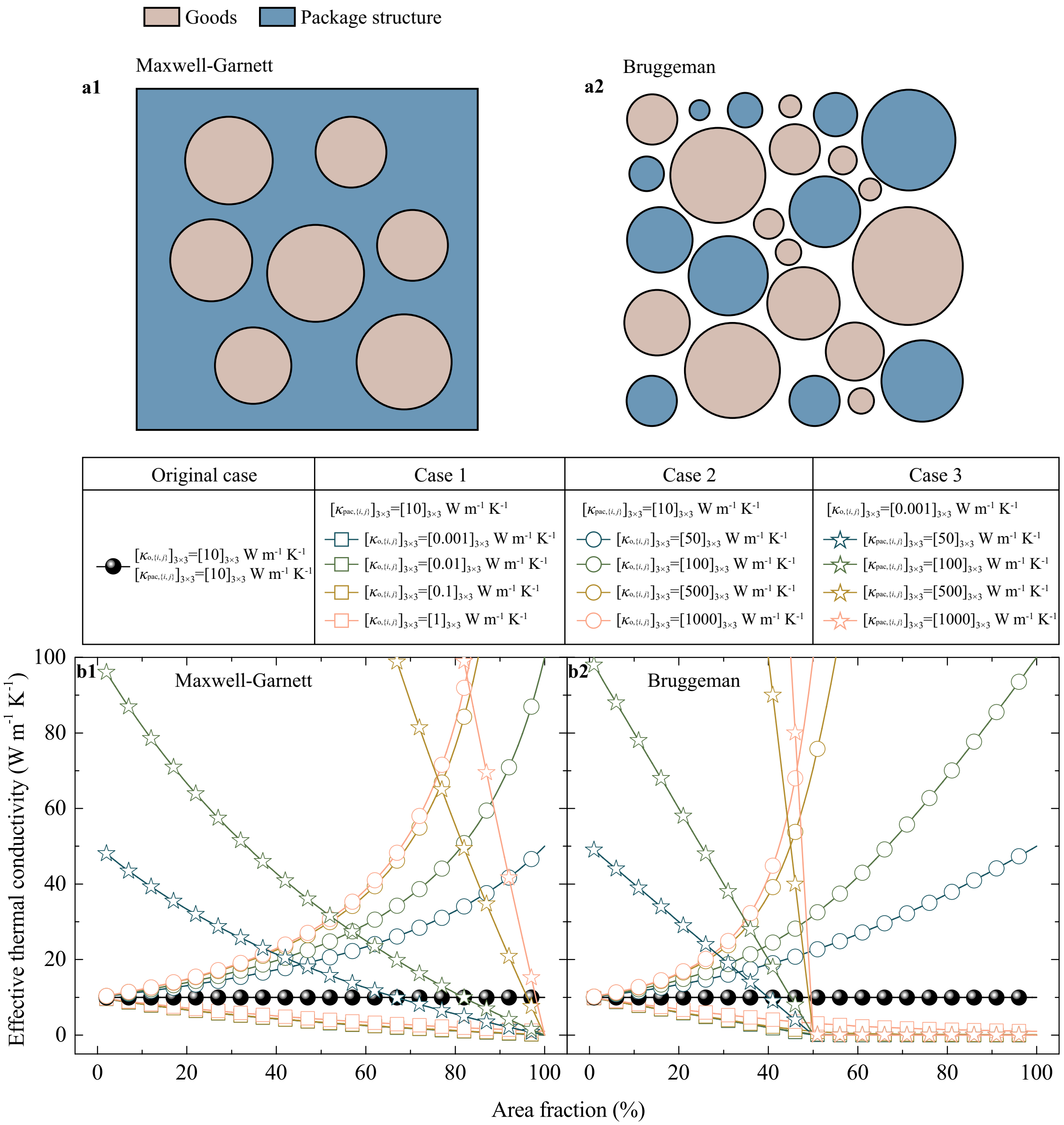

Supplementary Fig. 13|Effective medium approximation. **a1,a2,** Distribution of goods and package structures for the Maxwell-Garnett approximation (**a1**) and the Bruggeman approximation (**a2**) [5–7]. **b1,b2,** Effective thermal conductivities of the temperature control zone under different cases, calculated using the Maxwell-Garnett approximation (**b1**) and Bruggeman approximation (**b2**). Please note that we take $\kappa_{\mathrm{pac},\{i,j\}} = \kappa_{\mathrm{o},\{i,j\}} = 10$ W m^{-1} K^{-1} as a reference. Source data are provided as a Source Data file.

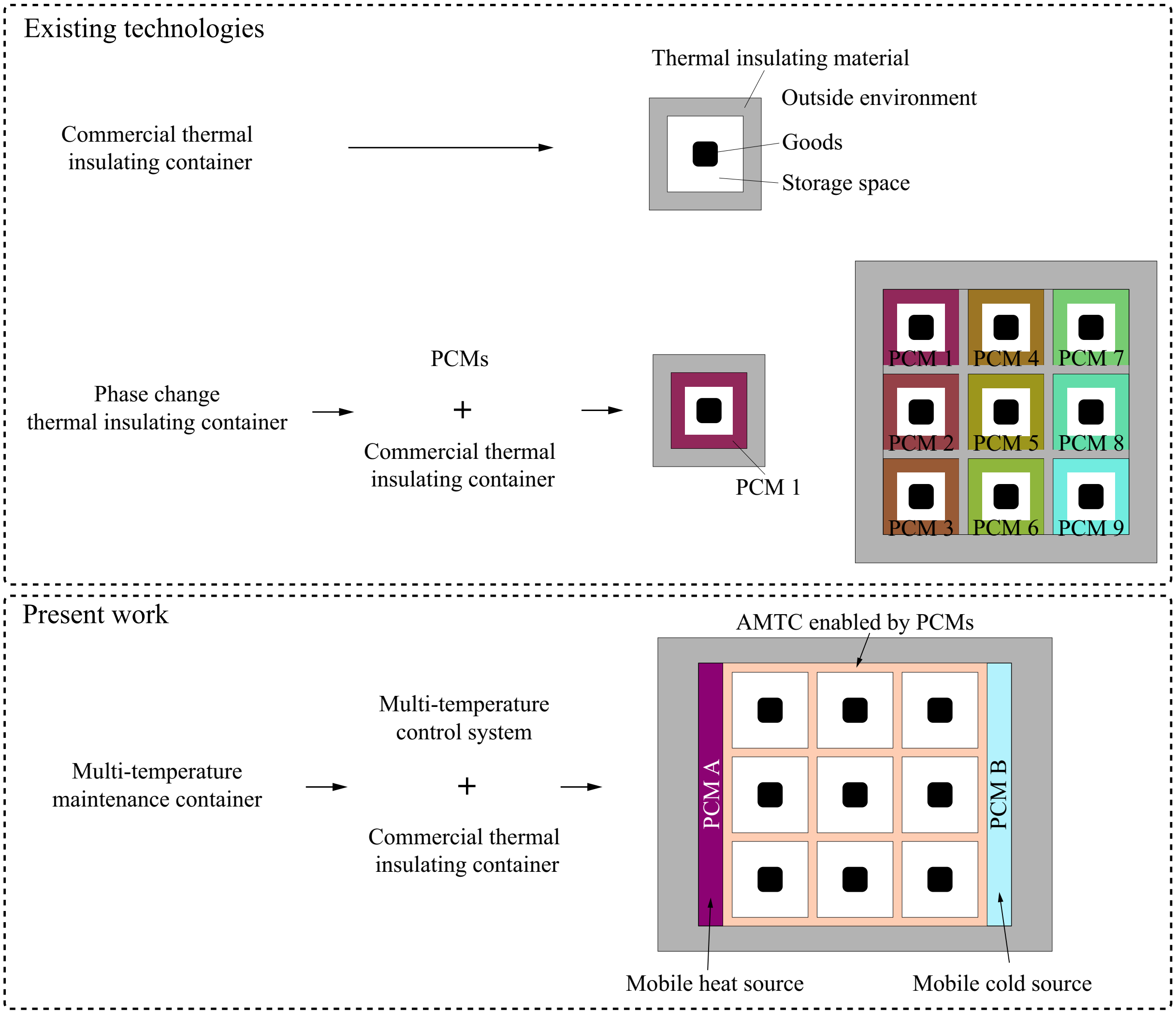

Supplementary Fig. 14|Schematic diagram for a commercial thermal insulating container, a phase change thermal insulating container, and a multi-temperature maintenance container. Abbreviation: PCM, phase change material.

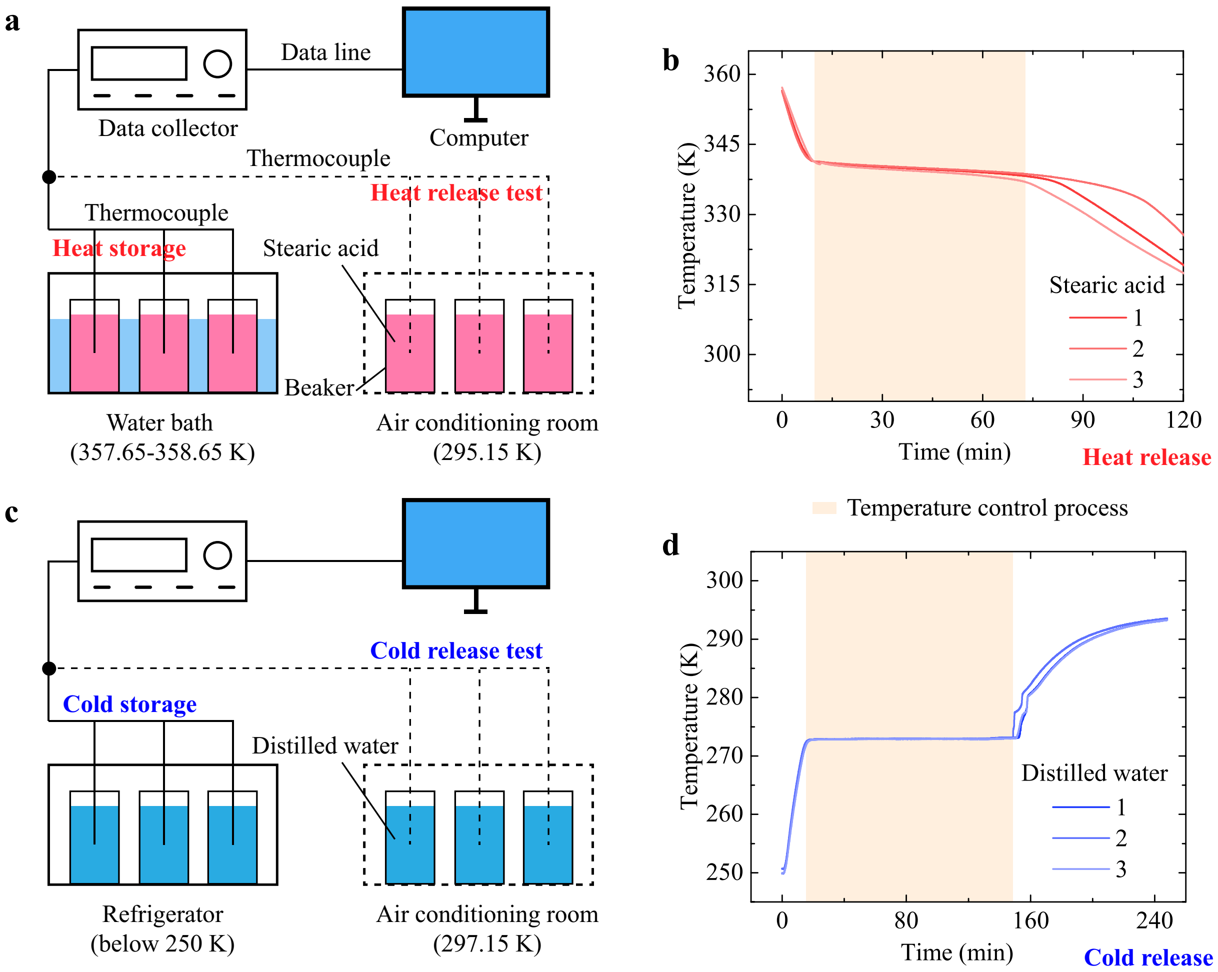

Supplementary Fig. 15|Experimental setup for heat and cold release performances of PCM A and PCM B. a,b, Heat storage/release experiment (**a**) and heat release performance (**b**) for stearic acid. **c,d,** Cold storage/release experiment (**c**) and cold release performance (**d**) for distilled water. The temperature control processes were employed to provide the temperature difference for the multi-temperature control system. Source data are provided as a Source Data file.

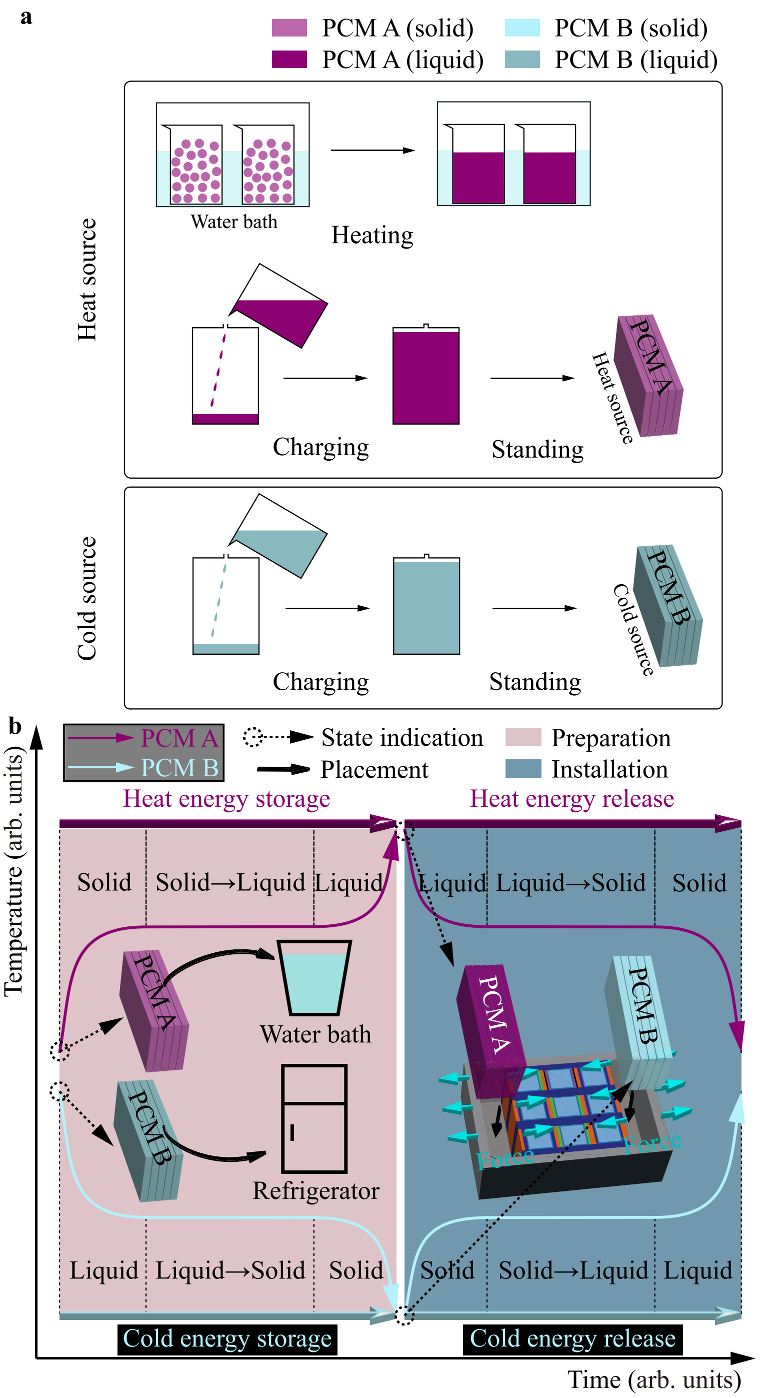

Supplementary Fig. 16|Fabricating, preparation, and installation of heat and cold sources. **a,** Fabrication of the mobile heat and cold sources. **b,** Preparation and installation of the mobile heat and cold sources. **Abbreviations:** PCM, phase change material. arb., arbitrary.

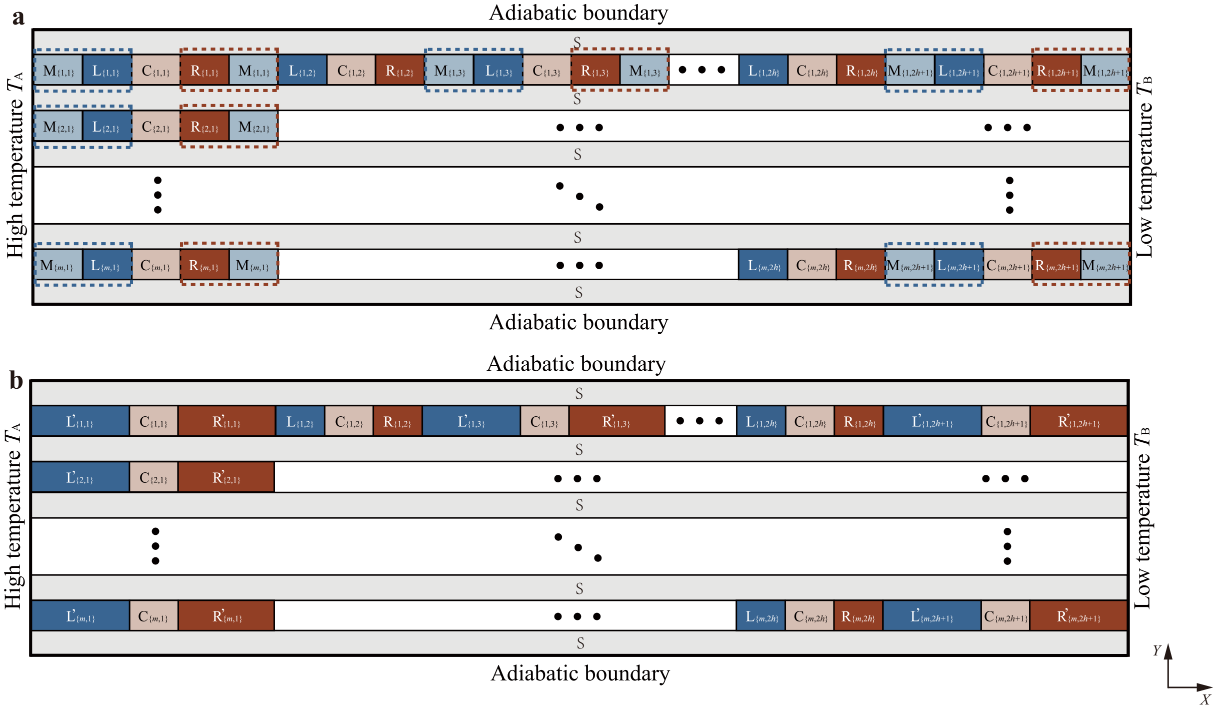

Supplementary Fig. 17|Schematic diagram for the design of an actual multi-temperature control system. Multi-temperature control system comprising five (**a**) and four (**b**) types of zones. Take $n\ (= 2h + 1, h = 0, 1, 2 \cdots)$, which is odd, as an example.

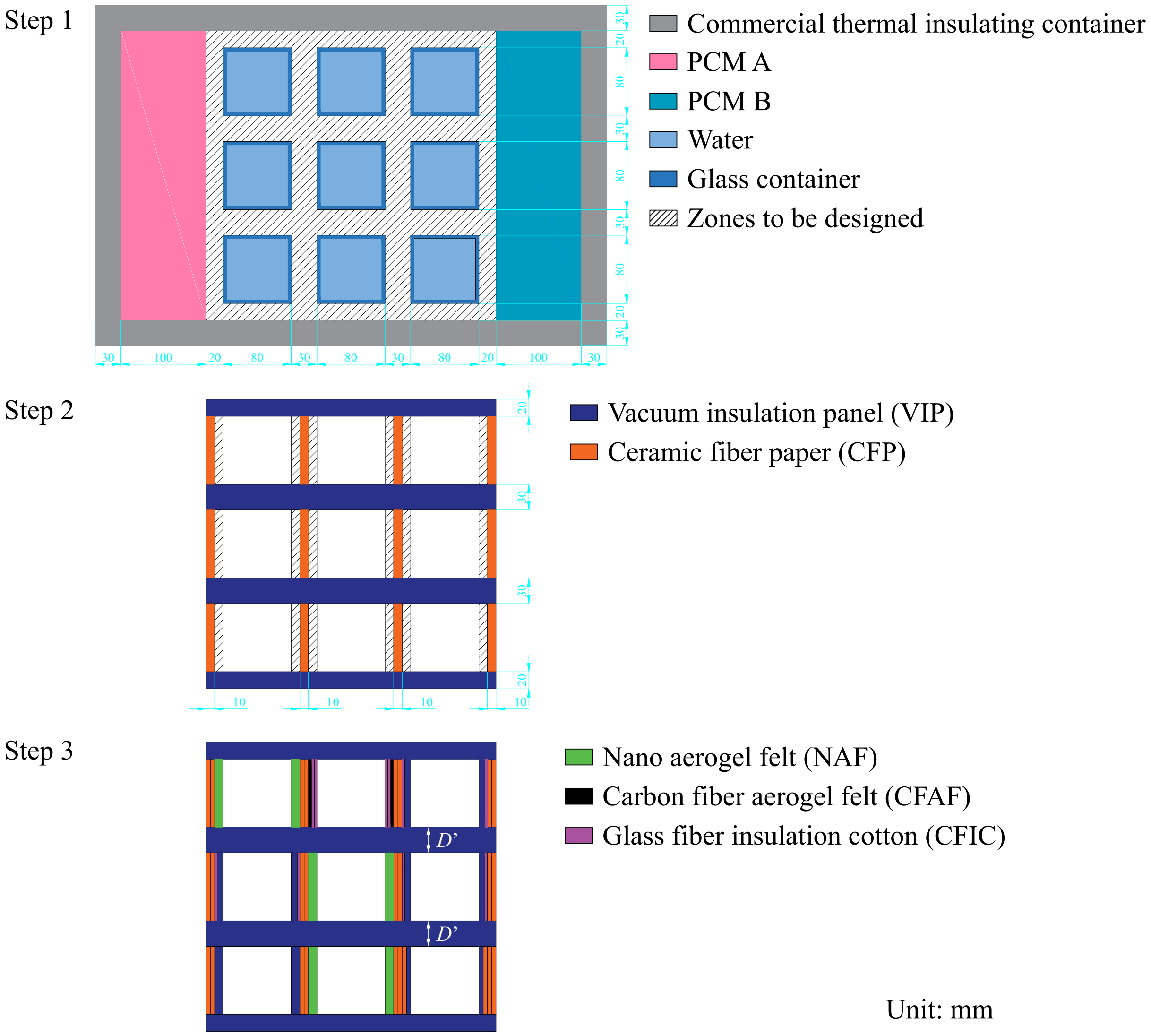

Supplementary Fig. 18|Design steps for an actual multi-temperature control system. Notes: For the experiment, the volume of PCM A (PCM B) and the thickness D' of two VIPs in the middle do not strictly correspond to the above designing parameters due to dimensional errors, installation of heat/cold source, and volume expansion of PCMs. The experimental devices are shown in Fig. 5b. This discrepancy does not impact the experiments, as PCM A (PCM B) still functions as a mobile heat (cold) source, and the VIPs still serve as thermal insulating materials, aligning with the principle of AMTC. **Abbreviation:** PCM, phase change material.

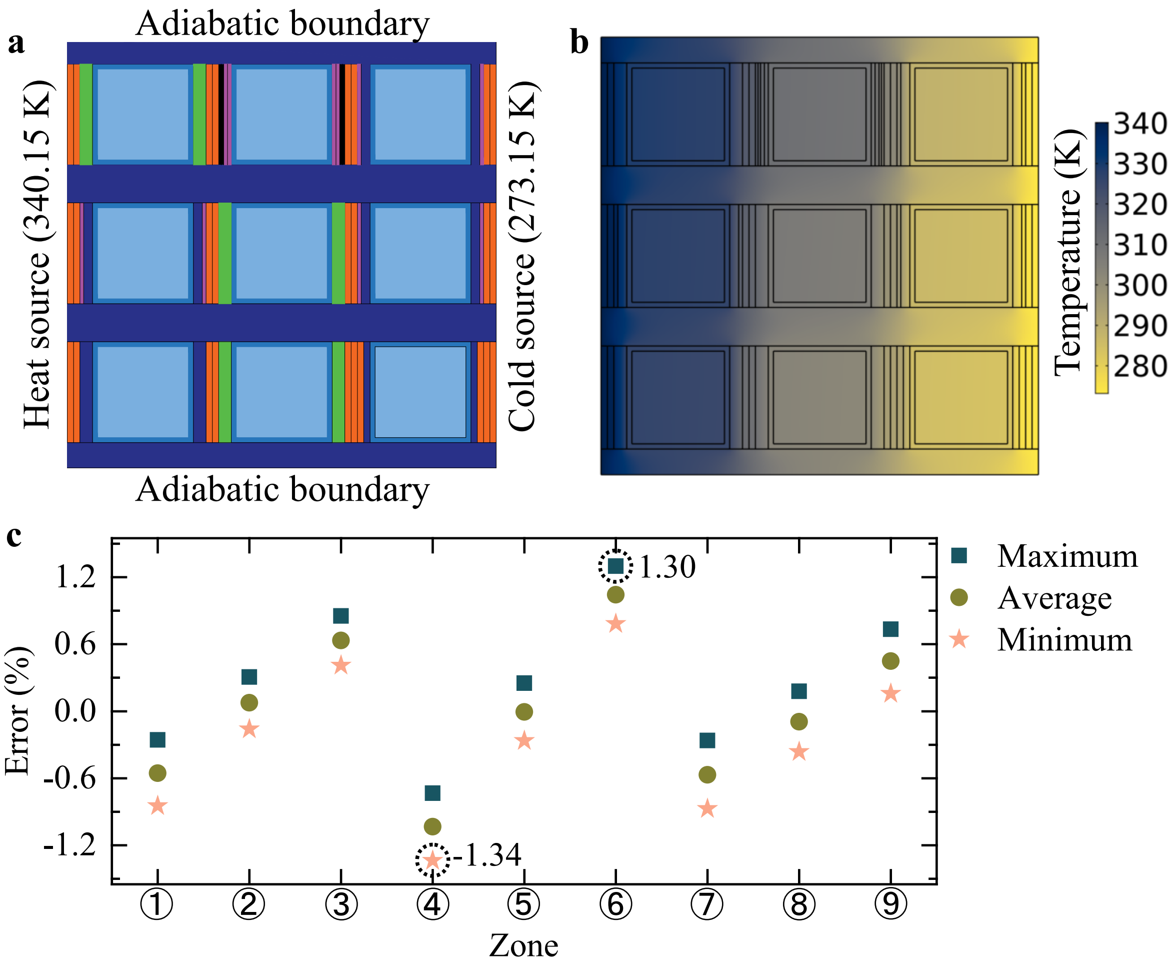

Supplementary Fig. 19|Validation of the multi-temperature control performance of the actual multi-temperature control system. a, Schematic diagram of the actual multi-temperature control system. **b,** Temperature distribution of the multi-temperature control system in steady-state. **c,** Discrepancies between the finite element simulation results and the required values. **Simulation details:** The thermal conductivity, density, specific heat capacity, dynamic viscosity, and specific heat rate of the simulacra (water) were $0.618\ \mathrm{W\ m^{-1}\ K^{-1}}$, $995.7\ \mathrm{kg\ m^{-3}}$, $4174\ \mathrm{J\ kg^{-1}\ K^{-1}}$, 0.0008 Pa s, and 1, respectively[30, 31]. We used "convection-enhanced thermal conductivity" in COMSOL Multiphysics[29] to simulate the water (vertical rectangular cavity models). The cavity height and board distance for the water were $0.001\ \mathrm{m}$ and $0.08\ \mathrm{m}$, respectively. For further thermal/structural parameters of the multi-temperature control system, see Supplementary Fig. 18 and Supplementary Table 4. Source data are provided as a Source Data file.

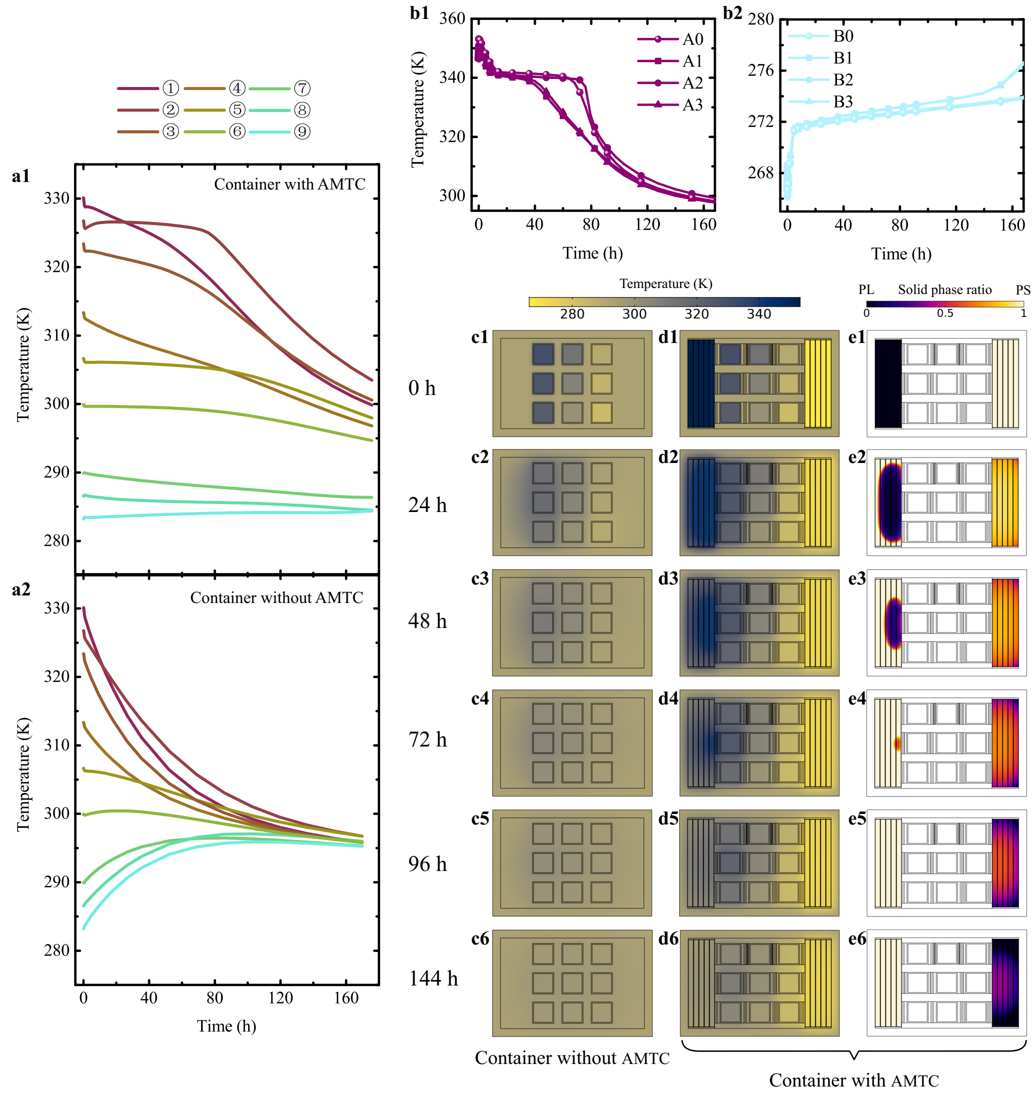

Supplementary Fig. 20|Multi-temperature maintenance performance (finite element simulation) of a 2-D multi-temperature control system. a1,a2, Multi-temperature variations of the goods stored in the multi-temperature maintenance container (container with AMTC, **a1**) and the commercial thermal insulating container (container without AMTC, **a2**). **b1,b2,** Temperature variations at test points A0-A3 (**b1**) and B0-B3 (**b2**). The positions of the test points are shown in Fig. 5c. **c1-c6,** Temperature distributions of the commercial thermal insulating container at characteristic times: 0 h (**c1**), 24 h (**c2**), 48 h (**c3**), 72 h (**c4**), 96 h (**c5**), and 144 h (**c6**). **d1-d6,** Temperature distributions of the multi-temperature maintenance container at characteristic times: 0 h (**d1**), 24 h (**d2**), 48 h (**d3**), 72 h (**d4**), 96 h (**d5**), and 144 h (**d6**). **e1-e6,** Phase distributions of the multi-temperature maintenance container (PCM A & PCM B) at characteristic times: 0 h (**e1**), 24 h (**e2**), 48 h (**e3**), 72 h (**e4**), 96 h (**e5**), and 144 h (**e6**). **Simulation details:** The initial temperature of the PCM A (PCM B) was 353.15 K (266.15 K). The initial temperature of each glass of water (simulacrum) was indicated in Eq. (2). The initial temperature of the remaining components of the container was 293.15 K. The convective heat transfer coefficient between the containers and the exterior environment was 10 $\mathrm{W\,m^{-2}\,K^{-1}}$. The environmental temperature stood at 293.15 K. The convective heat transfer inside the container was neglected. The thermal conductivities of the commercial thermal insulating container, high-density polyethylene, water, glass, and air were 0.04 $\mathrm{W\,m^{-1}\,K^{-1}}$, 0.5 $\mathrm{W\,m^{-1}\,K^{-1}}$, 0.6 $\mathrm{W\,m^{-1}\,K^{-1}}$, 0.875 $\mathrm{W\,m^{-1}\,K^{-1}}$, and 0.0259 $\mathrm{W\,m^{-1}\,K^{-1}}$, respectively[30–32]. The density and specific heat capacity of the water were 1000 $\mathrm{kg\,m^{-3}}$ and 4200 $\mathrm{J\,kg^{-1}\,K^{-1}}$, respectively. The specific heat ratio of the water was 1. For additional thermal conductivities of the multi-temperature control system components, refer to Supplementary Fig. 18 and Supplementary Table 3. We used "phase change material" in COMSOL Multiphysics[29] to simulate the phase transitions of the PCM A & PCM B. The transition interval was 5 K. The thermal properties of the PCMs can be found in Supplementary Table 2. Excluding the PCMs and water, we set the density and the specific heat capacity of the remaining components to 100 $\mathrm{kg\,m^{-3}}$ and 420 $\mathrm{J\,kg^{-1}\,K^{-1}}$ for simplicity. Source data are provided as a Source Data file. **Abbreviations:** AMTC, adaptive multi-temperature control. PL, pure liquid. PS, pure solid.

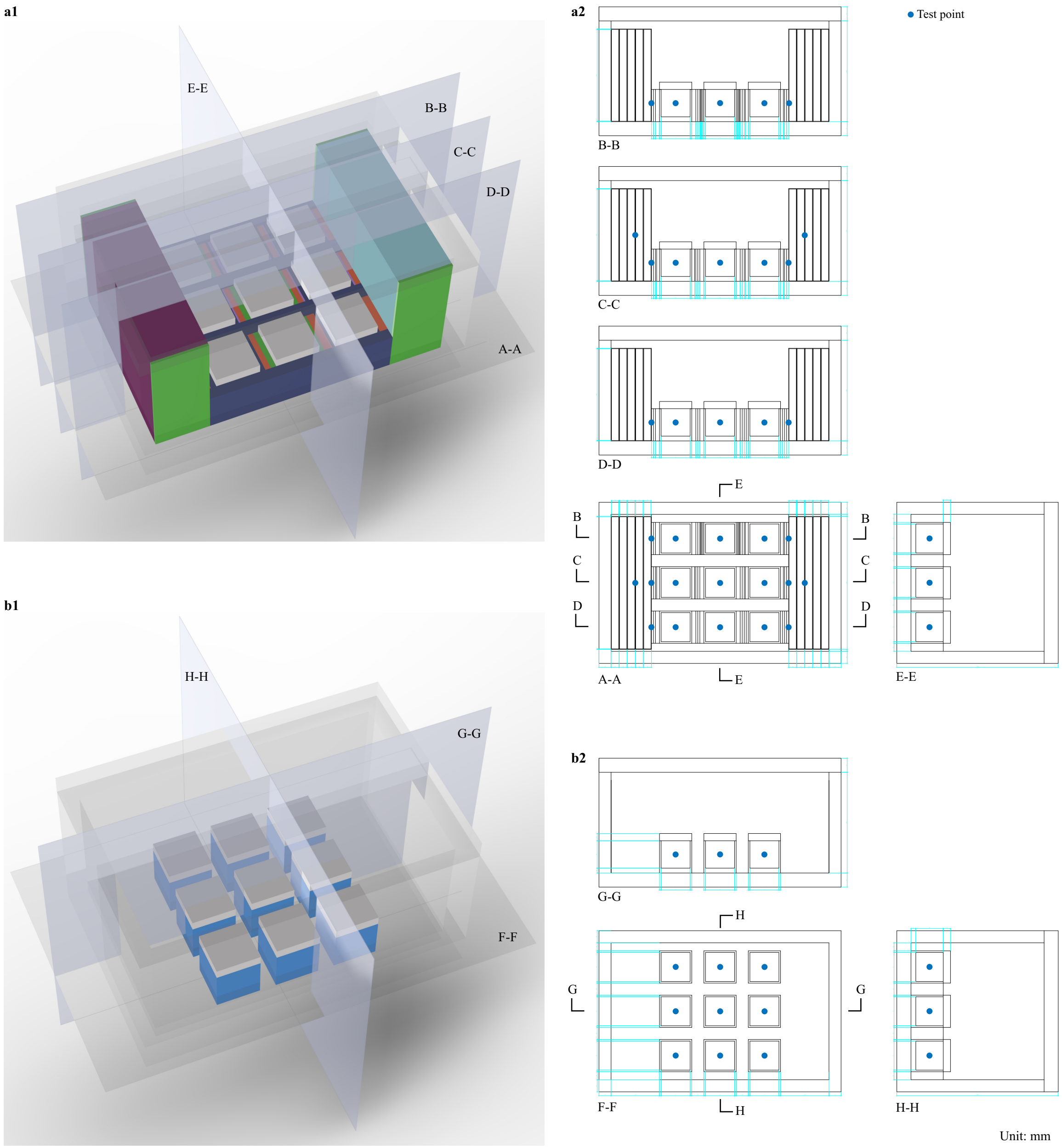

Supplementary Fig. 21|Schematic of a 3-D multi-temperature maintenance container and a 3-D commercial thermal insulating container. a1,a2, Cross-section view (**a1**) and structural parameters (**a2**) of a 3-D multi-temperature maintenance container. **b1,b2,** Cross-section view (**b1**) and structural parameters (**b2**) of a 3-D commercial thermal insulating container. The reference test points for thermocouples are indicated in **a2** and **b2**.

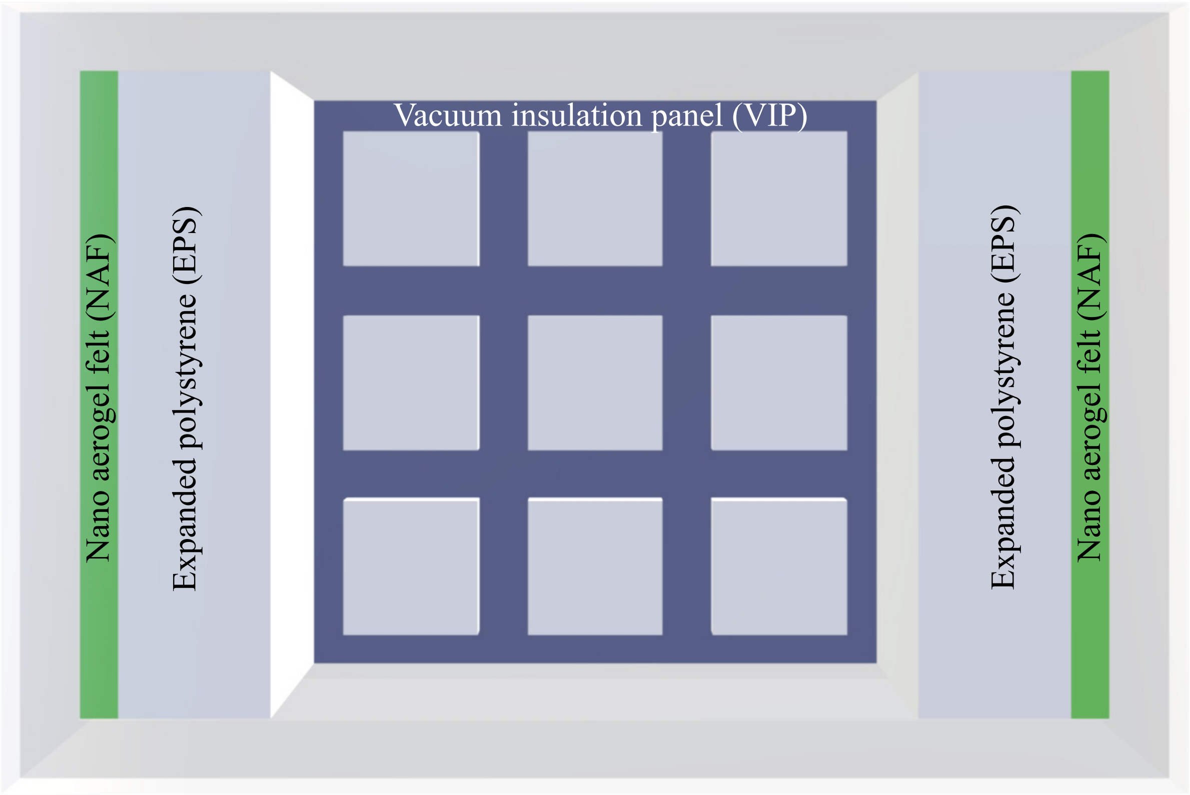

Supplementary Fig. 22|Schematic diagram for the container with LTCM. The height of the low thermal conductivity material (LTCM), in this instance, used to replace the mobile heat and cold sources in the multi-temperature maintenance container, was adjusted to 245 mm. All other parameters remained unchanged. For further details, please refer to Supplementary Fig. 21a.

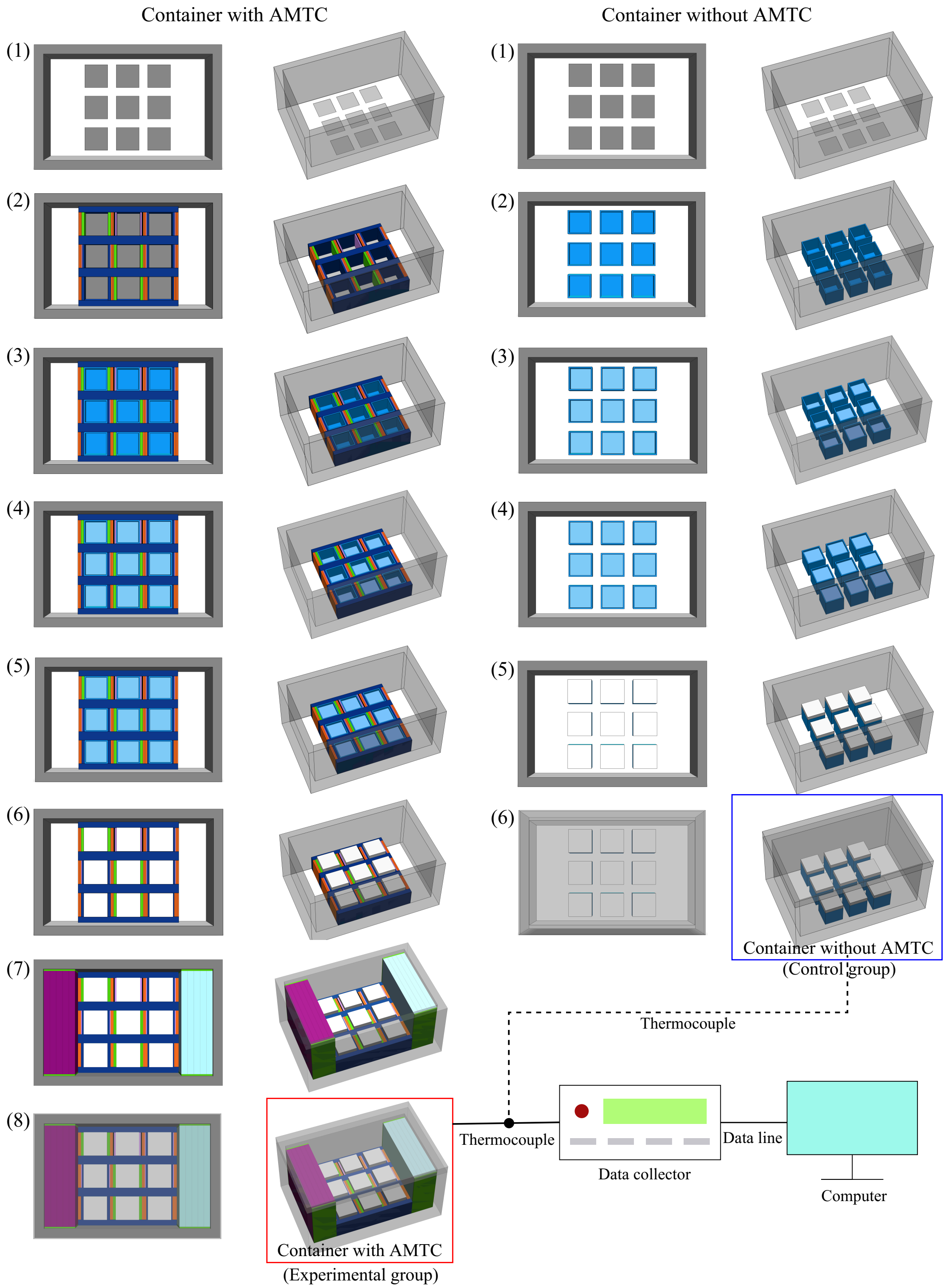

Supplementary Fig. 23|Installation procedures for a multi-temperature maintenance container (container with AMTC) and a commercial thermal insulating container (container without AMTC) for multi-temperature maintenance testing. For the reference positions of temperature test points, please see Supplementary Fig. 21. **Abbreviation:** AMTC, adaptive multi-temperature control.

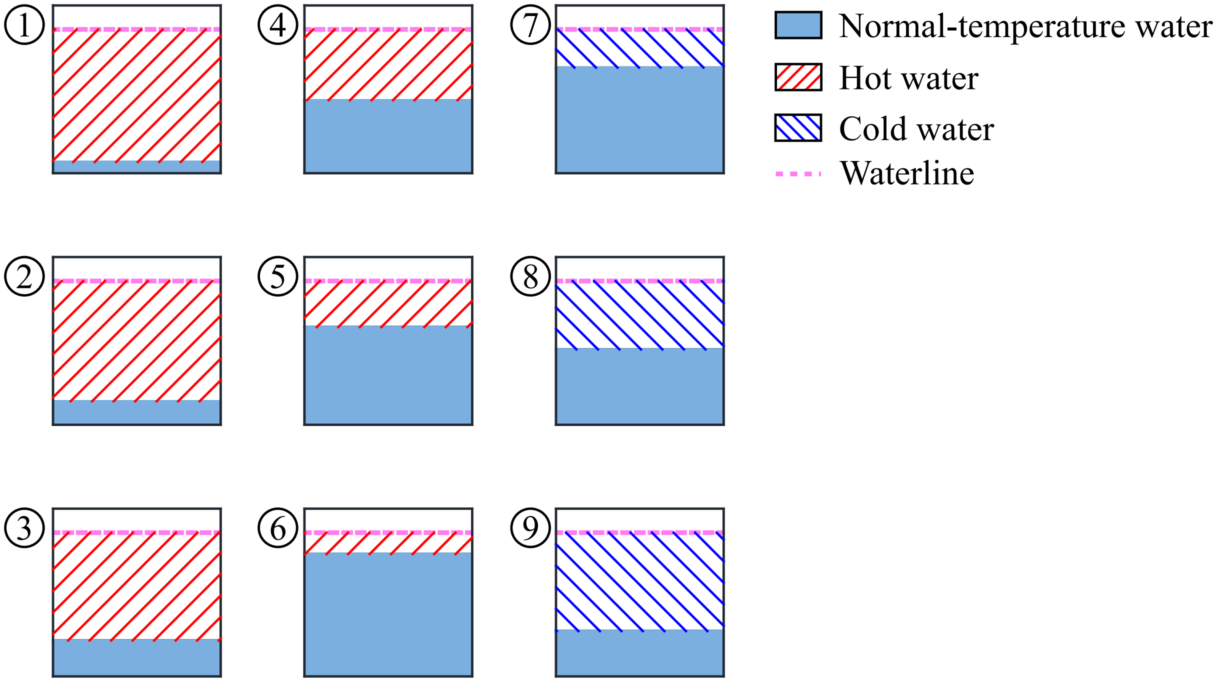

Supplementary Fig. 24|Schematic illustrating the preparation of water at different initial temperatures. The waterline represents a volume of 300 mL. For further discussions, we labelled each piece of glassware (and the goods stored within) as ①, ②· · · ⑨ (Methods).

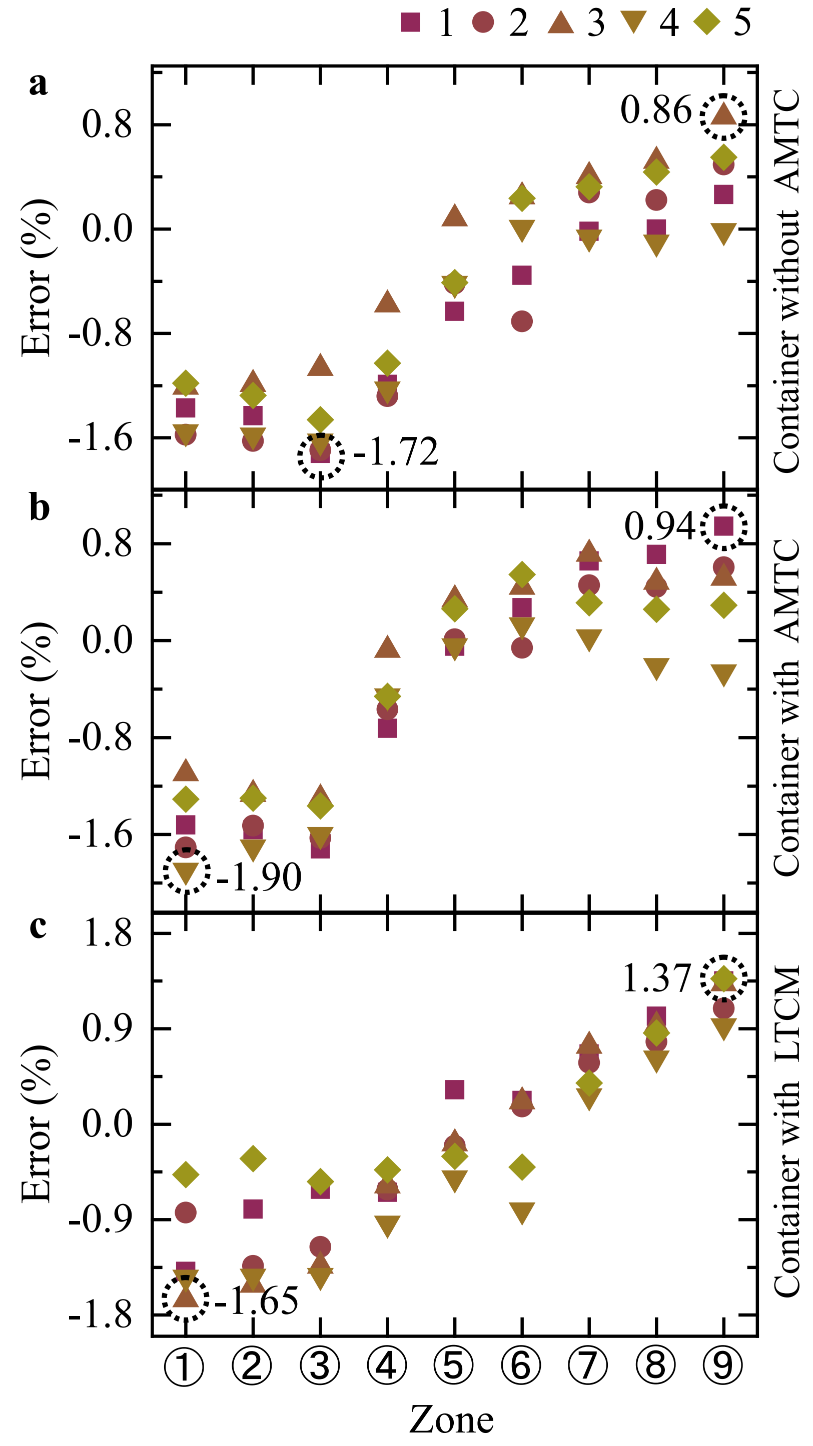

Supplementary Fig. 25|Validation of the preparation methods for water (simulacra) with different initial temperatures. Errors in the initial temperatures for each glass of water (representative of goods) in the commercial thermal insulating container 1 (container without AMTC, **a**), multi-temperature maintenance container (container with AMTC, **b**), and commercial thermal insulating container 2 (container with LTCM, **c**) across five independent tests. Source data are provided as a Source Data file. **Abbreviations:** AMTC, adaptive multi-temperature control. LTCM, low thermal conductivity material.

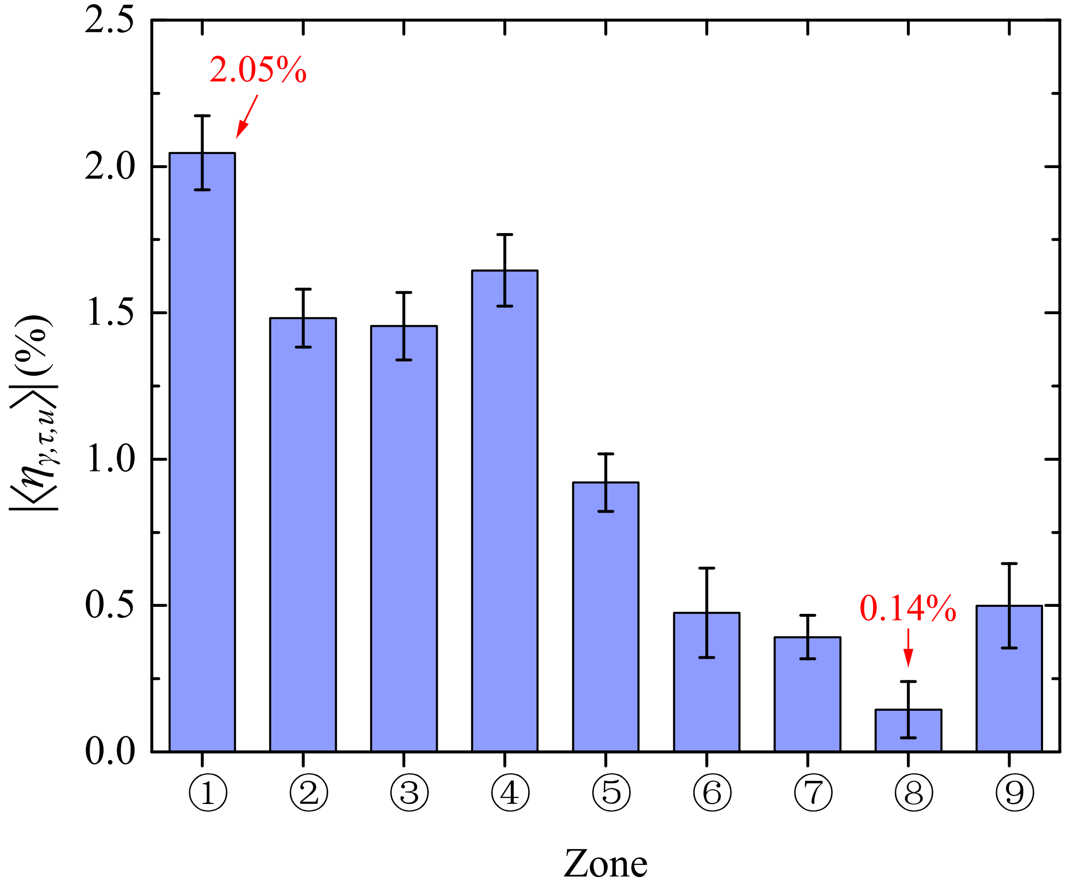

Supplementary Fig. 26|Temperature variation magnitudes $|\langle\eta_{\gamma,\tau,u}\rangle|$ of the simulacra after two hours. The foundational data was extracted from Fig. 7c at two hours corresponding to the container with AMTC ($\tau = 2$ h and $u = 1$; Methods). The lowest value, 0.14%, is noted in zone ⑧, whilst the highest value, 2.05%, is seen in zone ①. Source data are provided as a Source Data file.

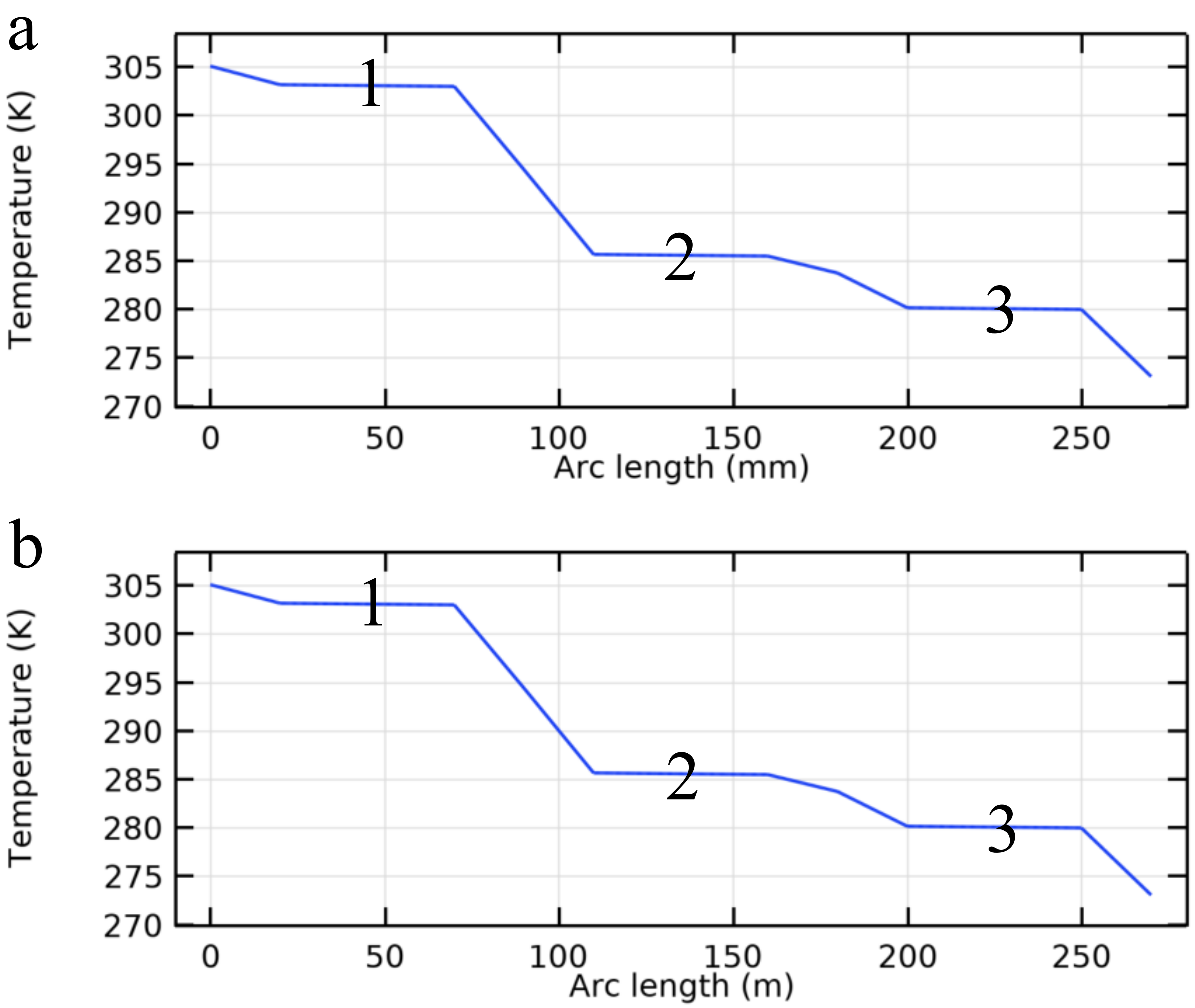

Supplementary Fig. 27|Temperature distribution on the characteristic lines for the case in Fig. 3a. a, pertains to a small size, whilst **b,** is associated with a large size.

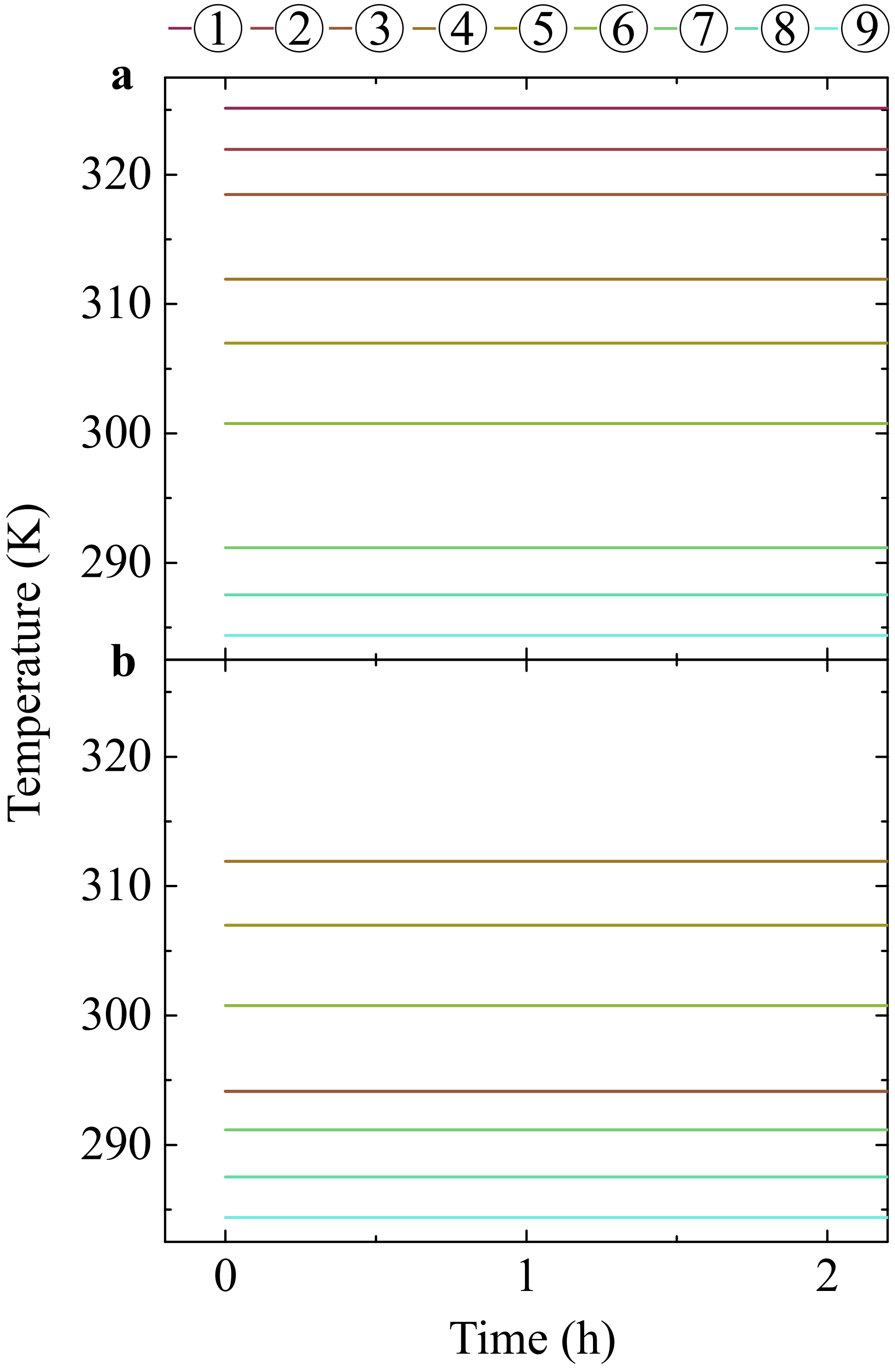

Supplementary Fig. 28|Temperature variation of each simulacrum in large size cases. Source data are provided as a Source Data file.

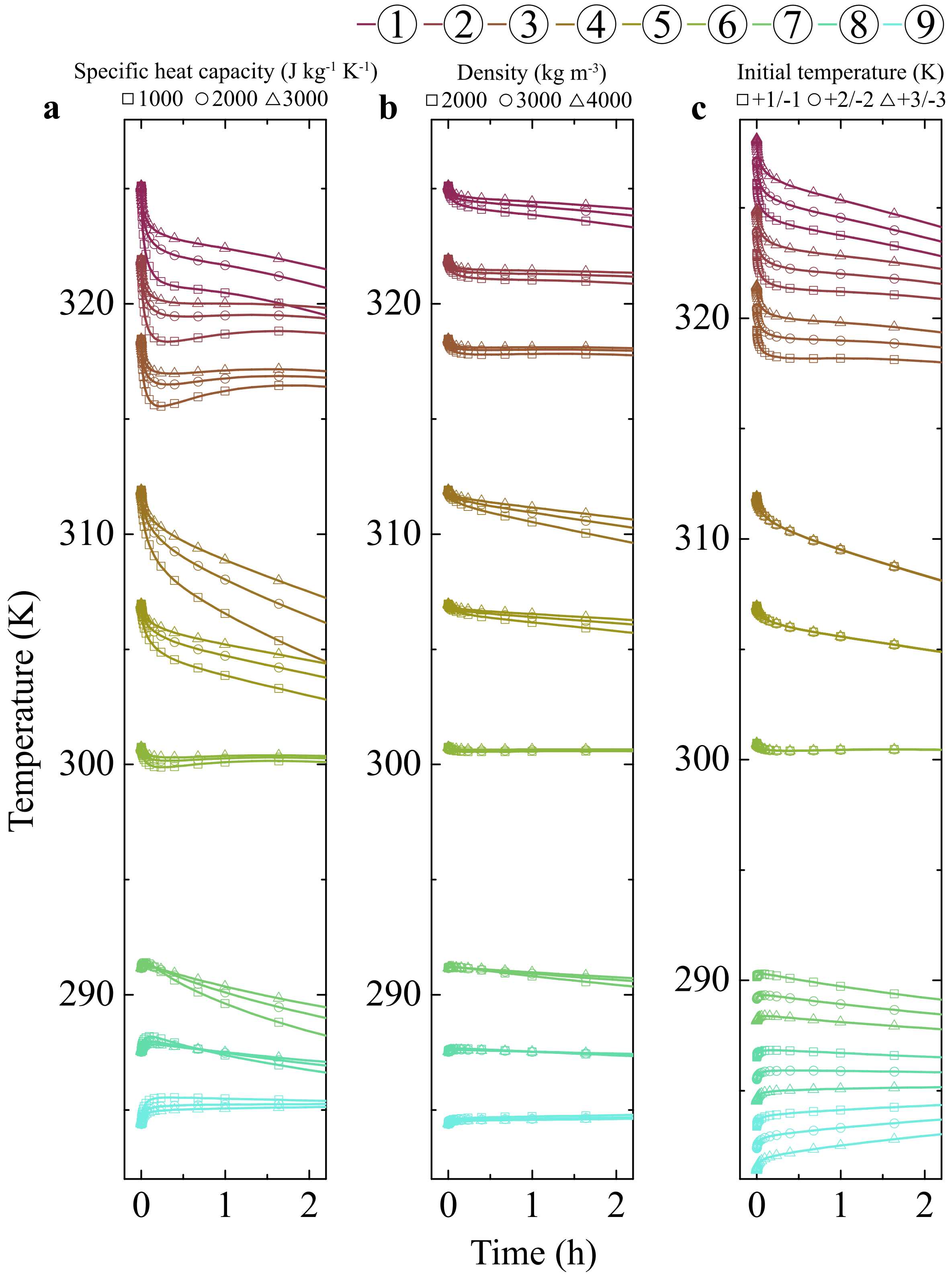

Supplementary Fig. 29|Thermal inertia research for the multi-temperature maintenance container. Effect of specific heat capacity (**a**), density (**b**), and initial temperature (**c**) on the temperature-time curves of each simulacrum. In **c**, the symbol +x/-x indicates that the initial temperatures of the simulacra in the high-temperature regions were increased by x K and those in the low-temperature regions were reduced by x K. Source data are provided as a Source Data file.

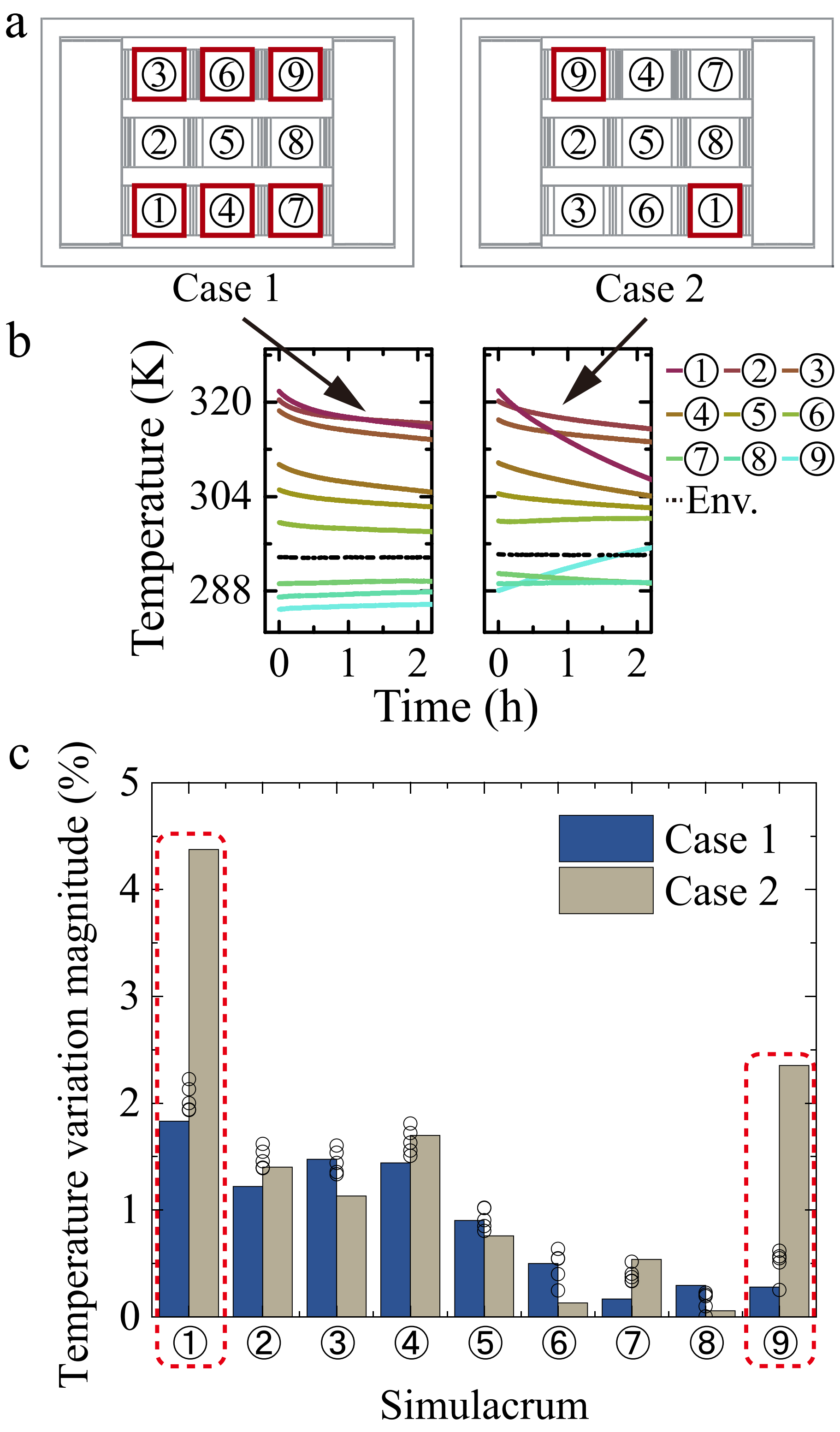

Supplementary Fig. 30|Wrong placement research for the multi-temperature maintenance container. a, Schematic representation of two cases of wrong placement. Misplaced simulacrum is marked with a red box. **b,** Experimental results for the temperature-time curve of the simulacrum in each temperature control zone. **c,** Temperature variation magnitude of each simulacrum after two hours. The five test results with correct placement were marked with circles for reference. Significant disruptions in temperature maintenance are highlighted in red boxes. Source data are provided as a Source Data file.

Supplementary Table 1|Simulation results for the maximum/average/minimum temperature of the goods stored in the temperature control zones using various approaches.

Approach	Temperature (K)	Zone								
		①	②	③	④	⑤	⑥	⑦	⑧	⑨
1	Maximum	389.97	380.12	370.51	359.78	350.10	340.33	329.59	320.07	310.13
	Average	389.92	380.02	370.46	359.73	350.00	340.28	329.54	319.98	310.08
	Minimum	389.87	379.93	370.41	359.67	349.90	340.23	329.49	319.88	310.03
2	Maximum	390.05	380.03	371.21	360.40	350.02	339.10	329.89	320.01	309.63
	Average	390.02	380.01	371.20	360.37	350.00	339.08	329.86	319.99	309.62
	Minimum	389.99	379.99	371.19	360.34	349.98	339.07	329.83	319.97	309.60
3	Maximum	390.06	379.55	370.75	359.99	350.03	340.94	329.92	320.52	310.19
	Average	389.99	379.52	370.72	359.92	350.00	340.91	329.84	320.48	310.16
	Minimum	389.91	379.49	370.69	359.84	349.97	340.88	329.77	320.45	310.13
4	Maximum	389.97	380.08	370.51	359.78	350.10	340.33	329.59	320.11	310.13
	Average	389.92	379.99	370.46	359.73	350.00	340.28	329.54	320.01	310.08
	Minimum	389.87	379.89	370.41	359.67	349.90	340.23	329.49	319.91	310.03
5	Maximum	390.30	380.21	370.03	360.27	350.20	339.87	330.32	320.18	310.10
	Average	390.00	380.01	369.94	359.98	350.00	339.77	330.03	319.99	310.00
	Minimum	389.70	379.82	369.84	359.68	349.80	339.68	329.73	319.79	309.91
6	Maximum	389.97	380.08	370.51	359.78	350.01	340.33	329.59	320.11	310.13
	Average	389.92	379.99	370.46	359.73	350.00	340.28	329.54	320.01	310.08
	Minimum	389.87	379.89	370.41	359.67	349.90	340.23	329.49	319.91	310.03

Supplementary Table 2|Thermal properties of the phase change materials (PCMs) ("/" indicates data not required for finite element simulation)[8, 30, 33–37].

PCMs / Thermal property	Stearic acid (PCM A)	Distilled water (PCM B)
Melting temperature (K)	/	273.15
Solidification temperature (K)	340.15	/
Latent heat of melting $\left(\mathrm{kJ\ kg^{-1}}\right)$	/	333
Latent heat of solidification $\left(\mathrm{kJ\ kg^{-1}}\right)$	219	/
Thermal conductivity of solid $\left(\mathrm{W\ m^{-1}\ K^{-1}}\right)$	0.19	2.20
Thermal conductivity of liquid $\left(\mathrm{W\ m^{-1}\ K^{-1}}\right)$	0.13	0.55
Specific heat capacity of solid $\left(\mathrm{kJ\ kg^{-1}\ K^{-1}}\right)$	2.5	2.0
Specific heat capacity of liquid $\left(\mathrm{kJ\ kg^{-1}\ K^{-1}}\right)$	2.1	4.2
Density of solid $(\mathrm{kg\ m^{-3}})$	953	917
Density of liquid $(\mathrm{kg\ m^{-3}})$	844	1000

Supplementary Table 3|Actual materials for the multi-temperature control system (data not cited are based on suppliers' parameters and material data network[31]).

Material	Thermal conductivity $(\mathrm{W\,m^{-1}\,K^{-1}})$	Density $(\mathrm{kg\,m^{-3}})$	Specific heat capacity $(\mathrm{J\,kg^{-1}\,K^{-1}})$	Suppliers
Ceramic fiber paper (CFP)	0.080	210	900	Shanghai Liangluo Electromechanical Equipment Business Department, China;<br>Zibo Jiuqiang Refractory Co., Ltd., China;<br>Shangdong Redon High Temperature Material Co, Ltd., China.
Nano aerogel felt (NAF)	0.018	200	549[38]	Langfang Yuncun Thermal Insulation Material Co., Ltd., China
Carbon fiber aerogel felt (CFAF)	0.022	180	502	Langfang Yuncun Thermal Insulation Material Co., Ltd., China;<br>MAGICAL New Materials Research, TaoBao, China.
Vacuum insulation panel (VIP)	0.008	220	800	Langfang Rongxun Building Materials Co., Ltd., China;<br>Zhongheng new material technology Co., Ltd.
Glass fiber insulation cotton (CFIC)	0.035	200	700	Hebei New Building Materials, China;<br>Weifang Codam Insulation Material Co., Ltd.<br>Shenzhen Sailing Fiberglass Co., Ltd.
Glass	0.875	2200	900	Shuyang Yanzhe Flower Gardening Center, China
High Density Polyethylene (HDPE)<br>(packaging materials of PCMs)	0.5	940[39]	2077[39]	Hebei Cangzhou Plastic Products Factory, China
Expanded polypropylene (EPP)<br>(materials of commercial thermal insulating container)	0.04	105[40]	466.7[40]	Anhui Huapai Brand Store, China
Expanded polystyrene (EPS)<br>(materials of foam covers)	0.03	40.6[41]	1280[41]	/

Supplementary Table 4|Thickness (along the X-axis in Supplementary Fig. 17) of the actual materials. Abbreviation: CFP, Ceramic fiber paper (Shanghai Liangluo Electromechanical Equipment Business Department, China); NAF, Nano aerogel felt (Langfang Yuncun Thermal Insulation Material Co., Ltd., China); CFAF, Carbon fiber aerogel felt (Langfang Yuncun Thermal Insulation Material Co., Ltd., China); VIP, Vacuum insulation panel (Langfang Rongxun Building Materials Co., Ltd., China); CFIC, Glass fiber insulation cotton (Hebei New Building Materials, China). They were calculated by Eq. (S86).

Zones	$\mathrm{R}_{\{1,1\}}$ $(\mathrm{L}_{\{1,1\}})$	$\mathrm{R}_{\{1,2\}}$ $(\mathrm{L}_{\{1,2\}})$	$\mathrm{R}_{\{1,3\}}$ $(\mathrm{L}_{\{1,3\}})$
Material [thickness (mm)]	NAF [10]	CFAF [2+2]+CFIC [3+3]	CFIC [3]+VIP [7]
Zones	$\mathrm{R}_{\{2,1\}}$ $(\mathrm{L}_{\{2,1\}})$	$\mathrm{R}_{\{2,2\}}$ $(\mathrm{L}_{\{2,2\}})$	$\mathrm{R}_{\{2,3\}}$ $(\mathrm{L}_{\{2,3\}})$
Material [thickness (mm)]	CFIC [3]+VIP [7]	NAF [10]	CFIC [3]+VIP [7]
Zones	$\mathrm{R}_{\{3,1\}}$ $(\mathrm{L}_{\{3,1\}})$	$\mathrm{R}_{\{3,2\}}$ $(\mathrm{L}_{\{3,2\}})$	$\mathrm{R}_{\{3,3\}}$ $(\mathrm{L}_{\{3,3\}})$
Material [thickness (mm)]	VIP [10]	NAF [10]	CFP [5]+VIP [5]

Supplementary Table 5|Volume of normal-temperature water (V_{nor}) for each glassware [calculated by Eq. (S88)].

Zone	①	④	⑦
Volume (mL)	23	152	220
Zone	②	⑤	⑧
Volume (mL)	49	204	158
Zone	③	⑥	⑨
Volume (mL)	76	255	95

Supplementary Table 6|Maximum, average, and minimum temperatures in the temperature control zone for the model in Fig. 3a. Case 1 pertains to a size at the "mm" level, whilst case 2 is at the "m" level.

Case	Temperature (K)	Zone		
		1	2	3
1	Maximum	303.22	285.72	280.22
	Average	303.15	285.65	280.15
	Minimum	303.08	285.58	280.08
2	Maximum	303.22	285.72	280.22
	Average	303.15	285.65	280.15
	Minimum	303.08	285.58	280.08

Supplementary References

1. Liu, F., Jia, B., Li, Z. & Zhang, X. Thermodynamics analysis for forced air pre-cooling of cherry. *J. Food Process Eng.* **44**, e13881 (2021).

2. Khumalo, G., Goedhals-Gerber, L. L., Cronje, P. & Berry, T. The non-conformance of in-transit citrus container shipments to cold protocol markets: A systematic literature review. *Food Control* **125**, 107947 (2021).

3. Jelle, B. P. Traditional, state-of-the-art and future thermal building insulation materials and solutions - Properties, requirements and possibilities. *Energy Build.* **43**, 2549-2563 (2011)

4. Cuce, E., Cuce, P. M., Wood, C. J. & Riffat, S. B. Toward aerogel based thermal superinsulation in buildings: A comprehensive review. *Renew. Sust. Energ. Rev.* **34**, 273-299 (2014).

5. Maxwell-Garnett, J. C. Colours in metal glasses and in metallic films. *Philos. Trans. R. Soc. A.* **203**, 385-420 (1904).

6. Bruggeman, D. A. G. Calculation of various physics constants in heterogenous substances I Dielectricity constants and conductivity of mixed bodies from isotropic substances. *Ann. Phys.-Berlin* **24**, 636-664 (1935).

7. Tian, B., Wang, J., Dai, G., Ouyang, X. & Huang, J. Thermal metadevices with geometrically anisotropic heterogeneous composites. *Int. J. Heat Mass Transf.* **174**, 121312 (2021).

8. Chen, Y., Xu, C., Cong, R., Ran, F. & Fang, G. Thermal properties of stearic acid/active aluminum oxide/graphene nanoplates composite phase change materials for heat storage. *Mater. Chem. Phys.* **269**, 124747 (2021).

9. Mishra, R. K., Verma, K., Mishra, V. & Chaudhary, B. A review on carbon-based phase change materials for thermal energy storage. *J. Energy Storage* **50**, 104166 (2022).

10. Zhao, Y., Zhang, X. & Hua, W. Review of preparation technologies of organic composite phase change materials in energy storage. *J. Mol. Liq*. **336**, 115923 (2021).

11. Zhou, B. et al. Novel composite phase change material of high heat storage and photothermal conversion ability. *J. Energy Storage* **49**, 104101 (2022).

12. Yu, K., Liu, Y. & Yang, Y. Review on form-stable inorganic hydrated salt phase change materials: Preparation, characterization and effect on the thermophysical properties. *Appl. Energy* **292**, 116845 (2021).

13. Fu, W. et al. High power and energy density dynamic phase change materials using pressure-enhanced close contact melting. *Nat. Energy* **7**, 270-280 (2022).

14. Woods, J. et al. Rate capability and Ragone plots for phase change thermal energy storage. *Nat. Energy* **6**, 295-302 (2021).

15. Yang, L. et al. A comprehensive review on sub-zero temperature cold thermal energy storage materials, technologies, and applications: State of the art and recent developments. *Appl. Energy* **288**, 116555 (2021).

16. Nazari, M. A. et al. A review of nanomaterial incorporated phase change materials for solar thermal energy storage. *Sol. Energy* **228**, 725-743 (2021).

17. Williams, J. D. & Peterson, G. P. A review of thermal property enhancements of low-temperature nano-enhanced phase change materials. *Nanomaterials* **11** (2021).

18. Shen, C. et al. Shape-stabilized hydrated salt/paraffin composite phase change materials for advanced thermal energy storage and management. *Chem. Eng. J.* **385**, 123958 (2020).

19. Zou, T. et al. Super absorbent polymer as support for shape-stabilized composite phase change material containing $Na_2HPO_4 \cdot 12H_2O$-$K_2HPO_4 \cdot 3H_2O$ eutectic hydrated salt. *Sol. Energy Mater. Sol. Cells* **231**, 111334 (2021).

20. Zhou, X., Zhang, X. & Zheng, Q. Composite phase change materials with heat transfer self-enhancement for thermal energy storage. *Sol. Energy Mater. Sol. Cells* **217**, 110725 (2020).

21. Liu, L. et al. Development of low-temperature eutectic phase change material with expanded graphite for vaccine cold chain logistics. *Renew. Energy* **179**, 2348-2358 (2021).

22. Xu, X. & Zhang, X. Simulation and experimental investigation of a multi-temperature insulation box with phase change materials for cold storage. *J. Food Eng.* **292**, 110286 (2021).

23. Zhao, Y., Zhang, X., Xu, X. & Zhang, S. Development of composite phase change cold storage material and its application in vaccine cold storage equipment. *J. Energy Storage* **30**, 101455 (2020).

24. Liaoning Market Supervision and Administration Bureau. Technical management specification for food cold chain logistics (warehousing and distribution). (local specification of Liaoning province, China, DB21/T 3356-2020, 2020).

25. Badshah, M. A., Leung, E. M., Liu, P., Strzelecka, A. A. & Gorodetsky, A. A. Scalable manufacturing of sustainable packaging materials with tunable thermoregulability. *Nat. Sustain.* **5**, 434-443 (2022).

26. Wen, J. Optimum temperature for various foods. *Health Protection and Promotion*, 57 (2011).

27. Liu, J. Drinking Chinese medicine doesn't have to be "while it's hot." *Health Guide*. **18**, 39 (2012).

28. Zhou, F. The best temperature for home life. *Popular Science*, 37 (1996).

29. http://cn.comsol.com/ (visible on Aug. 11, 2023).

30. Yang, S. & Tao, W. *Heat transfer* (Higher Education Press, Beijing, ed. 4, 2006).

31. https://www.matweb.com/ (visible on Aug. 11, 2023).

32. Banerjee, S., Pattnayek, S., Kumar, R. & Kar, K. K. Impact of graphite on thermomechanical, mechanical, thermal, electrical properties, and thermal conductivity of HDPE/copper composites. *Fuel Cells* **20**, 116-130 (2020).

33. Jankowski, N. R. & McCluskey, F. P. A review of phase change materials for vehicle component thermal buffering. *Appl. Energy* **113**, 1525-1561 (2014).

34. Yadav, C. & Sahoo, R. R. Effect of nano-enhanced PCM on the thermal performance of a designed cylindrical thermal energy storage system. *Exp. Heat Transf.* **34**, 356-375 (2021).

35. Li, G., Hwang, Y. & Radermacher, R. Review of cold storage materials for air conditioning application. *Int. J. Refrig.-Rev. Int. Froid.* **35**, 2053-2077 (2012).

36. Bonales, L. J., Rodríguez, A. C. & Sanz, P. D. Thermal conductivity of ice prepared under different conditions. *Int. J. Food Prop.* **20**, S610-S619 (2017).

37. Nichols, E. L. On the density of ice. *Phys. Rev. (Series I)* **8**, 21-37 (1899).

38. Wang, X. *Base research on the application of nanoporous* SiO_2 *aerogel based thermal insulation composite* (National University of Defense Technology, 2006).

39. Qi, C., Wang, J. & Yadama, V. Heat transfer modeling of oriented sorghum fibers reinforced high-density polyethylene film composites during hot-pressing. *Polymers* **13**, 3631 (2021).

40. Záleská, M. et al. Influence of waste plastic aggregate and water-repellent additive on the properties of lightweight magnesium oxychloride cement composite. *Appl. Sci.-Basel.* **9**, 5463 (2019).

41. Blaine, R. L. In search of thermal effusivity reference materials. *J. Therm. Anal. Calorim.* **132**, 1419-1422 (2018).
